# COMA: efficient structure-constrained molecular generation using contractive and margin losses

**DOI:** 10.1186/s13321-023-00679-y

**Published:** 2023-01-19

**Authors:** Jonghwan Choi, Sangmin Seo, Sanghyun Park

**Affiliations:** 1 Department of Computer Science, Yonsei University, Yonsei-ro 50, 03722 Seoul, Republic of Korea; 2 UBLBio Corporation, Yeongtong-ro 237, 16679 Suwon, Gyeonggi-do Republic of Korea

**Keywords:** Drug design, Molecular optimization, Goal-directed molecular generation, Structure-constrained molecular generation, Deep generative model, Metric learning, Contrastive learning, Reinforcement learning

## Abstract

**Background:**

Structure-constrained molecular generation is a promising approach to drug discovery. The goal of structure-constrained molecular generation is to produce a novel molecule that is similar to a given source molecule (e.g. hit molecules) but has enhanced chemical properties (for lead optimization). Many structure-constrained molecular generation models with superior performance in improving chemical properties have been proposed; however, they still have difficulty producing many novel molecules that satisfy both the high structural similarities to each source molecule and improved molecular properties.

**Methods:**

We propose a structure-constrained molecular generation model that utilizes contractive and margin loss terms to simultaneously achieve property improvement and high structural similarity. The proposed model has two training phases; a generator first learns molecular representation vectors using metric learning with contractive and margin losses and then explores optimized molecular structure for target property improvement via reinforcement learning.

**Results:**

We demonstrate the superiority of our proposed method by comparing it with various state-of-the-art baselines and through ablation studies. Furthermore, we demonstrate the use of our method in drug discovery using an example of sorafenib-like molecular generation in patients with drug resistance.

**Supplementary Information:**

The online version contains supplementary material available at 10.1186/s13321-023-00679-y.

## Introduction

**Fig. 1:**
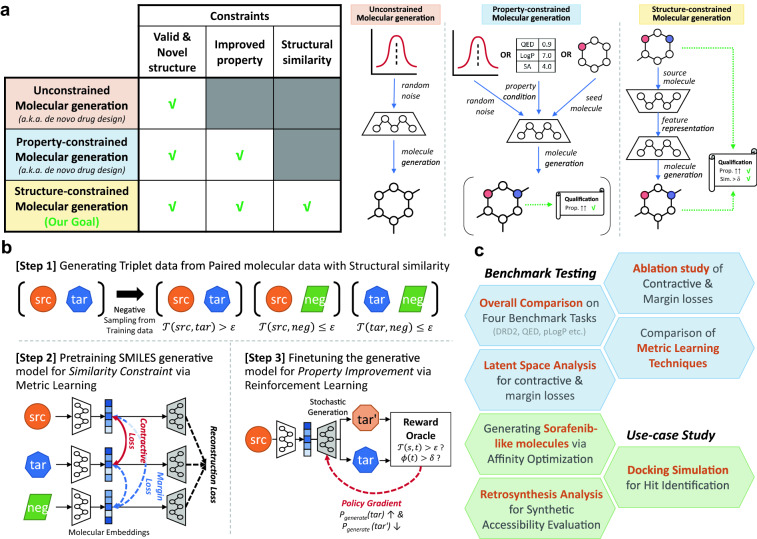
Overviews of structure-constrained molecular generation and COMA. **a** Descriptions of three types of goal-directed molecular optimisation and examples. **b** Overviews of model architecture and training scheme of COMA. The generative model of COMA is based on a variational autoencoder, and the training process consists of following two steps: metric learning with contractive and margin losses to achieve the structural similarity constraint and reinforcement learning to produce molecules satisfying both constraints of structural similarity and property improvement. **c** Overview of experiments. The goals of four benchmark tasks are to enhance each molecular property score, and the goal of use case study for sorafenib resistance is to decrease an affinity score against the ABCG2 protein

Structure-constrained molecular generation is a challenging problem in goal-directed molecular optimisation studies [1]. The goal of structure-constrained molecular generation is to produce novel molecules with improved target chemical properties while resembling the molecular structure of the source drugs (Fig. 1a). A traditional approach in organic chemistry involves the identification of a molecular substructure that associates with target biological entities (e.g. kinase) and considers several possible modifications of molecular motifs, except for the key region, to identify a novel drug candidate with potential activity against a specific disease [2, 3]. However, this brute-force-like approach requires a considerable amount of expert knowledge and enormous cost because of the large size of the drug-like chemical space, which is estimated to be in the range of ${10}^{30}$–${10}^{60}$ [4]. To address this inefficiency problem, various computer-aided drug design methods, particularly artificial intelligence (AI) technique-based applications, have been proposed.

AI-based methods for efficient goal-directed molecular optimisation utilize various deep generative models, optimisation techniques, and molecular representation methods [5–9]. Variational autoencoder (VAE), generative adversarial network (GAN), and normalizing flow are major deep generative models for molecular generation [1]. Genetic algorithm, Bayesian optimisation, particle swarm optimisation, and reinforcement learning are widely used techniques for molecular optimisation [10]. The representative molecular representation methods exploited for molecular generation are the simplified molecular-input line-entry system (SMILES) and graph-based representation; however, recent studies have attempted to use more complex representation methods, such as self-referencing embedded strings (SELFIES) and three-dimensional fixed coordinate systems [10, 11].

Many AI-based molecular optimisation methods have been proposed for structure-constrained molecular generation against diverse goals of chemical and pharmacological properties. For instance, generative tensorial reinforcement learning (GENTRL), which identifies potent DDR1 kinase inhibitors for the treatment of renal fibrosis, has been a great achievement in drug discovery because it succeeded in finding novel nanomolar hits with improved half-maximum inhibitory concentrations using VAE, the policy gradient algorithm of reinforcement learning, and the SMILES string representation method [8]. JTVAE and VJTNN are graph-based molecular generation models that use junction-tree VAEs to efficiently learn and utilize the structural information of the source molecules [12, 13]. The authors of JTVAE and VJTNN evaluated their structure-constrained molecular generation performances in several optimisation tasks for the partition coefficient (logP), quantitative estimate of drug-likeness (QED), and biological activity against dopamine receptor D2 (DRD2) using benchmark datasets containing 34–99k molecular pairs generated from the ZINC database [12]. Copy-and-refine (CORE) strategy is a variation of VJTNN and the molecular generation process of CORE for high structural similarity consists of two steps: copying some substructures of a source molecule and refining the copied ones with predefined scaffolding trees and an adversarial learning method [14]. Modof is a graph-based molecular generation method that identifies editable sites in molecules using the graph edit distance algorithm and applies several graph edit methods, such as removal fragment prediction and child node type prediction, to improve a target property [5]. EMPIRE is a SMILES-based molecular editing method to generate a new molecule with desired scaffold structures [15]. EMPIRE first identifies the scaffold structure of an input molecule, generate molecular fragments via VAE-based and building block-based models, and produce new molecular structures that resemble the input scaffold structures by adding the generate fragments to the scaffold. EMPIRE exhibited excellent performance for retaining input molecular structures, but property improvement seemed to be limited due to simple fragment addition procedure. UGMMT is a common SMILES-based VAE model; however, the author proposed a unsupervised learning-based training scheme to address the drawback of supervised approaches that paired molecular data in sufficient amounts might be unavailable in practical tasks [16]. UGMMT utilizes a double-cycle training scheme that can be considered a dual-learning method and exhibits its superiority on DRD2 and QED optimisation tasks.

Although these state-of-the-art models have shown good molecular generation performance in terms of chemical properties and structural similarity, there is still room to increase the efficiency of molecular generation with these two constraints. Furthermore, graph-based methods have been proposed more often than SMILES-based methods in structure-constrained molecular generation studies. However, a recent comprehensive study demonstrated that there are no obvious shortcomings of SMILES-based generation methods compared to graph-based generation methods, and SMILES-based molecular optimisation may be better [17].

To this end, we propose a novel structure-constrained molecular generation model called COMA, which utilizes reinforcement learning and metric learning techniques (Fig. 1b). The proposed model has the architecture of a SMILES-based VAE and is trained with two regularization terms, contractive and margin losses, to compel structurally similar molecules to have similar latent vectors in the VAE and encourage the decoder of the VAE to generate novel molecules that resemble a source molecule efficiently. In this study, the structural molecular similarity was evaluated using the Tanimoto similarity score [18]. After training with the contractive and margin losses, COMA was fine-tuned to intensively produce molecules with desirable properties among the various generated outputs similar to the source molecules using property-based rewards and the REINFORCE algorithm [19]. We verified the superiority of COMA by comparing it with several state-of-the-art models on four benchmark datasets (Fig. 1c) and confirmed the advantages of contractive and margin losses via ablation studies. Furthermore, we demonstrated the proof-of-concept of COMA by conducting a use-case study in which we explored sorafenib-like drug candidates against drug resistance in hepatocellular carcinoma (HCC). Our contributions include the following.

- We designed a COMA molecular generation model with two novel regularization terms, named *contractive* and *margin* losses, to achieve high structural similarity and property improvement simultaneously in structure-constrained molecular generation tasks.
- We verified that COMA outperformed various molecular generation models on four benchmark datasets and demonstrated the merits of contractive and margin losses by conducting ablation studies.
- We demonstrated the proof-of-concept of COMA via the discovery task of novel compounds that were similar to sorafenib, but had desirable properties associated with drug resistance in HCC.

## Results and discussion

### Overview of COMA

The generative model of the COMA is a gated recurrent unit (GRU)-based VAE for encoding and decoding SMILES strings. SMILES describes molecular structures using ASCII codes, where each ASCII code represents a component of a molecular structure, such as an atom, bond type, or branch structure. The molecular generation process of COMA is simple: for a given source molecule, the model calculates a latent vector using a pretrained encoder with contractive and margin losses; then, the model generates a molecule using the latent vector and a decoder pretrained by reinforcement learning. The training process of COMA is as follows. An encoder learns a mapping function of SMILES strings into latent vectors using contractive and margin losses to improve the structural similarity performance. The role of *contractive* loss is to embed molecules with similar structures in close points to each other in a latent space, whereas the role of *margin* loss is to force dissimilar molecules to be placed as far as possible from each other. The decoder learns how to generate valid SMILES strings from latent vectors, whereas the encoder learns a mapping function; then, it is further trained by reinforcement learning to selectively produce SMILES strings with not only high structural similarity to source molecules but also improved target properties. Details of the training process are provided in the Additional file 1: Fig. S1, and Algorithms S1–S2).

### Overall comparison on four benchmark tasks

*Dataset* To evaluate the performance of the COMA, we used four benchmark datasets (DRD2, QED, pLogP04, and pLogP06) provided in [13]. The goal of the DRD2 task is to generate a novel molecule that is more active toward dopamine receptor D2 than a source molecule under the constraint that Tanimoto similarity $\ge 0.4$. The score of biological activity against the dopamine receptor D2 has a range of [0,1], and it is interpreted that the higher the score, the better the activity. The goal of the QED task is to produce novel molecules that are more drug-like than the corresponding source molecules under the constraint that the Tanimoto similarity $\ge 0.4$. The QED score has a range of [0,1] with higher scores indicating better drug-likeness. The goal of the last pLogP04 and pLogP06 tasks was to enhance penalized logP scores with similarity thresholds of 0.4 and 0.6, respectively. A penalized logP score is defined as a logP score that accounts for the ring size and synthetic accessibility [20]. Details of the statistics for the benchmark datasets are provided in Additional file 1: Table S1.

*Baseline methods* We compared COMA with seven state-of-the-art models: JTVAE, VJTNN, VJTNN+GAN, CORE, HierG2G, HierG2G + BT, and UGMMT. JTVAE is a graph-based molecular generation model that optimizes molecular properties using a Bayesian optimisation method [12]. VJTNN is a refined version of JTVAE with an added neural attention function, and VJTNN + GAN is a more refined version with adversarial training [13]. CORE is an improved version of VJTNN + GAN, which generates molecules using a copy-and-refine strategy [14]. HierG2G is a graph-based generative model that uses a hierarchical encoding scheme [7]. HierG2G+BT is an improved version of HierG2G that adds a back-translation step for data augmentation [21]. The UGMMT is a SMILES-based generative model that is trained using an unsupervised learning scheme [16].

*Evaluation metrics* We evaluated the COMA and baseline models using various evaluation metrics of structure-constrained molecular generation (Additional file 1: Tables S2–S9). These metrics allow for various aspects of comparison of generated molecular structures, such as evaluation metrics used in GuacaMol [22]. We first trained all models with the training dataset of each benchmark task, generated molecules 20 times for each source molecule in the test dataset, and then evaluated the generated molecules with seven metrics:

- *Validity* the ratio of valid SMILES strings generated from the test data
- *Novelty* the ratio of valid SMILES strings that are not in the training data
- *Property* the average of property scores of valid SMILES strings
- *Improvement* the average of the difference of property scores between generated and source SMILES strings
- *Similarity* the average of Tanimoto similarity between generated and source SMILES strings
- *Diversity* the average of pairwise Tanimoto dissimilarity between generated SMILES strings
- *Success rate* the ratio of valid and novel SMILES strings satisfying both constraint *property improvement* and *structural similarity*.

The details of the metric calculations are described in the Methods section. We evaluated COMA and all baseline models on the two benchmark datasets, DRD2 and QED, but were not able to evaluate two baseline models, including JTVAE and UGMMT, on the penalized logP datasets because of the out-of-memory problems raised by the models during experiments.

**Fig. 2:**
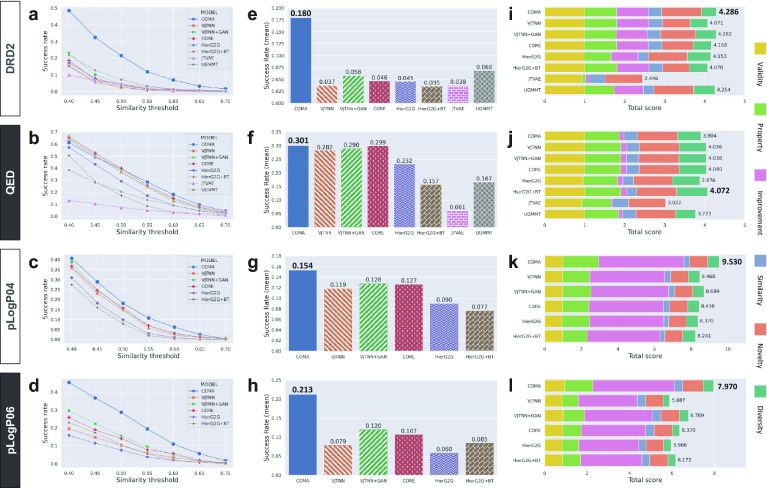
Overall performance on four benchmark tasks. **a–d** Evaluation of success rates over several similarity thresholds. For a given similarity threshold, the success rate is defined as the ratio of novel molecules satisfying both constraints property improvement and higher similarity than the threshold. **e–f** Comparison of the average scores of success rates over similarity thresholds. **i–l** Comparison of total performance scores. A total score is calculated as the sum of six metrics (validity, property, improvement, similarity, novelty, and diversity). **a**, **e**, and **i**, DRD2 results. **b**, **f**, and **j**, QED results. **c**, **g**, and **k**, Penalized LogP04 results. **d**, **h**, and **l** Penalized LogP06 results. Note that we did not evaluate two baseline models JTVAE and UGMMT on the two penalized logP datasets owing to the out-of-memory problems raised by the models during experiments

*Success Rate Comparison* We evaluated the success rate over several similarity thresholds in the range of 0.40–0.70 (Fig. 2a–h) because the success rate is the most important metric for measuring how much a model generates valid molecules satisfying three constraints simultaneously: novel structure, improved property, and structural similarity (Fig. 1a). The proposed COMA model had equivalent or better performance than the baseline models over several threshold conditions, and we confirmed that COMA was able to produce molecules satisfying high similarity constraints (0.55–0.70) more than the baseline models (Fig. 2a–d). For a quantitative comparison, we computed the average success rate over the similarity thresholds and confirmed that COMA had average scores of 0.180, 0.301, 0.154, and 0.213 for DRD2, QED, pLogP04, and pLogP06, respectively (Fig. 2e–h). Compared to the baseline models, the scores of COMA were as high as 0.002–0.240 compared to the state-of-the-art models, which demonstrate that COMA is more appropriate for structure-constrained molecular generation.

*Overall performance* The remaining six metrics show the characteristics of the COMA and baseline models (Fig. 2i–l and Additional file 1: Tables S2–S9). COMA outperformed the baseline models on all benchmark datasets in terms of validity and novelty, whereas diversity was relatively low for graph-based models, such as VJTNN and CORE. JTVAE showed better similarity performance than COMA in DRD2 and QED tasks but failed to improve property scores simultaneously, resulting in poor success rates. For the overall evaluation, we computed the total validity, properties, improvement, similarity, novelty, and diversity scores of each model (Fig. 2i–l). We confirmed that except for QED, COMA had the highest scores, which was consistent with the success rate analysis. In the deeper analysis, COMA showed the best averages of valid ratio ($0.988\pm 0.018$), property ($1.231\pm 0.457$), improvement ($2.357\pm 1.940$), and novelty ($0.988\pm 0.017$) over the four benchmark tasks, CORE had the best similarity performance ($0.344\pm 0.031$), and HierG2G produced most diverse molecular structures ($0.571\pm 0.129$). COMA was not the best models in the term of similarity, in which COMA achieved $0.341\pm 0.027$, but the similarity difference between COMA and CORE was not significantly large. HierG2G exhibited the highest average diversity over the benchmark tasks, but it had poor improvement and similarity performances of $1.922\pm 1.590$ and $0.285\pm 0.035$, respectively, resulting in the lowest average of total score of $0.932\pm 0.299$. These experimental results demonstrate that the proposed method, which is the collaboration of contractive loss, margin loss, and reinforcement learning, is effective for structure-constrained molecular generation.

**Fig. 3:**
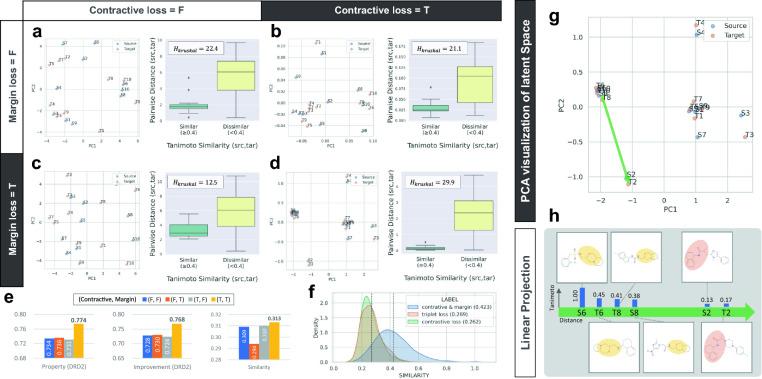
Ablation studies of contractive and margin losses. **a–d** (Left) PCA plots for 10 molecular pairs sampled from the DRD2 training dataset and (Right) comparison of pairwise distance distributions between intra-(dis)similar molecular sets that constructed from the 20 molecules. For each box plot, the center line and box limits represent the quartiles of distance distributions, and whiskers represent 1.5x interquartile range. **a** The case when neither contractive nor margin losses was used. **b** The case when only contractive loss was used. **c** The case when only margin loss was used. **d** The case when both were used. **e** Comparison of molecular generation performances for three metrics (property, improvement, and similarity) in the DRD2 benchmark task. **f** Comparison of similar molecule generation performances on the DRD2 dataset among three regularization terms, contractive-and-margin, triplet, and contrastive loss functions. The score in parentheses is the mean value of the distribution. **g** Latent space analysis in the case when both contractive and margin losses were used. The green arrow represents a projective space containing three molecular pairs for analysing the effects of contractive and margin losses. **h** Analysis of the relationship between molecular structures and embedding positions via the Tanimoto similarity. A common molecular substructure between neighboring molecules was highlighted

*Ablation Study of Contractive and Margin losses* We performed ablation experiments on the DRD2 benchmark dataset to demonstrate the advantages of the contractive and margin loss terms in achieving high similarity and property improvement simultaneously (Fig. 3).

*Statistical analysis* We examined the effects of contractive and margin losses on molecular latent vectors of VAE by examining the relationship between Tanimoto similarity and Euclidean distance over molecular pairs. First, we sampled 20 molecules from the DRD2 training dataset by randomly selecting 10 molecular pairs ${\mathcal {A}}:=\bigcup _{i=1}^{10}{\{S_i, T_i\}}$ (Additional file 1: Tables S10) and defined two sets of molecular pairs with Tanimoto similarity scores ${\mathcal {T}}$, ${\mathcal {U}}:=\{(x,y)\in {\mathcal {A}}\times {\mathcal {A}}\mid {\mathcal {T}}(x,y)\ge 0.4\}$ and ${\mathcal {V}}:=\{(x,y)\in {\mathcal {A}}\times {\mathcal {A}}\mid {\mathcal {T}}(x,y)<0.4\}$. Next, for each case of whether contractive and margin losses were used, we calculated the two-dimensional embedding vectors of the 20 molecules in ${\mathcal {A}}$ using principal component analysis (PCA), computed the Euclidean distance values of molecular pairs in ${\mathcal {U}}$ and ${\mathcal {V}}$ with the corresponding embedding vectors, and compared the distance distributions between ${\mathcal {U}}$ and ${\mathcal {V}}$ (Fig. 3a–d). The difference between the two distributions was measured using the Kruskal–Wallis H test. The high value of H statistics implies that paired molecules with similar structures are close to each other, and paired dissimilar molecules are located far away. We confirmed that the cases of usage of both contractive and margin losses had the highest H-score of 29.9 ($p<10^{-7}$). Furthermore, ideal molecular embedding distributions in a latent space were identified only when using both contractive and margin losses. The case of not using both was higher than the case of using only one of the two (Fig. 3a–c) because, in the case of only contractive loss, both pairwise distances in ${\mathcal {U}}$ and ${\mathcal {V}}$ decreased, which did not make a difference, whereas in the case of only margin loss, not only was the distance in ${\mathcal {V}}$ increased but the distance in ${\mathcal {U}}$ also increased.

*Performance Comparison* We also evaluated three metrics: property, improvement, and similarity, for each trained model with or without contractive and margin losses (Fig. 3e). There were no notable differences in the similarity between the cases, but high property and improvement scores were observed only when both regularization terms were used. These results demonstrate that contractive and margin losses play crucial roles in structure-constrained molecular generation. Furthermore, we confirmed that the combination of contractive and margin losses outperformed two traditional loss terms, triplet loss [23] and contrastive loss [24], in a similar molecule generation task on the DRD2 dataset. Both loss terms were proposed to obtain effective feature representations through metric learning. COMA generated target molecules with the average similarity of 0.423 to source molecules, whereas variations of COMA that utilized either triplet or contrastive losses instead of contractive and margin losses had the average score of 0.269 and 0.262, respectively (Fig. 3f).

*Latent space analysis* The advantage of COMA is that it elevates the similarity performance by exploiting contractive and margin losses. These terms were designed to make molecules with similar structures close to each other in a latent space and to make structurally dissimilar molecules far from each other. To verify that they worked as intended, we conducted a linear projection analysis on the data used in the previous statistical analysis (Fig. 3g–h). We selected one point S6 from the latent space, drew a randomly directed arrow starting from the point, and compared the molecular structures corresponding to the six points lying on the arrow. We confirmed that points adjacent to the starting point had a high Tanimoto similarity, whereas the far points had low similarity scores. Hence, we determined that the proposed method is as effective as intended.

### Use case: drug discovery for sorafenib resistance

Structure-constrained molecular generation can be used to discover drug candidates for patients resistant to chemotherapy by generating a novel molecule that resembles an existing drug; however, drug resistance-related chemical properties are reduced without loss of pharmacophore features of the existing drug. We applied COMA to sorafenib, a targeted anticancer drug for hepatocellular carcinoma (HCC), to enhance the therapeutic effectiveness of chemotherapy in sorafenib-resistant HCC patients.

*Association between Sorafenib Resistance and ABC transporters* Sorafenib is an inhibitor of protein kinases in the Raf/Mek/Erk pathway that suppresses cell proliferation and angiogenesis in tumour cells [25]. Owing to the moderate therapeutic effect and veiled drug resistance of sorafenib [26], the discovery of new drug candidates that can be used as alternatives to sorafenib is an important research task. One of the suspected mechanisms associated with sorafenib resistance is the ATP-binding cassette (ABC) transporters that pull drugs out of the cells [27]. Since multiple-target tyrosine kinase inhibitors (TKIs), including sorafenib, act as ABC transporter substrates [28], it appears that the ABC transporters pull out sorafenib from the HCC tumour cells before it can bind to its therapeutic target proteins. Thus, a decrease in the binding affinity of sorafenib against ABC transporter proteins without loss of affinity against the therapeutic target proteins of sorafenib may alleviate sorafenib resistance and elevate the therapeutic effectiveness of chemotherapy in HCC patients.

**Fig. 4:**
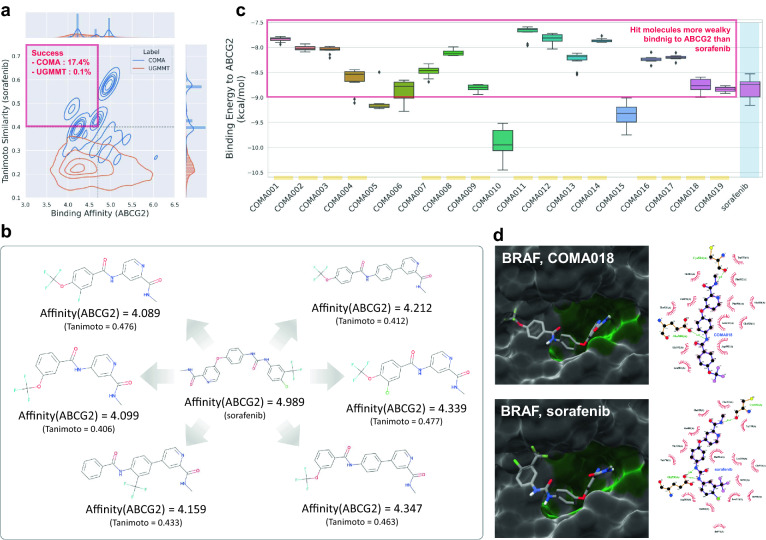
Discovery of potential drug candidates derived from sorafenib. **a** Comparison of joint distributions over binding affinity to ABCG2 (x-axis) and Tanimoto similarity to sorafenib (y-axis) using kernel density estimation between COMA and UGMMT. The success rate is defined as the affinity score against ABCG2 being less than 4.7, and the Tanimoto coefficient being greater than 0.4 simultaneously. **b** Comparison of generated molecules achieving weak ABCG2 affinity and high similarity with sorafenib. The affinity scores were evaluated by DeepPurpose. **c** Comparison of binding energies of sorafenib and 19 molecules satisfying the constraints. The binding energy scores to ABCG2 were evaluated using AutoDock Vina. For each box, the center line and box limits shows the quartiles of binding energy to ABCG2, and whiskers represent 1.5x interquartile range. The 15 of 19 molecules have higher binding energies with ABCG2 compared to sorafenib, which makes them potential drug candidates for alleviating sorafenib resistance. **d** Docking simulation results of sorafenib and the identified hit molecule COMA018 against BRAF. (Left) 3D visualization of ABCG2 and ligand complexes drawn by Chimera to show a binding pocket and contact regions. (Right) 2D visualization of complexes drawn by LigPlot Plus to show binding sites and residues

*Binding Affinity Optimisation against ABCG2* To conduct a proof-of-concept of COMA for sorafenib-like hit discovery, we designed a goal to decrease the binding affinity score against the protein of ABC subfamily G member 2 (ABCG2) while conserving the substructures of sorafenib without loss of affinity against the serine/threonine-protein kinase B-raf (BRAF), which is a target kinase of sorafenib (Additional file 1: Table S11). We curated 16k SMILES strings from the ChEMBL database [29] and constructed training datasets for the COMA and UGMMT (Additional file 1: Table S1 and Algorithm S3). We selected UGMMT as a baseline model because, to the best of our knowledge, it is the state-of-the-art SMILES-based model. After training and generating 10k molecules with sorafenib as the source molecule, we compared the success rates (Fig. 4a). The success rate was defined as the ratio of generated novel molecules satisfying the target molecule conditions (Tanimoto similarity $>0.4$ and affinity score to ABCG2 $<4.7$). COMA exhibited a high success rate (0.174), whereas UGMMT had a low success rate (0.001). The UGMMT score was too low because UGMMT failed to generate molecules similar to sorafenib, although UGMMT reduced the binding affinity against ABCG2 more than COMA. We confirmed that the outputs of COMA not only exhibited high Tanimoto similarities but also had molecular structures that resembled sorafenib to the human eye (Fig. 4b).

*Docking simulation for hit identification* To identify drug candidates for sorafenib resistance among the molecules generated by COMA, we assessed their docked poses on ABCG2 and BRAF. Docked poses were evaluated using AutoDock Vina 1.2.3 [30] and visualised using Chimera 1.16 [31] and LigPlot Plus 2.2.5 [32]. To prepare the ligands, we first obtained 19 unique molecules among the 10k generated molecules by duplicated molecule removal (Additional file 1: Table S12 and Fig. S3), generated three-dimensional coordinates of molecules using Open Babel 3.1.1 [33], (de)protonated molecules at pH 7.4, and made pdbqt files using meeko 0.3.0, which is a Python library for AutoDock. To prepare the ABCG2 and BRAF receptors, we downloaded 3D structure files, including 6VXH and 1UWH, from the PDB database [34] for ABCG2 and BRAF, respectively, and checked their hydrogens using ADFR software 1.0 [35]. We utilized Chimera to define the box center and size and executed AutoDock Vina to generate 20 poses per receptor-ligand pair. We selected the best pose with the lowest score per receptor-ligand pair and compared it with that of sorafenib.

We found that 15 molecules had lower binding energies against ABCG2 than sorafenib (Fig. 4c); hence, these molecules might be hit molecules as alternatives to sorafenib. Next, to check whether the hit candidates had as strong a binding affinity against BRAF as sorafenib, we drew graphics for the 3D structure of the receptor-ligand complex using Chimera and confirmed that these molecules fit well with the binding pocket for sorafenib in BRAF. (Fig. 4d and Additional file 1: Fig. S4). Furthermore, we confirmed that the generated molecules and sorafenib had common interatomic contacts based on the van der Waals radii using the structural analysis tools and default parameters. The 2D plots drawn by LigPlot Plus also showed that the molecules had hydrogen bonds with residues, including Glu500(A) and Asp593(A), interacting with sorafenib in BRAF.

*Synthetic accessibility evaluation* Finally, we evaluated the synthesizability of the molecules generated by COMA using the retrosynthesis analysis of Scifinder-n [36]. Most molecules could be synthesised in two steps (Additional file 1: Fig. S5). Because the generated molecules were similar to the existing drug sorafenib, good synthesizability could be guaranteed, which suggests that structure-constrained molecule generation models, such as COMA, would be effective tools in practical tasks for goal-directed drug discovery. Taken together, the results of the *in silico* analysis demonstrate that sorafenib derivatives generated by COMA can be alternative drug candidates to sorafenib in patients exhibiting high drug resistance.

## Conclusion

AI-based generative models for structure-constrained molecular generation can not only be a solution for effective drug discovery but also a powerful and explainable tool for chemists and pharmacologists. Existing structure-constrained molecular generation models exhibit good performance in terms of generating molecules with better molecular properties than source compounds; however, they have limitations in producing molecules that satisfy molecular property improvement, novelty, and high similarity to the source molecule simultaneously. In this study, we designed a VAE-based generative model for structure-constrained molecular generation with two novel regularization terms of metric learning: *contractive* and *margin* losses. Our model achieved both high property improvement and high structural similarity via two training phases: a metric learning phase that enables the VAE to get the ability to generate molecules similar to the source molecule and a reinforcement learning phase that makes the VAE intensively produce molecules satisfying both the similarity constraint and property enhancement. COMA outperformed various state-of-the-art models on four benchmark datasets: DRD2, QED, plogP04, and plogP06. Ablation studies demonstrated the importance of contractive and margin losses. Furthermore, we introduced the use case of COMA in drug discovery for sorafenib resistance.

COMA exploited reinforcement learning framework with heuristically customized reward functions for each benchmark task. While the importance of reward function design in reinforcement learning is well-known [37], there is no standard method to find optimal reward functions yet, which could make COMA practical applications difficult. We recommend using the reward functions designed in this study as prototypes and optimize them by slightly changing the reward parameters such as the threshold value of similarity.

In this study, we used the metric learning framework for structure-constrained molecular optimisation, which allows to improve a target property with retaining some molecular structures, but recent drug design studies devote their efforts to multi-objective molecular optimisation, which might be a more challenging task. In the future, to address the multi-objective molecular optimisation tasks, we will attempt to develop new applications of our proposed contractive and margin loss terms by replacing structure similarity by biological activity similarity.

## Methods

### Implementation details

COMA was implemented using Python 3.6, and several open-source tools, including PyTorch 1.10.1 and RDKit 2021.03.5. RDKit, an open source tool for chemoinformatics, was used for SMILES kekulization, SMILES validity check, Tanimoto similarity computation, and estimation of QED. PyTorch, an open-source machine learning framework, was used to construct and train the neural networks of COMA. All experiments were conducted on Ubuntu 18.04.6 LTS with 64 GB of memory and a GeForce RTX 3090.

### Tanimoto similarity

The Tanimoto similarity, which ranges from 0 to 1, compares molecular structures such as atom pairs and topological torsions, represented by Morgan fingerprints. In this study, the Morgan fingerprints were binary vectors generated using RDKit with radii of 2 and 2048 bits. For any two SMILES strings *x* and *y* with the corresponding fingerprint vectors $FP(x)=(p_1, p_2,..., p_{2048})$ and $FP(y)=(q_1, q_2,..., q_{2048})$, the Tanimoto similarity score was computed as:

1 $$\begin{aligned} {\mathcal {T}}(x,y)=\frac{\sum \limits _{i=1}^{2048}{p_i q_i}}{\sum \limits _{j=1}^{2048}{(p_j + q_j - p_j q_j)}}. \end{aligned}$$

### Binding affinity prediction

Predicting the binding affinity scores for ABCG2 and BRAF is crucial for the application of COMA for sorafenib resistance. In this study, DeepPurpose [38], a PyTorch-based library for virtual screening, was used for the accurate and high-throughput affinity prediction of more than 4.6 million pairs of molecules. We exploited the predictive model with message-passing and convolutional neural networks pretrained on BindingDB, which is a public database of measured binding affinities [39], to generate training datasets for UGMMT and COMA and compute the rewards of reinforcement learning in COMA.

### Benchmark datasets

In this study, we used four benchmark datasets provided in [13] and the original dataset for sorafenib resistance (Additional file 1: Table S1).

The DRD2 dataset contains 34k molecular pairs (source and target) with DRD2 activity scores derived from the ZINC database [40]. The DRD2 activity score ranged from 0 to 1 and was assessed using the regression model of support vector machine from [41]. For each pair in the DRD2 dataset, the pair of SMILES strings satisfied the similarity constraint that the Tanimoto similarity was greater than or equal to 0.4, and the DRD2 scores of the source and target SMILES strings were less than 0.05 and greater than 0.5, respectively. The QED dataset contained 88k molecular pairs derived from the ZINC database with QED scores. The QED score ranged from 0 to 1 and was calculated using the RDKit [42]. For each pair in the QED dataset, the Tanimoto similarity between two SMILES strings was greater than or equal to 0.4, and the QED scores of the source and target were in the ranges [0.7, 0.8] and [0.9, 1.0], respectively. The penalized logP04 and penalized logP06 datasets contained 98k and 74k molecular pairs derived from the ZINC database with penalized logP scores, respectively. The penalized logP score ranged from $-$63.0 to 5.5. For each pair in the penalized logP04 dataset, the Tanimoto similarity between two SMILES strings was greater than or equal to 0.4. In the case of the penalized logP06, the similarity threshold was set to 0.6.

We constructed a dataset for the sorafenib-like molecular generation to introduce an example of COMA application. Based on the observation that the activity of ABCG2 is related to sorafenib resistance in hepatocellular carcinoma [27, 43], this application aimed to generate sorafenib-like molecules with lower binding affinity against ABCG2, while conserving the level of affinity against the target kinase of sorafenib BRAF as much as possible. The dataset contained 231k molecular pairs derived from the ChEMBL database [29] with binding affinity scores against ABCG2 and BRAF. The binding affinity score evaluated using DeepPurpose was pKd. For each pair in the ABCG2 dataset, the Tanimoto similarity between two molecules was greater than or equal to 0.4, and the ABCG2 affinity values of the source and target were in the ranges [4.9, 8.4] and [3.3, 4.7], respectively. For BRAF, both the source and target had binding affinities greater than 6.0.

### Metric learning for molecular structural similarity

COMA has the structure of VAEs. It consists of an encoder $q_\phi$ that maps an input SMILES string to a latent vector, and decoder $p_\psi$ that reconstructs a string from a latent vector. However, the goal of COMA is to generate a SMILES string that is structurally similar to a given input SMILES string, whereas the original VAE aims to sample a random SMILES string. More concretely, given a pair of SMILES strings with similar chemical structures $x_{s}$ and $x_{t}$, the objective function of COMA $L(x_{s}, x_{t})$ is expressed as

2 $$\begin{aligned} \begin{aligned} L(x_{s}, x_{t})&= \,{\mathbb {E}}_{z \sim q_{\phi }(z \mid x_{t})}{[logp_{\psi }(x_{t} \mid z)]} \\&+ {\mathbb {E}}_{z \sim q_{\phi }(z \mid x_{s})}{[logp_{\psi }(x_{s} \mid z)]} \\&- D(q_{\phi }(z \mid x_{t}) \Vert p(z \mid x_{s})) \\&- D(q_{\phi }(z \mid x_{s}) \Vert p(z \mid x_{t})), \end{aligned} \end{aligned}$$

where $p(z \mid x_{s})$, $p(z \mid x_{t})$ are prior distributions, and $D(\cdot \Vert \cdot )$ is the Kullback–Leibler (KL) divergence. Equation (2) was derived from the lower bound of $logp(x_{t} \mid x_{s}) + logp(x_{s} \mid x_{t})$. The prior distributions were replaced by $q_{\phi }(z \mid x_{s})$ and $q_{\phi }(z \mid x_{t})$, respectively, because our assumption for similarity was that paired SMILES strings should be embedded into an identical latent point; thus, the KL terms ensured that $q_{\phi }(z \mid x_{s})$ and $q_{\phi }(z \mid x_{t})$ were equal. However, the computation of double KL terms is complex and unstable because the gradients must be calculated for both trainable distributions, that is, $q_{\phi }(z \mid x_{s})$ and $q_{\phi }(z \mid x_{t})$, in KL terms. To replace the KL terms for efficient computation, we utilized the Fréchet distance [44] that measures the distances between the probability distributions. The objective function of the COMA in Eq(2) is rewritten as

3 $$\begin{aligned} \begin{aligned} L(x_{s}, x_{t})&= \,{\mathbb {E}}_{z \sim q_{\phi }(z \mid x_{t})}{[-logp_{\psi }(x_{t} \mid z)]} \\&+ {\mathbb {E}}_{z \sim q_{\phi }(z \mid x_{s})}{[-logp_{\psi }(x_{s} \mid z)]} \\&+ \Vert \mu _{t} - \mu _{s}\Vert ^2 + tr[\Sigma _{t} + \Sigma _{s} - 2(\Sigma _{t}\Sigma _{s})^{1/2}], \end{aligned} \end{aligned}$$

where $\mu _{t}$ and $\Sigma _{t}$ are the mean vector and covariance matrix, respectively, of a target SMILES string $x_{t}$ computed by encoder $q_\phi$, and $\mu _{s}$ and $\Sigma _{s}$ are the mean vector and covariance matrix, respectively, of a source string $x_{s}$.

Equation (3) can impose the restriction that similar SMILES strings have identical distributions; however, it is not guaranteed that structurally dissimilar strings have different distributions. To address this issue, triplet learning was designed to create effective molecular embeddings for (dis)similar molecular pairs. The algorithm for the triplet dataset construction from a paired dataset is described in Additional file 1: Algorithm S3. Given a triplet of SMILES strings ($x_{s}$, $x_{t}$, $x_{n}$), where $x_{s}$ and $x_{t}$ are similar but $x_{n}$ is relatively different, the final objective function of COMA $L(x_{s}, x_{t}, x_{n})$ is expressed as

4 $$\begin{aligned} \begin{aligned} L(x_{s}, x_{t}, x_{n})&= \,{\mathbb {E}}_{z \sim q_{\phi }(z \mid x_{t})}{[-logp_{\psi }(x_{t} \mid z)]} \\&+ {\mathbb {E}}_{z \sim q_{\phi }(z \mid x_{s})}{[-logp_{\psi }(x_{s} \mid z)]} \\&+ \underbrace{{\mathbb {E}}_{z \sim q_{\phi }(z \mid x_{n})}{[-logp_{\psi }(x_{n} \mid z)]}}_\text {Reconstruction loss} \\&+ \underbrace{\Vert \mu _{t} - \mu _{s}\Vert ^2 + tr[\Sigma _{t} + \Sigma _{s} - 2(\Sigma _{t}\Sigma _{s})^{1/2}]}_\text {Contractive loss} \\&+ softplus(1 - \Vert \mu _{t} - \mu _{n}\Vert ^2) \\&+ \underbrace{softplus(1 - \Vert \mu _{s} - \mu _{n}\Vert ^2)}_\text {Margin loss}, \end{aligned} \end{aligned}$$

where $softplus(x)=log(1+e^x)$. The softplus function was used to prevent the excessive spread of latent vectors. In Eq. (4), the role of *reconstruction loss* was that the encoder and decoder learned how to generate valid and diverse SMILES strings, and both the *contractive loss*, which was equal to the Fréchet distance between two multivariate Gaussian distributions, and the *margin loss*, which was similar to soft hinge loss, were aimed at improving the performance of structure-constrained molecular generation in terms of similarity. COMA was first trained using these three loss terms (Additional file 1: Algorithm S2), and then it was trained via the reinforcement learning procedure explained below.

### Reinforcement learning for molecular property optimisation

COMA can translate an input molecule into a valid SMILES string that resembles the input via metric learning; however, the outputs of COMA are not yet guaranteed to exhibit desirable chemical properties. Reinforcement learning aims to encourage COMA to produce molecules that are structurally similar to the given input molecule, but with desirable chemical properties. To achieve this goal, we utilized the REINFORCE algorithm [19], where an episode and a reward are defined as the transformation process of an input SMILES string and a score based on the evaluated properties of the output, respectively. Specifically, for a given SMILES string *x* and a given reward function *R*, the objective function is expressed as

5 $$\begin{aligned} L(x) = -R({\hat{x}},x)p_{\psi }({\hat{x}} \mid z_{x}), \end{aligned}$$

where ${\hat{x}}$ is an output translated from *x*, and $z_{x}$ is a latent vector of *x* sampled by the pretrained encoder $q_\phi$. Because generating molecules with desirable properties depends solely on the decoder’s ability, only the gradient of the decoder was calculated as follows:

6 $$\begin{aligned} \nabla _{\psi }L(x) = {\mathbb {E}}_{p_{\psi }({\hat{x}} \mid z_{x})}[-R({\hat{x}},x)\nabla _{\psi }logp_{\psi }({\hat{x}} \mid z_{x})]. \end{aligned}$$

In Eqs. (5) and (6), a reward function $R({\hat{x}},x)$ should be designed for property improvement while ensuring that the degeneration of the similarity between ${\hat{x}}$ and *x* is within the allowable limits. In the case of DRD2 and QED, the reward function was defined as

7 $$\begin{aligned} R({\hat{x}},x) = {\left\{ \begin{array}{ll} \max \{0,\frac{\phi ({\hat{x}}) - \delta }{1 - \delta }\} & \quad \text {if } {\mathcal {T}}({\hat{x}},x) > \epsilon \\ 0 &\ quad \text {otherwise}, \end{array}\right. } \end{aligned}$$

where ${\mathcal {T}}$ is a function for the Tanimoto similarity, $\phi$ is a property scoring function, $\delta$ is the threshold of the property, and $\epsilon$ is the threshold of the Tanimoto coefficient. Using this reward function, the decoder of COMA was guided to increase the probability of producing molecules with property $>\delta$ and similarity $>\epsilon$. In this study, we set heuristically $\delta$ as 0 and 0.75 for DRD2 and QED, respectively, and $\epsilon =0.3$ in both tasks. In the pLogP04 and pLogP06 tasks, a slightly modified reward function is exploited.

8 $$\begin{aligned} R({\hat{x}},x) = {\left\{ \begin{array}{ll} \max \{0,\frac{[\phi ({\hat{x}}) - \phi (x)] - \delta }{1 - \delta }\} & \quad  \text {if } {\mathcal {T}}({\hat{x}},x) > \epsilon \\ 0 & \quad  \text {otherwise}, \end{array}\right. } \end{aligned}$$

We set threshold of property improvement $\delta$ as 0 for both pLogP04 and pLogP06, and a similarity threshold $\epsilon$ was set 0.3 and 0.5 for pLogP04 and pLogP06, respectively. In contrast, in the case of decreasing property values, such as the affinity of ABCG2, the reward function was defined as:

9 $$\begin{aligned} R({\hat{x}},x) = {\left\{ \begin{array}{ll} \max \{0,\frac{\delta - \phi ({\hat{x}})}{\delta }\} & \quad \text {if } {\mathcal {T}}({\hat{x}},x) > \epsilon \\ 0 & \quad \text {otherwise}, \end{array}\right. } \end{aligned}$$

Using this reward function, the decoder of COMA could receive a positive reward only if a molecule with property $<\delta$ and similarity $>\epsilon$ was generated. In the experiment for sorafenib resistance, $\epsilon$ was set to 0.4, $\delta =4.989$, and we added a condition for a positive reward in which the affinity value against BRAF was larger than 6.235. The two threshold values of 4.989 and 6.235 were equal to the affinity values of sorafenib against ABCG2 and BRAF, respectively, as evaluated using DeepPurpose.

The details of the reinforcement learning procedure for COMA are described in the Additional file 1: Algorithm S3.

### Evaluation Metrics

To evaluate the performance in structure-constrained molecular generation tasks, we used the following seven metrics: validity, novelty, property, improvement, similarity, diversity, and success rate. More concretely, given the training and test datasets $X_{train}$ and $X_{test}$, the seven metrics for a molecular generation model ${\mathcal {M}}$ that generates 20 molecules with one source molecule were defined as

10 $$\begin{aligned} Valid({\mathcal {M}})= & {} \sum _{x \in X_{test}}{\frac{{1\!\!1}\left( \sum _{y \in {\mathcal {M}}(x)}{\zeta (y)}>0\right) }{|X_{test} |}}, \end{aligned}$$

11 $$\begin{aligned} Novel({\mathcal {M}})= & {} \sum _{x \in X_{test}}{\frac{{1\!\!1}\left( {\mathcal {M}}(x)\setminus X_{train}\ne \emptyset \right) }{|X_{test} |}}, \end{aligned}$$

12 $$\begin{aligned} Prop({\mathcal {M}})= & {} \frac{1}{|X_{test} |} {\sum _{x \in X_{test}}{\left( \frac{\sum _{y \in {\mathcal {M}}(x)}{\zeta (y)\phi (y)}}{\sum _{y \in {\mathcal {M}}(x)}{\zeta (y)}}\right) }}, \end{aligned}$$

13 $$\begin{aligned} Impr({\mathcal {M}})= & {} \frac{1}{|X_{test} |} {\sum _{x \in X_{test}}{\left( \frac{\sum _{y \in {\mathcal {M}}(x)}{\zeta (y)[\phi (y)-\phi (x)]}}{\sum _{y \in {\mathcal {M}}(x)}{\zeta (y)}}\right) }}, \end{aligned}$$

14 $$\begin{aligned} Sim({\mathcal {M}})= & {} \frac{1}{|X_{test} |} {\sum _{x \in X_{test}}{\left( \frac{\sum _{y \in {\mathcal {M}}(x)}{\zeta (y){\mathcal {T}}(y,x)}}{\sum _{y \in {\mathcal {M}}(x)}{\zeta (y)}}\right) }}, \end{aligned}$$

15 $$\begin{aligned} Div({\mathcal {M}})= & \frac{1}{|X_{test} |} {\sum _{x \in X_{test}}{\left( 1- \frac{\sum _{\begin{array}{l} y \in {\mathcal {M}}(x); \\ z \in {\mathcal {M}}(x) \setminus \{y\} \end{array}}{\zeta (y)\zeta (z){\mathcal {T}}(y,z)}}{\sum _{\begin{array}{l} y \in {\mathcal {M}}(x); \\ z \in {\mathcal {M}}(x) \setminus \{y\} \end{array}}{\zeta (y)\zeta (z)}} \right) }}, \end{aligned}$$

16 $$\begin{aligned} SR({\mathcal {M}})= &  \sum _{x \in X_{test}} \frac{{ {1\!\!1}\left( \sum _{\begin{array}{c} y \in {\mathcal {M}}(x); \\ y \notin X_{train} \end{array}}{\left\{ \zeta (y)\mathbb {1}(\phi (y)-\phi (x)\ge \delta ){1\!\!1}({\mathcal {T}}(y,x)\ge \epsilon )\right\} > 0}\right) }}{{|X_{test} |}}, \end{aligned}$$

where ${1\!\!1}(p)$ is an indicator function that returns 1 if a statement *p* is true and zero otherwise; $\zeta (x)$ is an indicator function that returns 1 if an input SMILES string *x* is valid and zero otherwise; $\phi (x)$ is an oracle that calculates a property score of an input SMILES string *x*; ${\mathcal {T}}$ is a function for Tanimoto similarity, and $\delta$ and $\epsilon$ are thresholds of improvement and similarity, respectively.

### Docking simulation

To verify our findings, that is, the molecules translated from sorafenib in the application example, we analysed the binding poses and evaluated the binding energy using docking simulation tools, including AutoDock Vina [30], Chimera [31], and LigPlot Plus [32]. AutoDock Vina is one of the most widely used open-source docking programs. To evaluate the extent to which our findings had less binding affinity against ABCG2, which is associated with sorafenib resistance, we conducted a docking simulation and computed scores in terms of binding energy using AutoDock Vina. Chimera is a program for interactive three-dimensional (3D) visualisation and analysis of molecular structures. We used Chimera to check the docking poses between BRAF and several ligands, including sorafenib, and our findings. LigPlot Plus is a program used for 2D visualisation of ligand-protein interaction diagrams. Through the output figures of LigPlot Plus, we identified protein residues interacting with sorafenib and compared them to analyse whether our molecules lost their binding ability against BRAF, a therapeutic target of sorafenib.

## Supplementary Information

**Additional file 1.** Supplementary algorithms S1–S3, tables S1–S12, and figures S1–S6.

## Data Availability

All source code and datasets used to produce the conclusions of this article are available at https://github.com/mathcom/COMA.

## Abbreviations

ABC: ATP-binding cassette
ABCG2: ABC subfamilty G member 2
AI: Artificial intelligence
BT: Back translation
CORE: Copy-and-refine
DDR1: Discoidin domain receptor tyrosine kinase 1
DRD2: Dopamine receptor D2
GAN: Generative adversarial network
GENTRL: Generative tensorial reinforcement learning
GRU: Gated recurrent unit
HCC: Hepatocellular carcinoma
HierG2G: Hierarchical geneartion for graph-to-graph translation
JTVAE: Junction-tree variational autoencoder
KL: Kullback–Leibler
QED: Quantitative estimate of drug-likeness
SELFIES: Self-referencing embedded strings
SMILES: Simplified molecular-input line-entry system
TKI: Tyrosine kinase inhibitor
UGMMT: Unpaired generative molecule-to-molecule translation
VAE: Variational autoencoder
VJTNN: Variational junction-tree encoder-decoder.
