# Supplementary material for: COMA: efficient structure-constrained molecular generation using contractive and margin losses
Source: J Cheminform. 2023 Jan 19;15:8. doi: 10.1186/s13321-023-00679-y (PMC9850577; doi:10.1186/s13321-023-00679-y)
Supplement: Supplementary file 1 — Additional file 1. Supplementary algorithms S1–S3, tables S1–S12, and figures S1–S6. [file 13321_2023_679_MOESM1_ESM.pdf]

Efficient Structure-constrained Molecular Generation using Contractive and Margin Losses

- Supplementary Information -

Index

Algorithm S1. Pretraining procedure of COMA with Metric Learning · · · 3
Algorithm S2. Finetuning procedure of COMA with Reinforcement Learning · · · 4
Algorithm S3. Generating procedure of a triplet dataset from a pair dataset · · · 5
Table S1. Descriptions of datasets · · · 6
Table S2. Success rates of similarity-constrained molecular optimization on DRD2 benchmark test · · · 7
Table S3. Molecular generative performance to SOTA models on DRD2 benchmark test · · · 7
Table S4. Success rates of similarity-constrained molecular optimization on QED benchmark test · · · 8
Table S5. Molecular generative performance to SOTA models on QED benchmark test · · · 8
Table S6. Success rates of similarity-constrained molecular optimization on pLogP04 benchmark test · · · 9
Table S7. Molecular generative performance to SOTA models on pLogP04 benchmark test · · · 9
Table S8. Success rates of similarity-constrained molecular optimization on pLogP06 benchmark test · · · 10
Table S9. Molecular generative performance to SOTA models on pLogP06 benchmark test · · · 10
Table S10. List of 20 SMILES strings for principal component analysis in DRD2 · · · 11
Table S11. Information of targeted proteins in the proof-of-concept experiment · · · 12
Table S12. List of candidate molecules translated from sorafenib by COMA · · · 13
Figure S1. Overview of training procedure for COMA · · · 14
Figure S2. Loss and reward plots for COMA during pretraining and finetuning steps · · · 15
Figure S3. Comparison of molecular structures between sorafenib and candidates · · · 16
Figure S4. Docking simulation analysis of candidates · · · 23
Figure S5. Retrosynthesis analysis of candidates · · · 33
Figure S6. Examples of molecular translation via COMA · · · 40

Algorithm S1. Pretraining procedure of COMA with Metric Learning

Inputs: Initial weights of encoder φ, initial weights of decoder ψ, a training dataset $D = \{(x_{src}, x_{tar}, x_{neg})\}$, a strength of contractive loss β, a strength of margin loss γ, a learning rate η, a number of iterations T, a batch size M

Outputs: Pretrained weights of encoder φ, pretrained weights of decoder ψ

for $t = 1$ **to** T **do**
  $L \leftarrow 0$ *# initialize a loss*
  for $i = 1$ **to** M **do**
    $x_{src}, x_{tar}, x_{neg} \leftarrow$ Sampling(D) *# random sampling from training data*
    $\mu_{src}, \Sigma_{src} \leftarrow$ Encoder(x_{src}; φ)
    $\mu_{tar}, \Sigma_{tar} \leftarrow$ Encoder(x_{tar}; φ)
    $\mu_{neg}, \Sigma_{neg} \leftarrow$ Encoder(x_{neg}; φ)
    $x'_{src} \leftarrow$ Decoder(μ_{src}, Σ_{src}; ψ)
    $x'_{tar} \leftarrow$ Decoder(μ_{tar}, Σ_{tar}; ψ)
    $x'_{neg} \leftarrow$ Decoder(μ_{neg}, Σ_{neg}; ψ)
    $L \leftarrow L +$ ReconstructionLoss(x_{src}, x'_{src})
    $L \leftarrow L +$ ReconstructionLoss(x_{tar}, x'_{tar})
    $L \leftarrow L +$ ReconstructionLoss(x_{neg}, x'_{neg})
    $L \leftarrow L + \beta *$ ContractiveLoss($\mu_{src}, \Sigma_{src}, \mu_{tar}, \Sigma_{tar}$)
    $L \leftarrow L + \gamma *$ [MarginLoss(μ_{src}, μ_{neg}) + MarginLoss (μ_{tar}, μ_{neg})]
  end for
  $\Delta\varphi, \Delta\psi \leftarrow$ Adam(L, φ, ψ)
  $\varphi \leftarrow \varphi - \eta * \Delta\varphi$
  $\psi \leftarrow \psi - \eta * \Delta\psi$
end for

Algorithm S2. Finetuning procedure of COMA with Reinforcement Learning

Inputs: Pretrained weights of encoder φ, pretrained weights of decoder ψ, a training dataset $D = \{(x_{src}, x_{tar}, x_{neg})\}$, a property-based reward oracle Λ, a threshold of similarity δ, a learning rate η, a number of iterations T, a batch size M, a size of replay buffer N

Outputs: Weights of encoder φ, weights of decoder ψ

$B \leftarrow \emptyset$ *# initialize a replay buffer*
$t \leftarrow 0$
while $t < T$ **do**
    while $|B| < N$ **do**
        $x_{src}, x_{tar}, x_{neg} \leftarrow \text{Sampling}(D)$ *# random sampling from training data*
        $x'_{src} \leftarrow \text{Decoder}(\text{Encoder}(x_{src}; \varphi); \psi)$
        if $\text{Tanimoto}(x_{src}, x'_{src}) > \delta$ **do**
            $B \leftarrow B \cup \{(x_{src}, x'_{src}, \Lambda(x'_{src}))\}$
        end if
    end while
    $L \leftarrow 0$
    for $i = 1$ **to** M **do**
        $x, x', r \leftarrow \text{Sampling}(B)$ *# random sampling from the replay buffer*
        $B \leftarrow B - \{(x, x', r)\}$ *# update for sampling without replacement*
        $L \leftarrow L - \text{logp}(x' | x) * r$
    end for
    $\Delta\psi \leftarrow \text{Adam}(L, \psi)$
    $\psi \leftarrow \psi + \eta * \Delta\psi$
    $t \leftarrow t + 1$
end while

Algorithm S3. Generating procedure of a triplet dataset from a pair dataset

Inputs: a paired dataset $D = \{(x_{src}, x_{tar})\}$, a number of repetition M, a threshold of similarity ε

Output: A triplet dataset $D' = \{(x_{src}, x_{tar}, x_{neg})\}$

$D' \leftarrow \emptyset$ *# initialize a set of triplets*
$\Sigma \leftarrow \emptyset$ *# initialize a set of negative elements*
for (x_{src}, x_{tar}) **in** D **do**
  $\Sigma \leftarrow \Sigma \cup \{x_{src}, x_{tar}\}$
end for
for (x_{src}, x_{tar}) **in** D **do**
  for $i = 1$ **to** M **do**
    $x_{neg} \leftarrow$ Sampling(Σ) *# random sampling from negative data*
    if Tanimoto(x_{src}, x_{neg}) $< \varepsilon$ **and** Tanimoto(x_{tar}, x_{neg}) $< \varepsilon$ **do**
      $D' \leftarrow D' \cup \{(x_{src}, x_{tar}, x_{neg})\}$
    end if
  end for
end for

Table S1. Descriptions of datasets

		DRD2	QED	pLogP04	pLogP06	Sorafenib
Number of Unique Items	Triplets (Src,Tar,Neg)	688040	1766120	1973800	1495400	4612380
	Pairs (Src,Tar)	34402	88306	98690	74770	230619
	Src	18490	38723	57856	67718	13840
	Tar	3141	13202	44759	69762	2340
	Neg	21632	51923	99066	132397	16180
Range of Tanimoto Similarity	(Src,Tar)	0.40 – 0.83	0.40 – 0.80	0.40 – 1.00	0.60 – 1.00	0.40 – 1.00
	(Src,Neg)	0.00 – 0.30	0.00 – 0.30	0.00 – 0.30	0.00 – 0.49	0.03 – 0.30
	(Tar,Neg)	0.00 – 0.30	0.00 – 0.30	0.00 – 0.30	0.00 – 0.49	0.03 – 0.30
Range of Property	Src	0.00 – 0.05	0.70 – 0.80	-62.52 – 1.66	-32.33 – 3.89	4.90 – 8.37
	Tar	0.50 – 1.00	0.90 – 0.95	-42.76 – 4.17	-30.63 – 5.48	3.39 – 4.70
	Difference (Tar - Src)	0.45 – 1.00	0.10 – 0.25	1.00 – 64.36	1.00 – 23.79	N/A

Table S2. Success rates of similarity-constrained molecular optimization on DRD2 benchmark test

	Threshold of similarity						
	**0.40**	**0.45**	**0.50**	**0.55**	**0.60**	**0.65**	**0.70**
COMA	**0.485**	**0.324**	**0.215**	**0.118**	**0.069**	**0.032**	**0.016**
VJTNN	0.132	0.052	0.020	0.008	0.002	0.002	0.000
VJTNN+GAN	0.199	0.083	0.033	0.014	0.007	0.002	0.001
CORE	0.153	0.051	0.028	0.011	0.005	0.001	0.000
HierG2G	0.126	0.056	0.027	0.012	0.006	0.003	0.001
HierG2G+BT	0.127	0.046	0.023	0.005	0.003	0.001	0.000
JTVAE	0.032	0.022	0.013	0.010	0.005	0.003	0.002
UGMMT	0.179	0.104	0.054	0.025	0.010	0.006	0.003

* Success rate : the ratio of valid and novel SMILES strings satisfying both conditions *Improvement* ≥ *0.05* and *Similarity* ≥ *threshold*

Table S3. Molecular generative performance to SOTA models on DRD2 benchmark test

	TOTAL SCORE	VALID RATIO	PROPERTY	IMPROVE-MENT	SIMILARITY	NOVELTY	DIVERSITY
COMA	**0.7142**	1.000	0.799	0.792	0.329	0.999	0.366
VJTNN	0.6787	0.999	0.803	0.797	0.344	0.696	0.433
VJTNN+GAN	0.7137	0.999	0.787	0.780	0.324	0.885	0.507
CORE	0.6947	0.999	0.800	0.793	0.338	0.779	0.459
HierG2G	0.6922	0.981	0.680	0.673	0.258	0.971	0.590
HierG2G+BT	0.6783	1.000	0.808	0.802	0.336	0.671	0.453
JTVAE	0.4077	0.935	0.091	0.084	0.402	0.934	0.000
UGMMT	0.7088	0.999	0.737	0.730	0.261	0.996	0.530

* Total score : the mean of the below six metrics
* Validity : the ratio of valid SMILES strings generated from test data
* Property : the average of property scores of valid SMILES strings generated from test data
* Improvement : the average of difference of property scores between valid target SMILES strings and source SMILES string of test data
* Similarity : the average of the *Tanimoto similarity* between valid target SMILES strings and source SMILES string of test data
* Novelty : the ratio of valid SMILES strings that are not in training data
* Diversity : the average of *Tanimoto dissimilarity (=1 - Tanimoto similarity)* between valid target SMILES strings

Table S4. Success rates of similarity-constrained molecular optimization on QED benchmark test

	Threshold of similarity						
	**0.40**	**0.45**	**0.50**	**0.55**	**0.60**	**0.65**	**0.70**
COMA	0.614	0.493	0.394	**0.284**	**0.181**	**0.095**	**0.044**
VJTNN	0.641	0.501	0.368	0.241	0.126	0.069	0.029
VJTNN+GAN	**0.669**	0.514	0.376	0.244	0.141	0.065	0.024
CORE	0.653	**0.525**	**0.399**	0.258	0.150	0.076	0.035
HierG2G	0.573	0.431	0.293	0.171	0.091	0.044	0.023
HierG2G+BT	0.505	0.289	0.172	0.085	0.034	0.013	0.005
JTVAE	0.133	0.105	0.073	0.051	0.033	0.024	0.013
UGMMT	0.384	0.280	0.205	0.136	0.090	0.048	0.024

* Success rate : the ratio of valid and novel SMILES strings satisfying both conditions *Improvement* $\geq$ *0.1* and *Similarity* $\geq$ *threshold*

Table S5. Molecular generative performance to SOTA models on QED benchmark test

	TOTAL SCORE	VALID RATIO	PROPERTY	IMPROVE-MENT	SIMILARITY	NOVELTY	DIVERSITY
COMA	0.6505	1.000	0.866	0.114	0.343	1.000	0.580
VJTNN	0.6728	1.000	0.904	0.151	0.308	1.000	0.674
VJTNN+GAN	0.6730	1.000	0.898	0.145	0.312	1.000	0.683
CORE	0.6733	1.000	0.899	0.146	0.317	1.000	0.678
HierG2G	0.6462	0.961	0.859	0.135	0.271	0.961	0.690
HierG2G+BT	**0.6788**	1.000	0.906	0.153	0.241	1.000	0.773
JTVAE	0.5037	0.926	0.734	0.037	0.400	0.925	0.000
UGMMT	0.6290	0.998	0.851	0.100	0.332	0.998	0.495

* Total score : the mean of the below six metrics
* Validity : the ratio of valid SMILES strings generated from test data
* Property : the average of property scores of valid SMILES strings generated from test data
* Improvement : the average of difference of property scores between valid target SMILES strings and source SMILES string of test data
* Similarity : the average of the *Tanimoto similarity* between valid target SMILES strings and source SMILES string of test data
* Novelty : the ratio of valid SMILES strings that are not in training data
* Diversity : the average of *Tanimoto dissimilarity (=1 - Tanimoto similarity)* between valid target SMILES strings

Table S6. Success rates of similarity-constrained molecular optimization on penalized LogP04 benchmark test

	Threshold of similarity						
	**0.40**	**0.45**	**0.50**	**0.55**	**0.60**	**0.65**	**0.70**
COMA	**0.406**	**0.289**	**0.181**	**0.108**	**0.063**	**0.025**	0.004
VJTNN	0.359	0.231	0.146	0.058	0.021	0.011	**0.005**
VJTNN+GAN	0.388	0.250	0.150	0.068	0.034	0.010	0.000
CORE	0.368	0.246	0.158	0.069	0.030	0.013	**0.005**
HierG2G	0.310	0.183	0.099	0.030	0.010	0.001	0.000
HierG2G+BT	0.275	0.160	0.079	0.020	0.004	0.001	0.000

* Success rate : the ratio of valid and novel SMILES strings satisfying both conditions *Improvement* $\geq$ *4.0* and *Similarity* $\geq$ *threshold*

Table S7. Molecular generative performance to SOTA models on penalized LogP04 benchmark test

	TOTAL SCORE	VALID RATIO	PROPERTY	IMPROVE-MENT	SIMILARITY	NOVELTY	DIVERSITY
COMA	**1.5883**	0.995	1.945	4.663	0.308	0.995	0.624
VJTNN	1.4113	0.975	1.489	4.095	0.316	0.975	0.618
VJTNN+GAN	1.4497	0.990	1.543	4.242	0.326	0.990	0.607
CORE	1.4063	0.984	1.431	4.087	0.324	0.984	0.628
HierG2G	1.3952	0.948	1.510	4.052	0.266	0.948	0.647
HierG2G+BT	1.3735	0.981	1.335	3.983	0.269	0.981	0.692

* Total score : the mean of the below six metrics
* Validity : the ratio of valid SMILES strings generated from test data
* Property : the average of property scores of valid SMILES strings generated from test data
* Improvement : the average of difference of property scores between valid target SMILES strings and source SMILES string of test data
* Similarity : the average of the *Tanimoto similarity* between valid target SMILES strings and source SMILES string of test data
* Novelty : the ratio of valid SMILES strings that are not in training data
* Diversity : the average of *Tanimoto dissimilarity (=1 - Tanimoto similarity)* between valid target SMILES strings

Table S8. Success rates of similarity-constrained molecular optimization on penalized LogP06 benchmark test

	Threshold of similarity						
	**0.40**	**0.45**	**0.50**	**0.55**	**0.60**	**0.65**	**0.70**
COMA	**0.454**	**0.368**	**0.288**	**0.195**	**0.110**	**0.056**	**0.018**
VJTNN	0.196	0.148	0.101	0.060	0.034	0.015	0.003
VJTNN+GAN	0.298	0.224	0.155	0.095	0.049	0.018	0.005
CORE	0.259	0.189	0.141	0.079	0.058	0.019	0.005
HierG2G	0.159	0.115	0.076	0.039	0.019	0.009	0.001
HierG2G+BT	0.230	0.174	0.106	0.055	0.021	0.006	0.004

* Success rate : the ratio of valid and novel SMILES strings satisfying both conditions *Improvement* ≥ *4.0* and *Similarity* ≥ *threshold*

Table S9. Molecular generative performance to SOTA models on penalized LogP06 benchmark test

	TOTAL SCORE	VALID RATIO	PROPERTY	IMPROVE-MENT	SIMILARITY	NOVELTY	DIVERSITY
COMA	**1.3285**	0.958	1.313	3.860	0.383	0.958	0.499
VJTNN	0.9812	0.826	0.768	2.772	0.399	0.825	0.297
VJTNN+GAN	1.1280	0.881	0.987	3.222	0.397	0.881	0.400
CORE	1.0617	0.859	0.871	3.037	0.396	0.859	0.348
HierG2G	0.9943	0.825	0.788	2.828	0.344	0.825	0.356
HierG2G+BT	1.0285	0.849	0.830	2.908	0.374	0.849	0.361

* Total score : the mean of the below six metrics
* Validity : the ratio of valid SMILES strings generated from test data
* Property : the average of property scores of valid SMILES strings generated from test data
* Improvement : the average of difference of property scores between valid target SMILES strings and source SMILES string of test data
* Similarity : the average of the *Tanimoto similarity* between valid target SMILES strings and source SMILES string of test data
* Novelty : the ratio of valid SMILES strings that are not in training data
* Diversity : the average of *Tanimoto dissimilarity (=1 - Tanimoto similarity)* between valid target SMILES strings

Table S10. List of 20 SMILES strings for principal component analysis in DRD2

Label	SMILES
S1	CN1C(CN2CCN(C3=NC=CC=N3)CC2)=CC(=O)N(C)C1=O
S2	CC(SC1=NC=C(C2=CC=CC=C2)N1C)C(=O)NC1CCCC2=CC=CC=C21
S3	O=C(NC1=CN=C2C=CC=CC2=C1)C1=CC=CC(S(=O)(=O)N2CCN(C3=CC=CC=C3)CC2)=C1
S4	NC(=O)N1CCN(C(=O)C2=CC=CC=C2)CC1
S5	O=C(CN1CCCCC1)NC(=O)NCC1=CC=CC=C1
S6	O=S(=O)(CC1=CC=CC=C1Cl)NCC1CN2CCCCC2CO1
S7	CC(NC(=O)CN1CCCC1)C1=CC=CC=C1
S8	CC1=NC([N+](=O)[O-])=CN1CC(=O)NCC1CN2CCCCC2CO1
S9	CN1C(=O)N(CC(=O)N2CCN(CC3=CC=CC=C3)CC2)C2=CC=CC=C21
S10	CC1=CC=C(S(=O)(=O)NCC2CN3CCCCC3CO2)C=C1
T1	CC1(C)CC(=O)N(CCCCN2CCN(C3=NC=CC=N3)CC2)C(=O)C1
T2	CC(C(=O)NC1CCCC2=CC=CC=C21)N1CCN(C2=CC=C(F)C=C2)CC1
T3	O=S(=O)(C1=CN=C2C=CC=CC2=C1)N1CCC(CCN2CCN(C3=CC=C(Cl)C=C3)CC2)CC1
T4	O=C(C1=CC=NC=C1)N1CCN(C2CCC(C3=CC=CC=C3)CC2)CC1
T5	O=C(CN1CCN(CC2=CC=CC=C2)CC1)NC1=CC=CC=C1C1=CC=CC=C1
T6	C1=CC=C2C(=C1)CCCC2NCC1CN2CCCC2CO1
T7	O=C(CN1CCN(C2=CC=CC=C2F)CC1)NC(C1=CC=CC=C1)C1=CC=CC=C1
T8	O=C(NCC1CN2CCCCC2CO1)NC1CCC2=CC(F)=CC=C21
T9	OC1=CC=C(N2CCN(CC3=CC=CC=C3)CC2)C=C1
T10	CNC1=CC(C(=O)NCC2CN3CCCC3CO2)=CC=N1

Table S11. Information of targeted proteins in use-case

Protein Name	Uniprot ID	PDB ID	FASTA
ABCG2	Q9UNQ0	6VXH	MSSSNVEVFIPVSQGNTNGFPATASNDLKAFTEGAVLSFHN ICYRVKLKSGFLPCRKPVEKEILSNINGIMKPGLNAILGPTG GGKSSLLDVLAARKDPSGLSGDVLINGAPRPANFKCNSGY VVQDDVVMGTLTVRENLQFSAALRLATTMTNHEKNERIN RVIQELGLDKVADSKVGTQFIRGVSGGERKRTSIGMELITD PSILFLDEPTTGLDSSTANAVLLLLKRMSKQGRTIIFSIHQPR YSIFKLFDSLTLLASGRLMFHGPAQEALGYFESAGYHCEAY NNPADFFLDIINGDSTAVALNREEDFKATEIIEPSKQDKPLIE KLAEIYVNSSFYKETKAELHQLSGGEKKKKITVFKEISYTTS FCHQLRWVSKRSFKNLLGNPQASIAQIIVTVVLGLVIGAIYF GLKNDSTGIQNRAGVLFFLTTNQCFSSVSAVELFVVEKKLF IHEYISGYYRVSSYFLGKLLSDLLPMRMLPSIIFTCIVYFMLG LKPKADAFFVMMFTLMMVAYSASSMALAIAAGQSVVSVA TLLMTICFVFMMIFSGLLVNLTTIASWLSWLQYFSIPRYGFT ALQHNEFLGQNFCPGLNATGNNPCNYATCTGEEYLVKQGI DLSPWGLWKNHVALACMIVIFLTIAYLKLLFLKKYS
BRAF	P15056	1UWH	MAALSGGGGGGAEPGQALFNGDMEPEAGAGAGAAASSA ADPAIPEEVWNIKQMIKLTQEHIEALLDKFGGEHNPPSIYLE AYEEYTSKLDALQQREQQLLESLGNGTDFSVSSSASMDTV TSSSSSSLSVLPSSLSVFQNPTDVARSNPKSPQKPIVRVFLPN KQRTVVPARCGVTVRDSLKKALMMRGLIPECCAVYRIQD GEKKPIGWDTDISWLTGEELHVEVLENVPLTTHNFVRKTFF TLAFCDFCRKLLFQGFRCQTCGYKFHQRCSTEVPLMCVNY DQLDLLFVSKFFEHHPIPQEEASLAETALTSGSSPSAPASDSI GPQILTSPSPSKSIPIPQPFRPADEDHRNQFGQRDRSSSAPNV HINTIEPVNIDDLIRDQGFRGDGGSTTGLSATPPASLPGSLTN VKALQKSPGPQRERKSSSSSEDRNRMKTLGRRDSSDDWEIP DGQITVGQRIGSGSFGTVYKGKWHGDVAVKMLNVTAPTP QQLQAFKNEVGVLRKTRHVNILLFMGYSTKPQLAIVTQWC EGSSLYHHLHIIETKFEMIKLIDIARQTAQGMDYLHAKSIIH RDLKSNNIFLHEDLTVKIGDFGLATVKSRWSGSHQFEQLSG SILWMAPEVIRMQDKNPYSFQSDVYAFGIVLYELMTGQLP YSNINNRDQIIFMVGRGYLSPDLSKVRSNCPKAMKRLMAE CLKKKRDERPLFPQILASIELLARSLPKIHRSASEPSLNRAGF QTEDFSLYACASPKTPIQAGGYGAFPVH

Table S12. List of candidate molecules translated from sorafenib by COMA

Name	SMILES	Tanimoto similarity	Binding Affinity	
			BRAF	ABCG2
Sorafenib	CNC(=O)C1=CC(OC2=CC=C(NC(=O)NC3=CC=C(Cl)C(C(F)(F)F)=C3)C=C2)=CC=N1	1.000	6.235	4.989
COMA001	CNC(=O)C1=CC(NC(=O)C2=CC=C(OC(F)(F)F)C=C2)=CC=N1	0.476	6.200	4.089
COMA002	CNC(=O)C1=CC(NC(=O)C2=CC=C(OC(F)(F)F)C(F)=C2)=CC=N1	0.406	6.362	4.099
COMA003	CNC(=O)C1=CC(NC(=O)C2=CC=CC(OC(F)(F)F)=C2)=CC=N1	0.433	6.345	4.159
COMA004	CNC(=O)C1=CC(C2=CC=C(NC(=O)C3=CC=CC=C3)C(C(F)(F)F)=C2)=CC=N1	0.412	6.144	4.212
COMA005	CNC(=O)C1=CC(C2=CC=C(NC(=O)C3=CC=C(OC(F)(F)F)C=C3)C=C2)=CC=N1	0.470	6.456	4.238
COMA006	CNC(=O)C1=CC(C2=CC=C(NC(=O)C3=CC=CC=C3)C=C2C(F)(F)F)=CC=N1	0.485	6.200	4.331
COMA007	CNC(=O)C1=CC(C2=CC=C(NC(=O)C3=CC=CC=C3C(F)(F)F)C=C2)=CC=N1	0.477	5.846	4.339
COMA008	CNC(=O)C1=CC(NC(=O)C2=CC=C(OC(F)(F)F)C(Cl)=C2)=CC=N1	0.463	6.232	4.347
COMA009	CNC(=O)C1=CC(C2=CC=C(NC(=O)C3=CC=CC(OC(F)(F)F)=C3)C=C2)=CC=N1	0.429	6.585	4.374
COMA010	CNC(=O)C1=CC(OC2=CC=C(NC(=O)C3=CC=CC=C3)C=C2C(F)(F)F)=CC=C1NC(=O)C1=CC=CC=C1	0.403	6.437	4.416
COMA011	CNC(=O)C1=CC(C2=CC=C(OC(F)(F)F)C=C2)=CC=N1	0.443	6.117	4.436
COMA012	CNC(=O)C1=CC(SC(=O)NC2=CC=C(OC(F)(F)F)C=C2)=CC=N1	0.470	6.083	4.475
COMA013	CNC(=O)C1=CC(C2=CC=C(NC(=O)C3=CC=C(Cl)C=C3)C=C2)=CC=N1	0.422	6.170	4.567
COMA014	CNC(=O)C1=CC(C2=CC=C(NC(=O)C3=CC=CC=N3)C=C2)=CC=N1	0.419	6.249	4.583
COMA015	CNC(=O)C1=CC(OC2=CC=C(NC(=O)C3=CC=CC=C3)C=C2)=CC=C1C(F)(F)F	0.462	6.136	4.610
COMA016	CNCCNC(=O)C1=CC(OC2=CC=C(NC(=O)C3=CC=CC=C3)C=C2)=CC=N1	0.456	6.456	4.632
COMA017	CNC(=O)C1=CC(C2=CC=C(NC(=O)C3=CC=CC(Cl)=C3)C=C2)=CC=N1	0.403	6.194	4.642
COMA018	CNC(=O)C1=CC(OC2=CC=C(NC(=O)C3=CC=C(OC(F)(F)F)C=C3)C=C2)=CC=N1	0.607	6.086	4.653
COMA019	CNC(=O)C1=CC(C2=CC=C(NC(=O)C3=CC=CC(C(F)(F)F)=C3)C=C2)=CC=N1	0.426	6.298	4.671

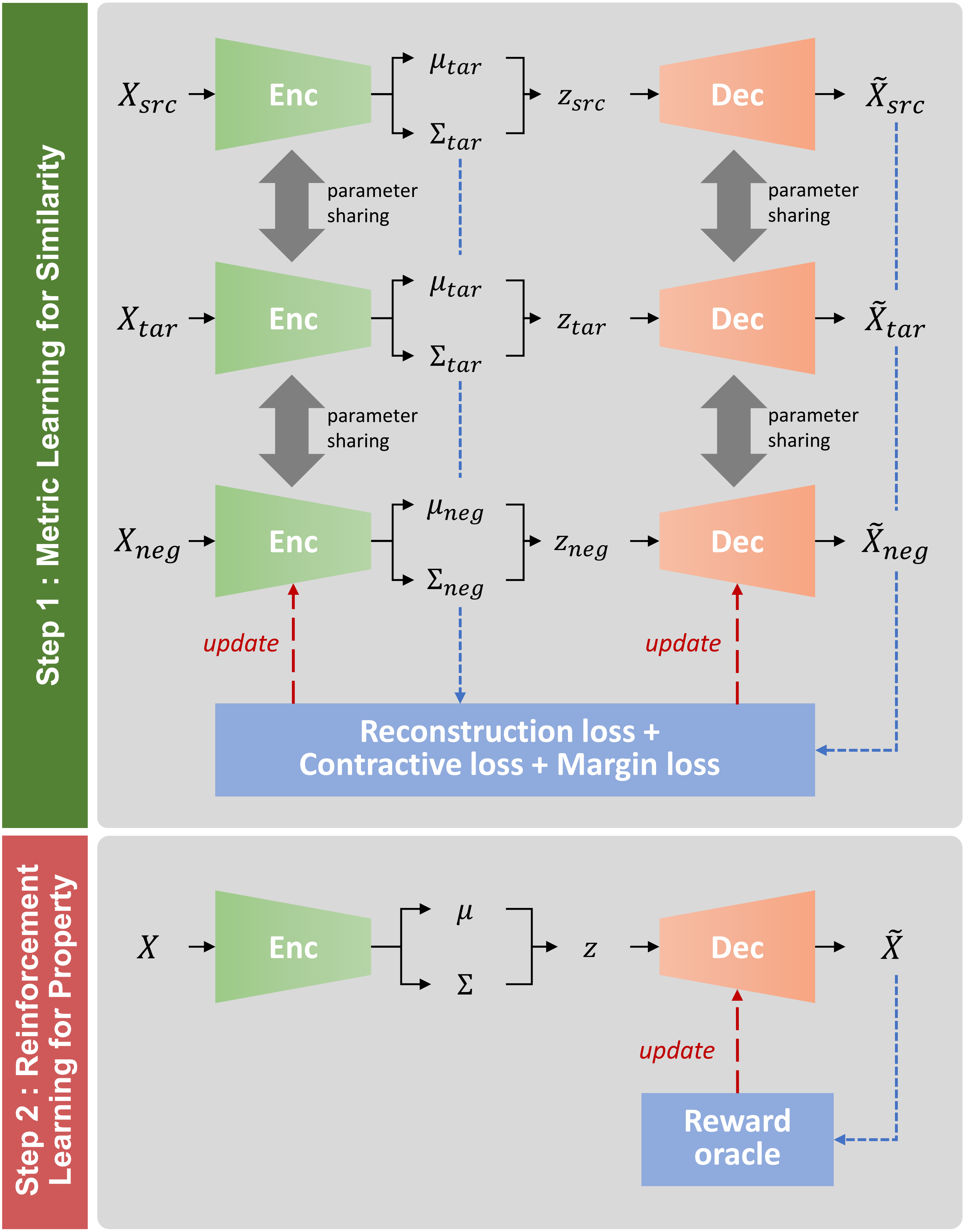

Figure S1. Overview of training procedure for COMA

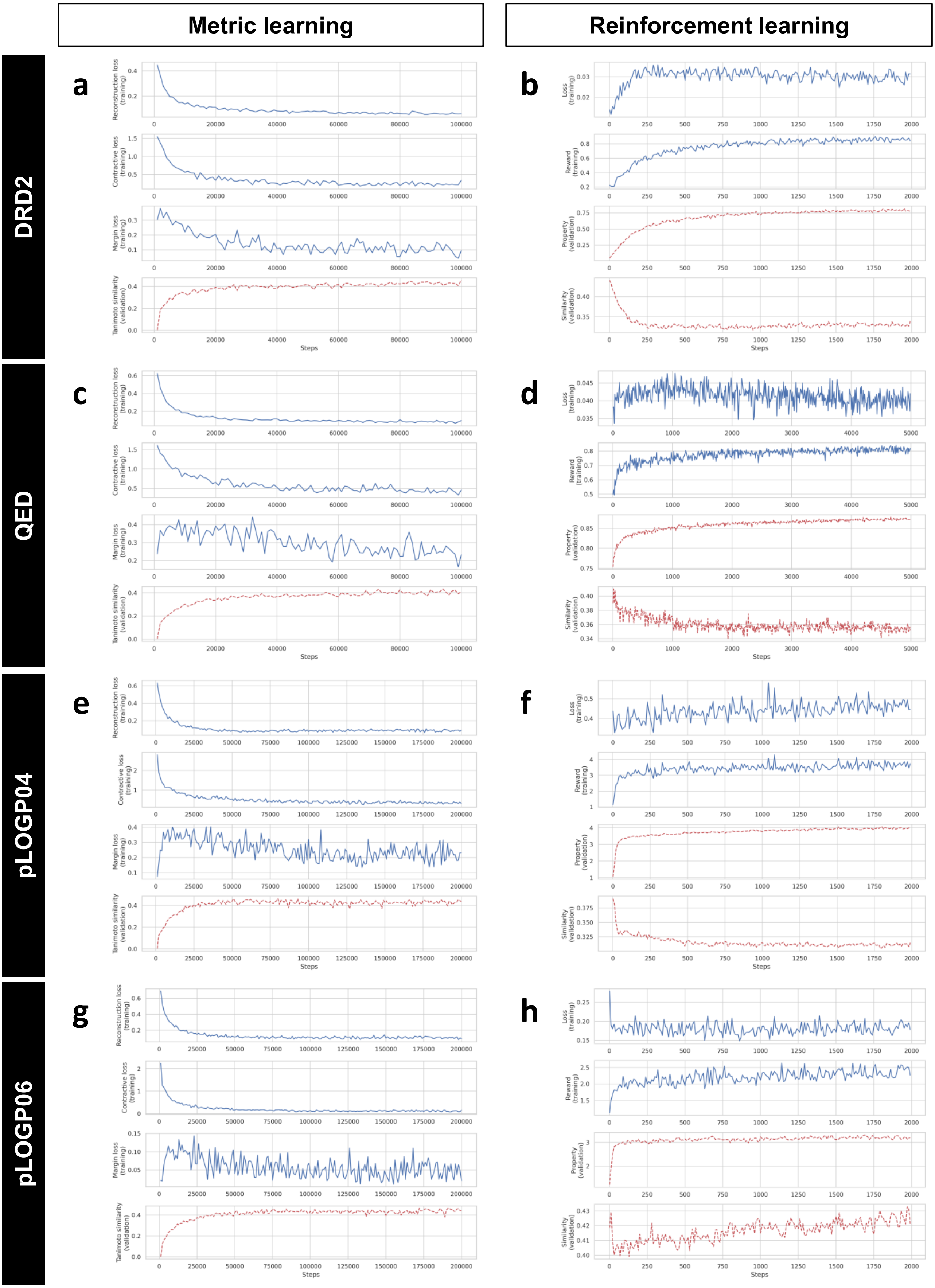

Figure S2. Loss and reward plots for COMA during pretraining and finetuning steps; **a-b**, DRD2; **c-d**, QED; **e-f**, penalized LogP04; **g-h**, penalized LogP06;

a

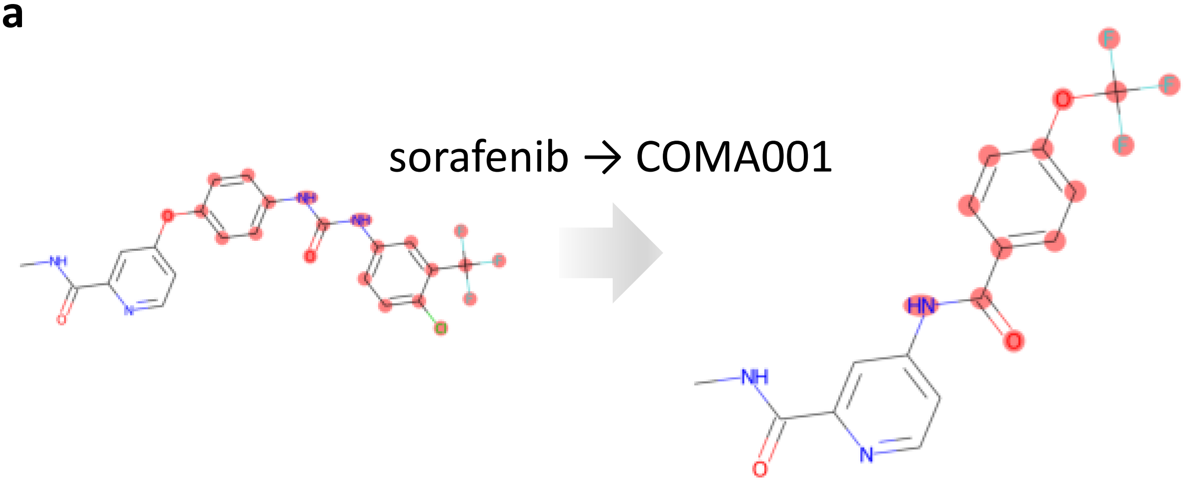

b

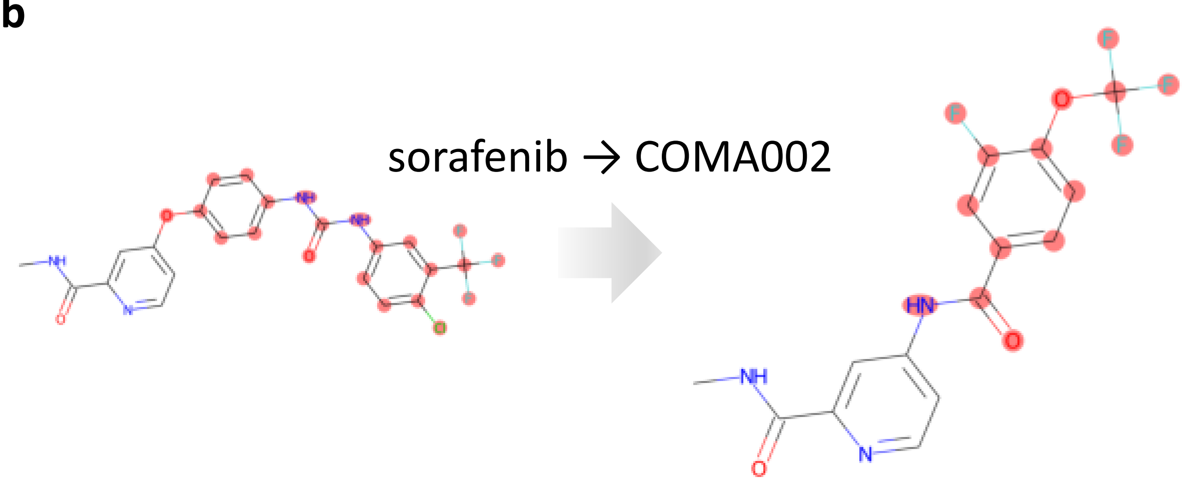

c

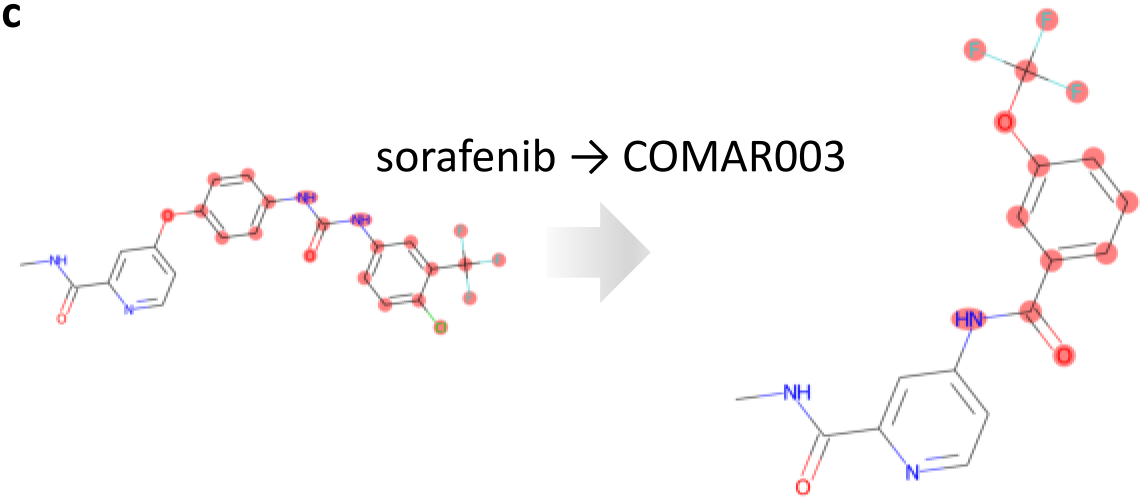

Figure S3. Comparison of molecular structures between sorafenib and candidates (cont.)

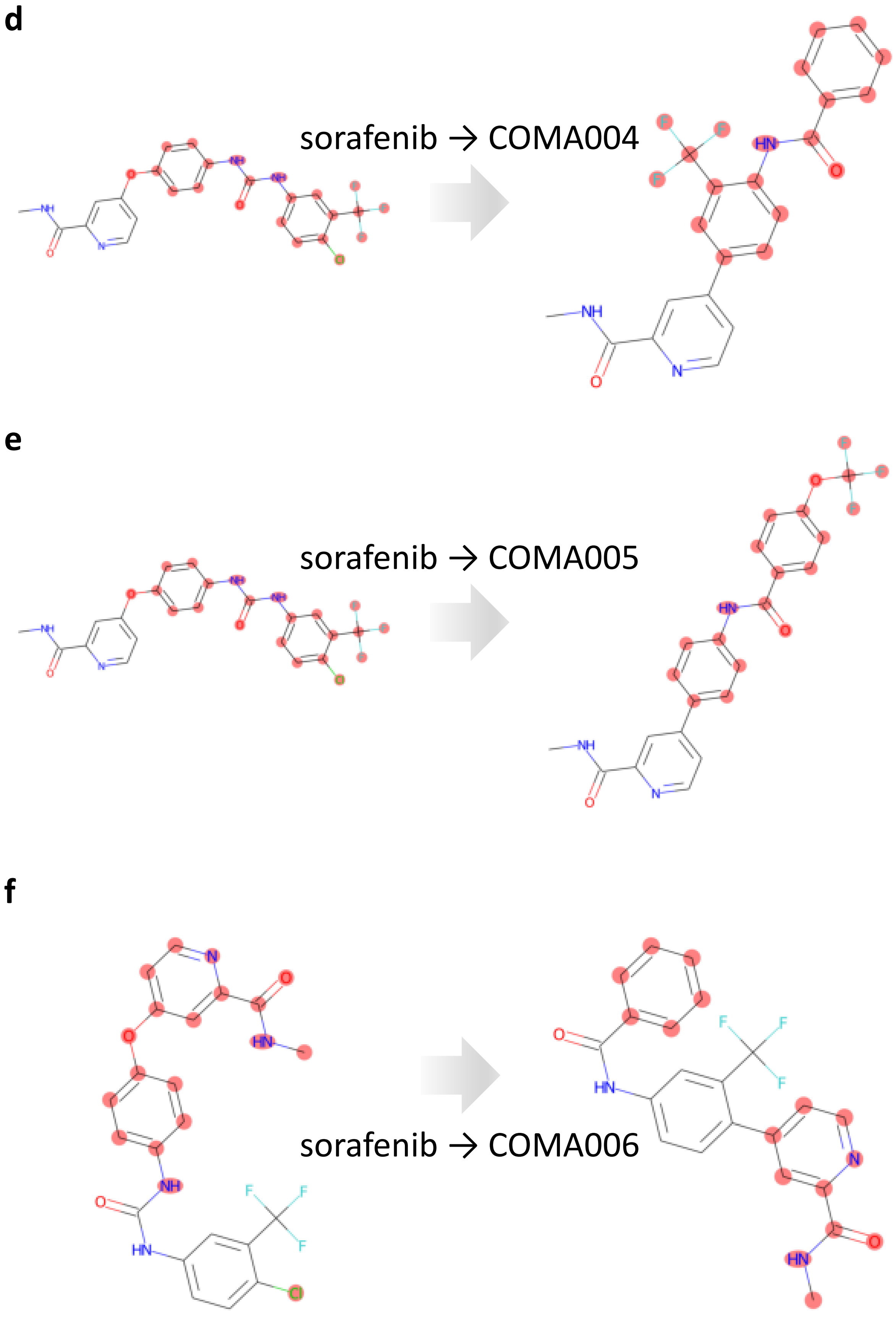

Figure S3. Comparison of molecular structures between sorafenib and candidates (cont.)

g

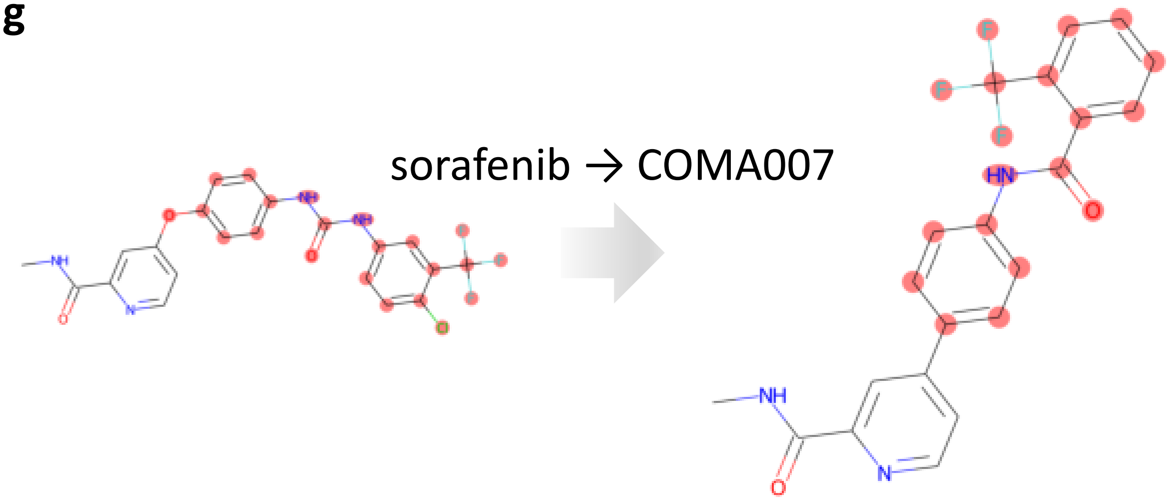

h

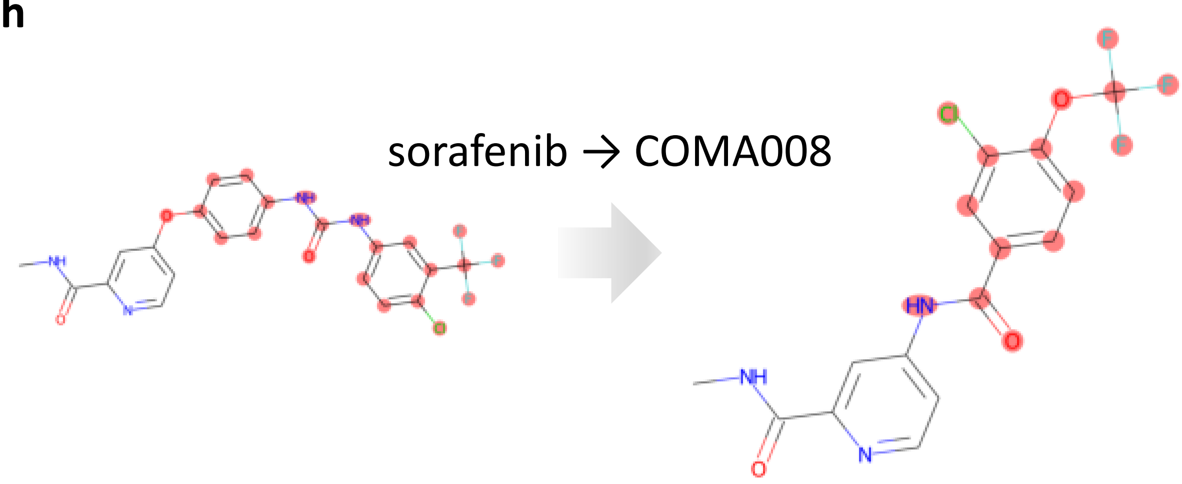

i

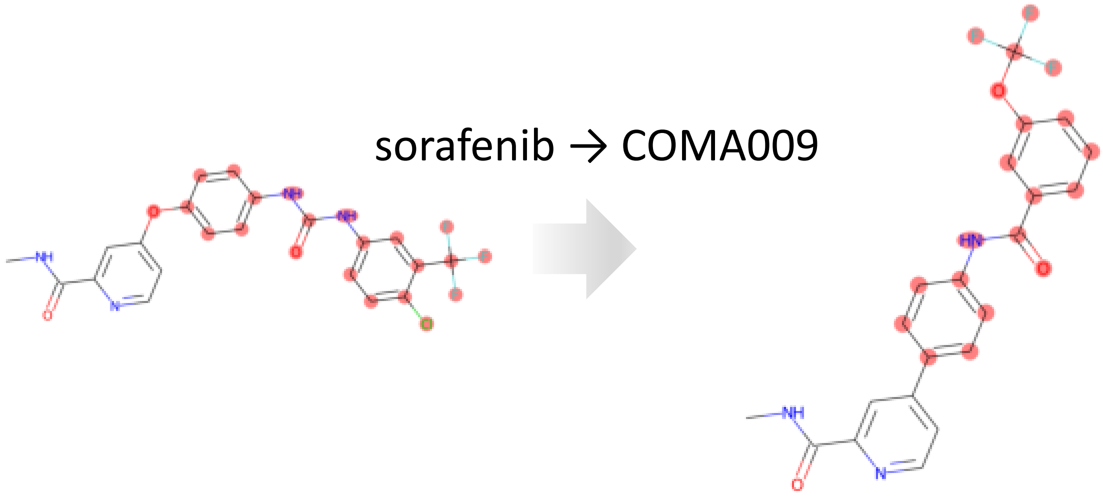

Figure S3. Comparison of molecular structures between sorafenib and candidates (cont.)

j

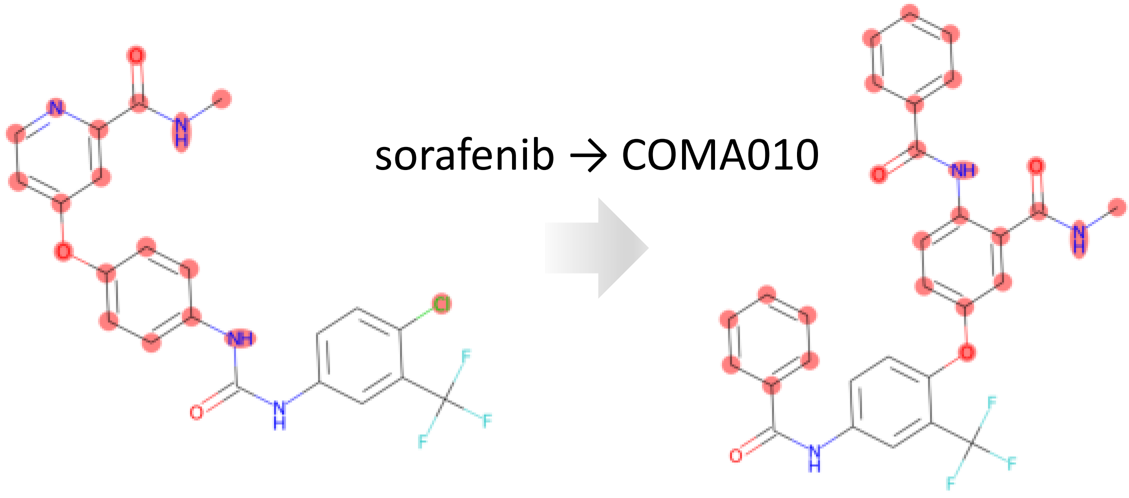

k

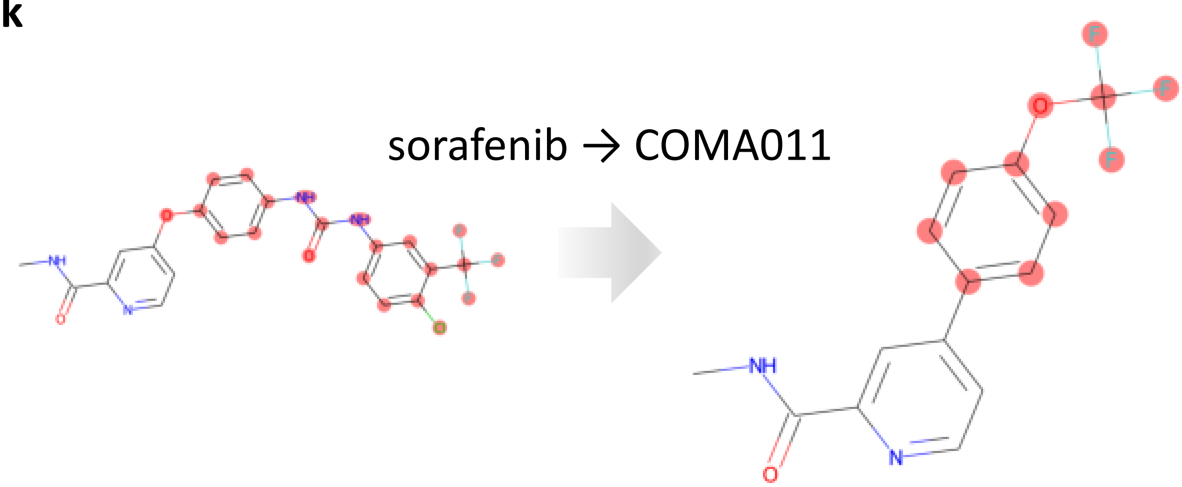

l

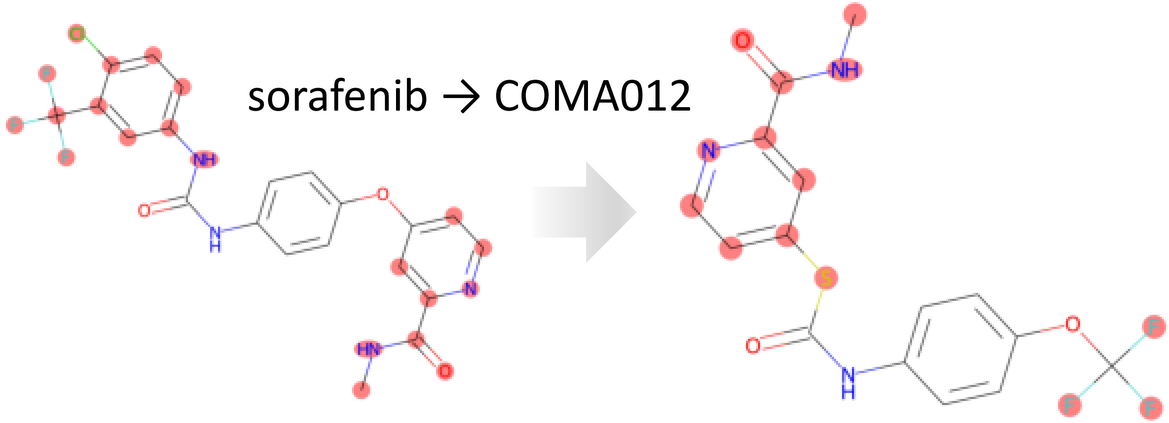

Figure S3. Comparison of molecular structures between sorafenib and candidates (cont.)

m

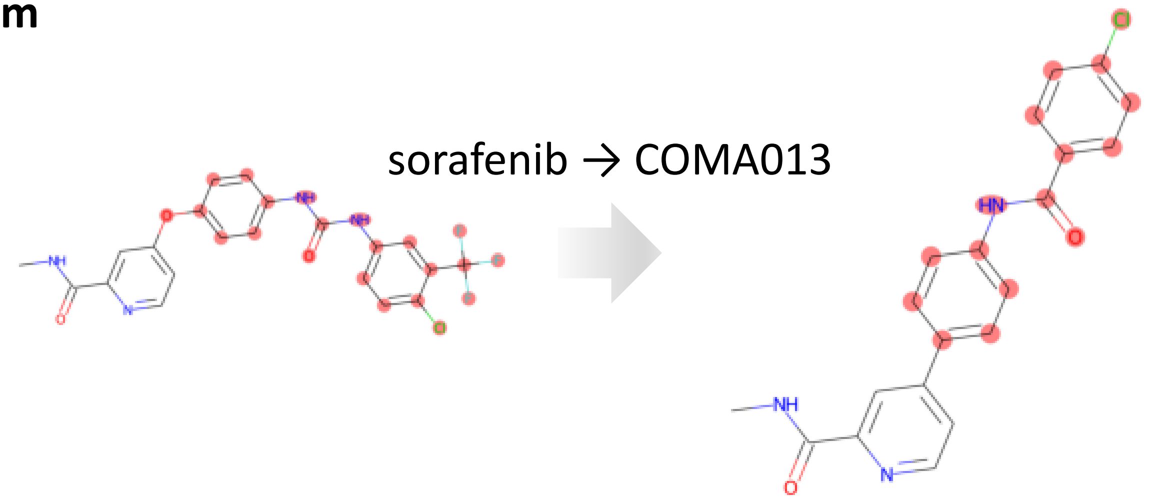

n

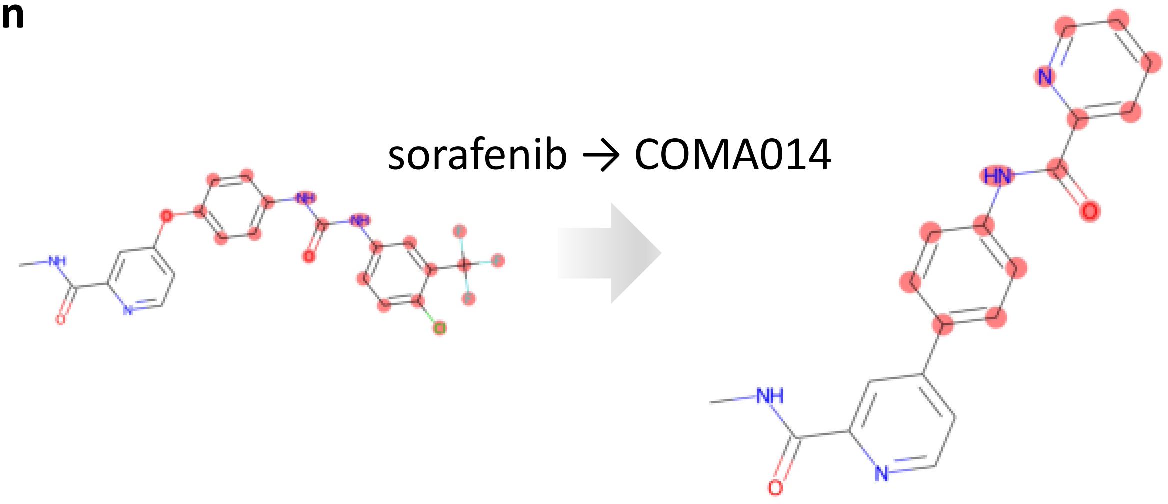

o

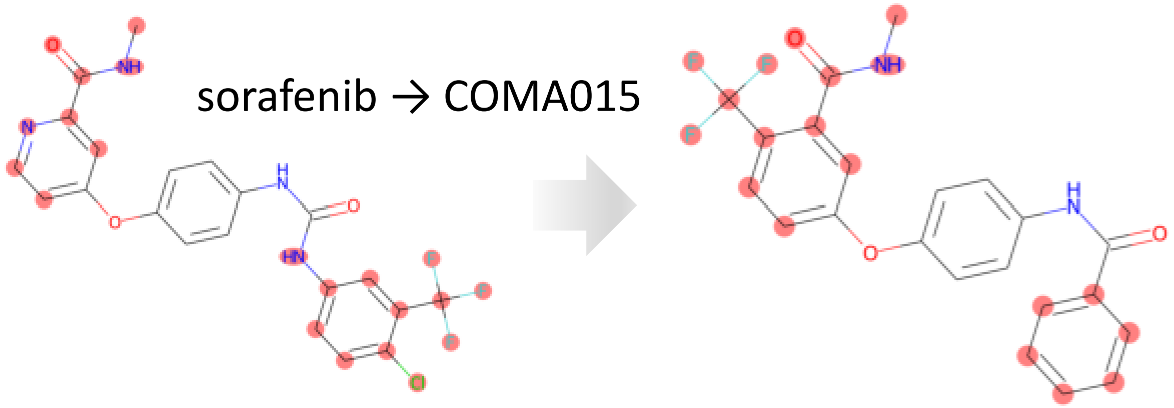

Figure S3. Comparison of molecular structures between sorafenib and candidates (cont.)

p

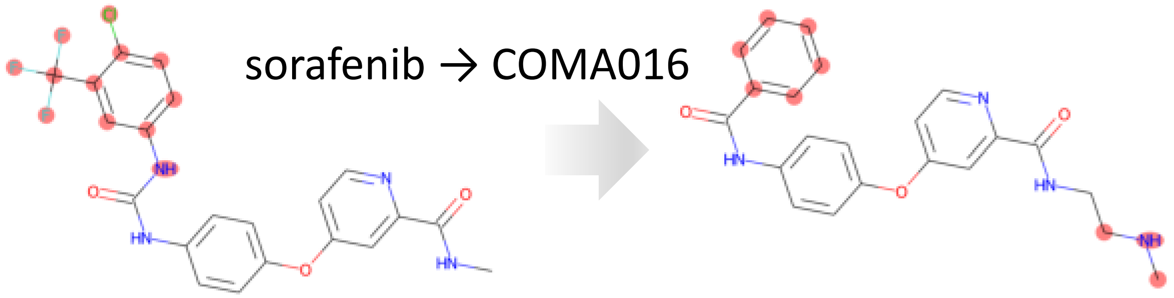

q

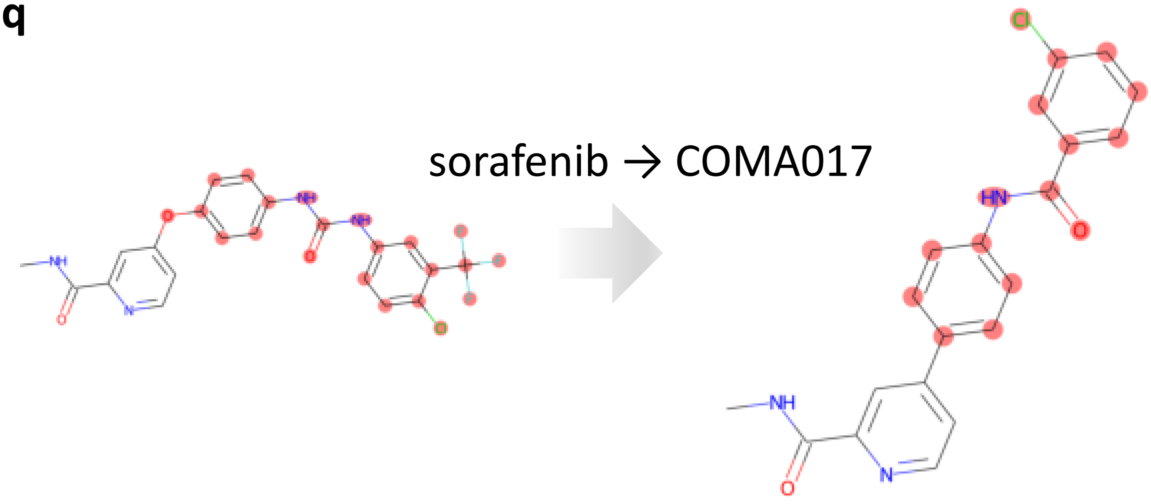

r

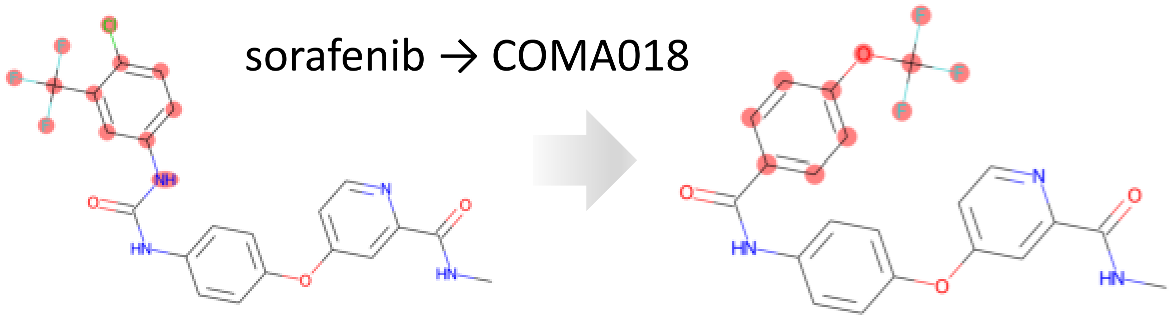

Figure S3. Comparison of molecular structures between sorafenib and candidates (cont.)

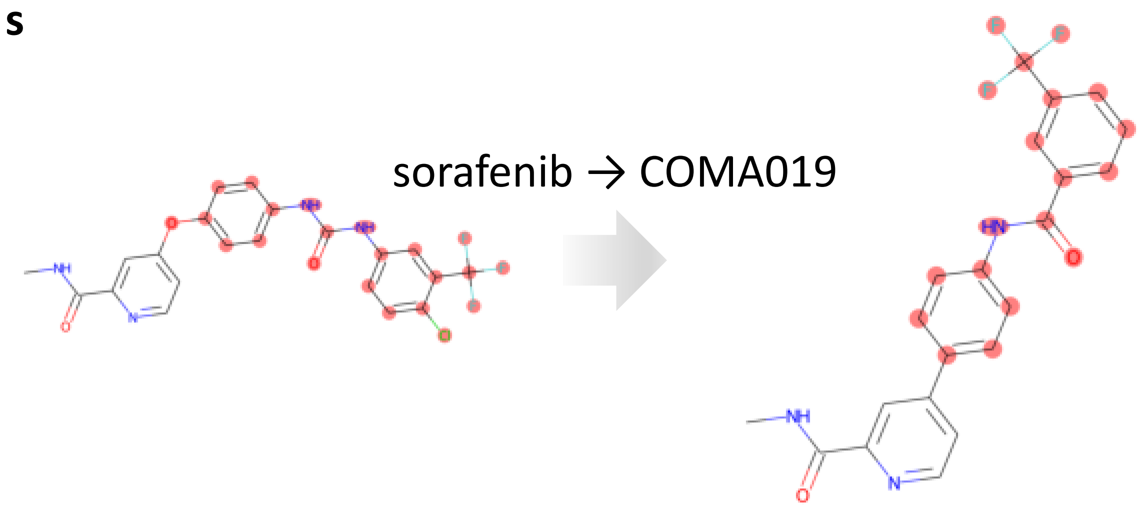

Figure S3. Comparison of molecular structures between sorafenib and candidates

a

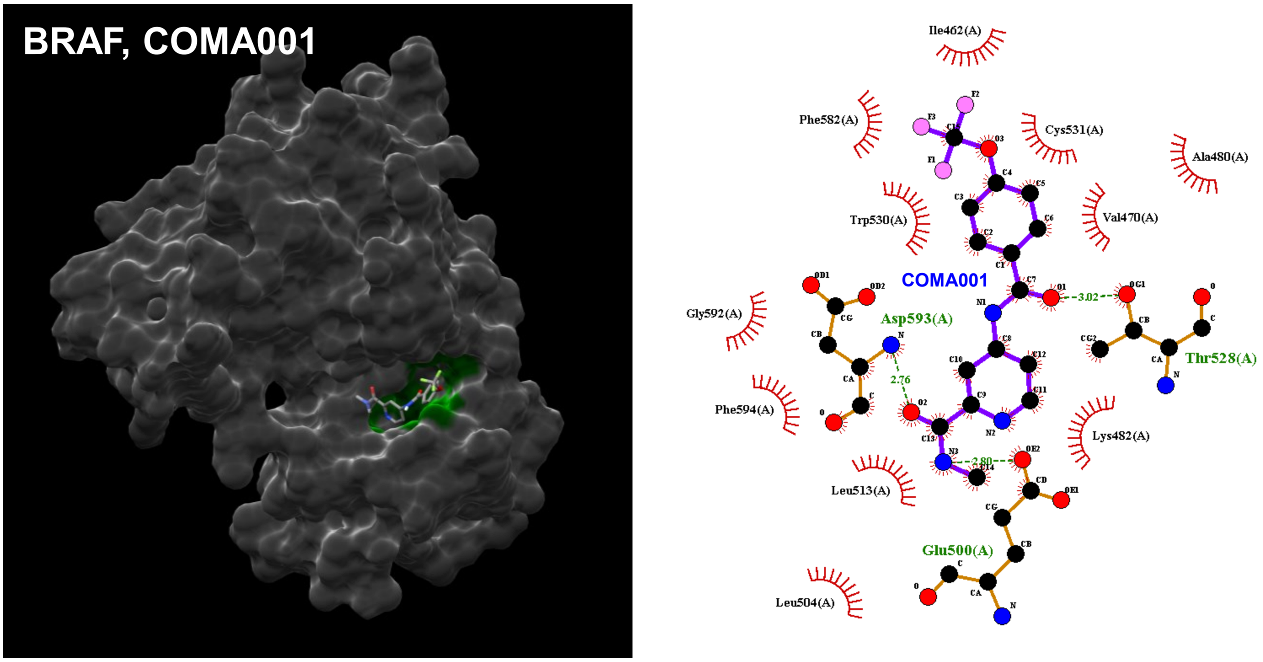

b

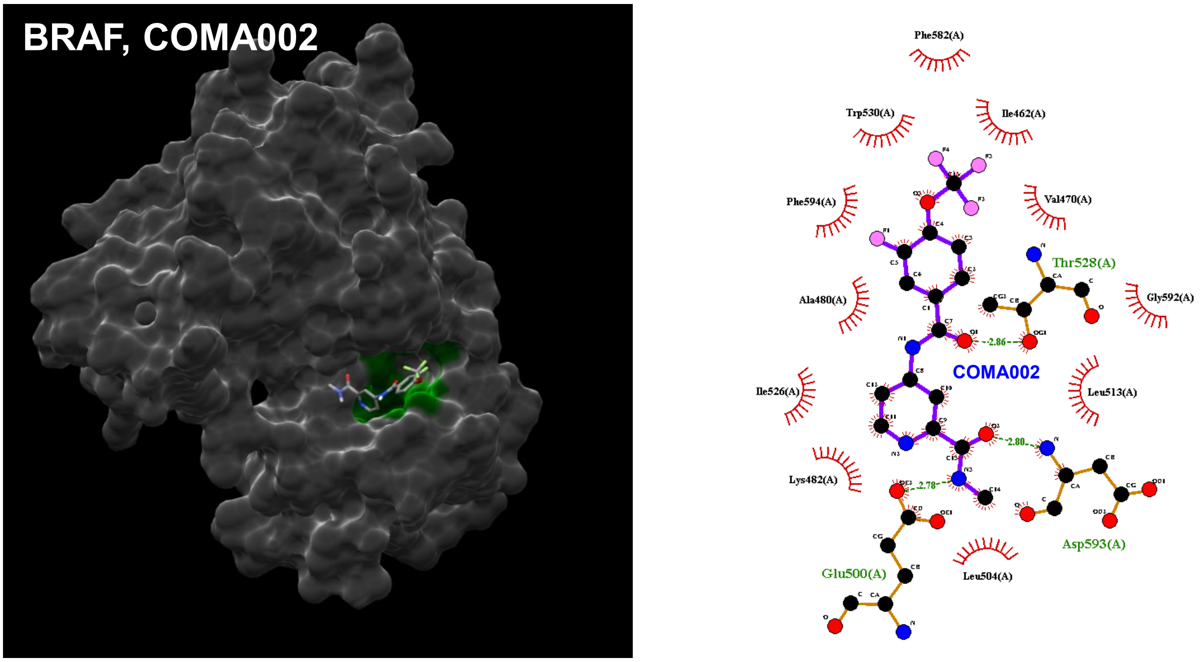

Figure S4. Docking simulation analysis of candidates. (Left) Graphics for the 3D structure of protein-ligand complex. A green surface region represents protein-ligand contacts. (Right) Diagrams of residues interacting with a ligand. Purple and yellow lines mean ligand bond and non-ligand bond, respectively. A green dashed line represents a hydrogen bond and a value on the line is its length. A red arc represents non-ligand residues involved in hydrophobic contacts. (cont.)

c

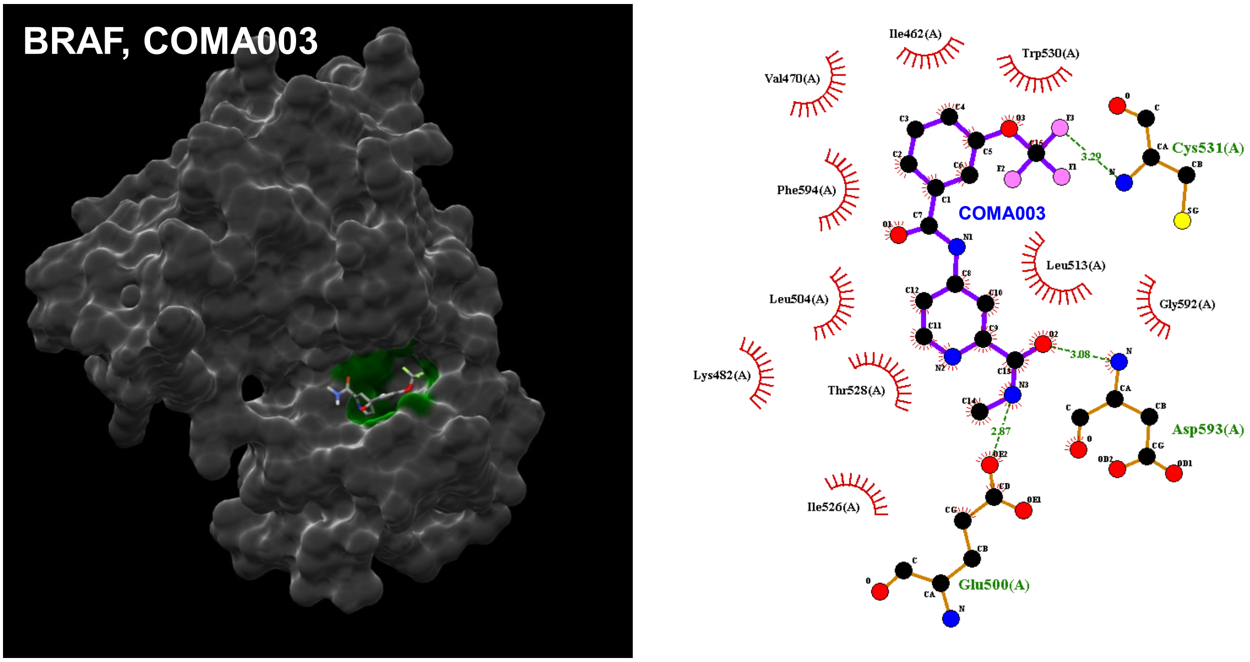

d

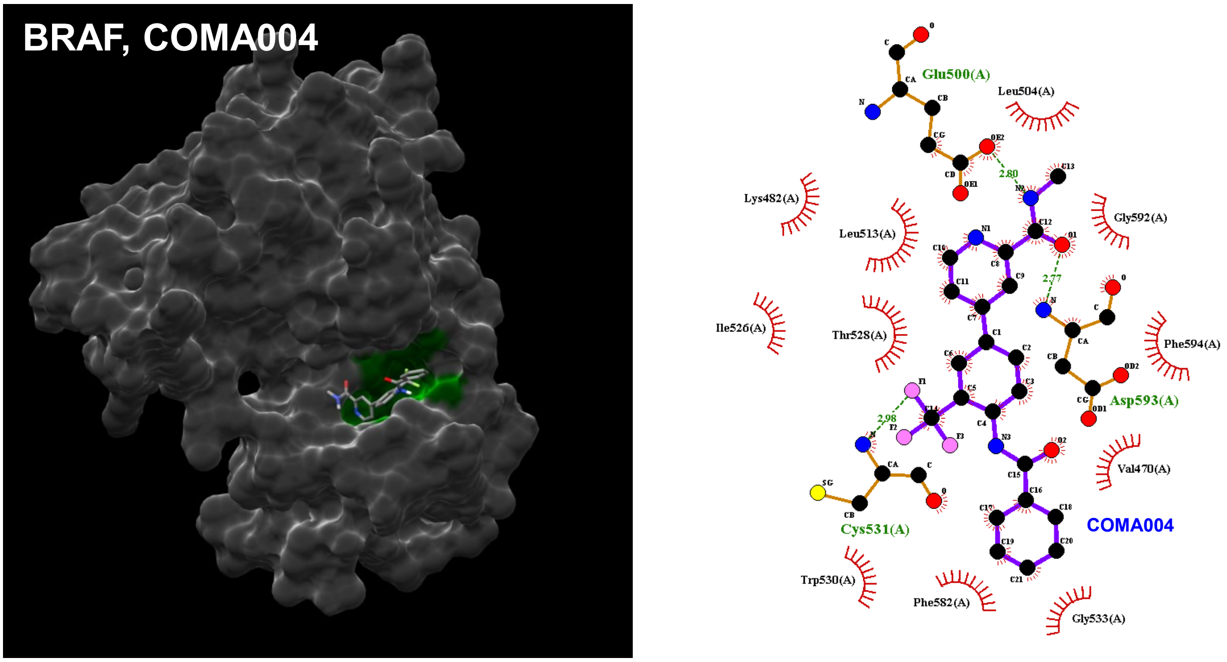

Figure S4. Docking simulation analysis of candidates. (Left) Graphics for the 3D structure of protein-ligand complex. A green surface region represents protein-ligand contacts. (Right) Diagrams of residues interacting with a ligand. Purple and yellow lines mean ligand bond and non-ligand bond, respectively. A green dashed line represents a hydrogen bond and a value on the line is its length. A red arc represents non-ligand residues involved in hydrophobic contacts. (cont.)

e

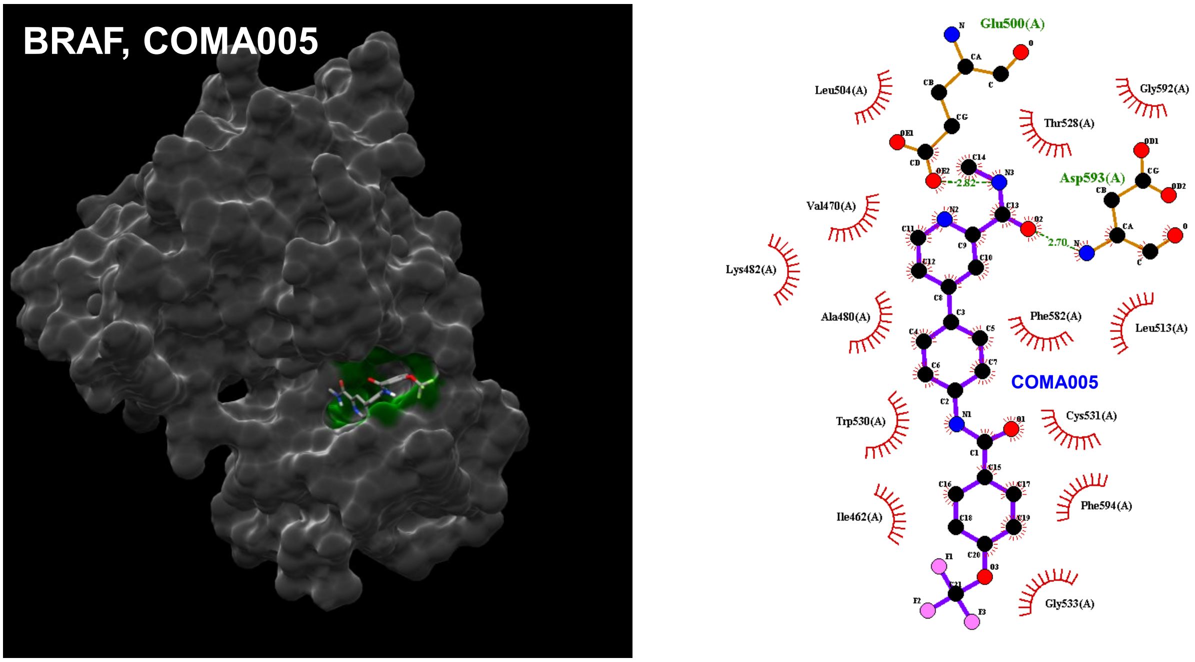

f

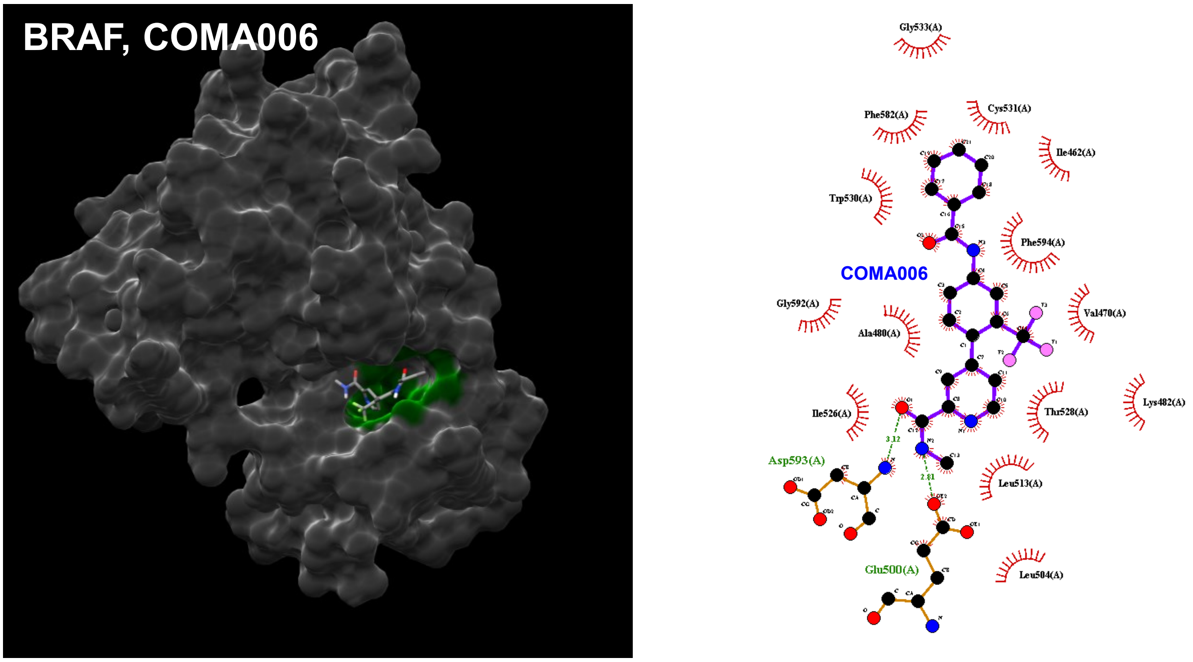

Figure S4. Docking simulation analysis of candidates. (Left) Graphics for the 3D structure of protein-ligand complex. A green surface region represents protein-ligand contacts. (Right) Diagrams of residues interacting with a ligand. Purple and yellow lines mean ligand bond and non-ligand bond, respectively. A green dashed line represents a hydrogen bond and a value on the line is its length. A red arc represents non-ligand residues involved in hydrophobic contacts. (cont.)

g

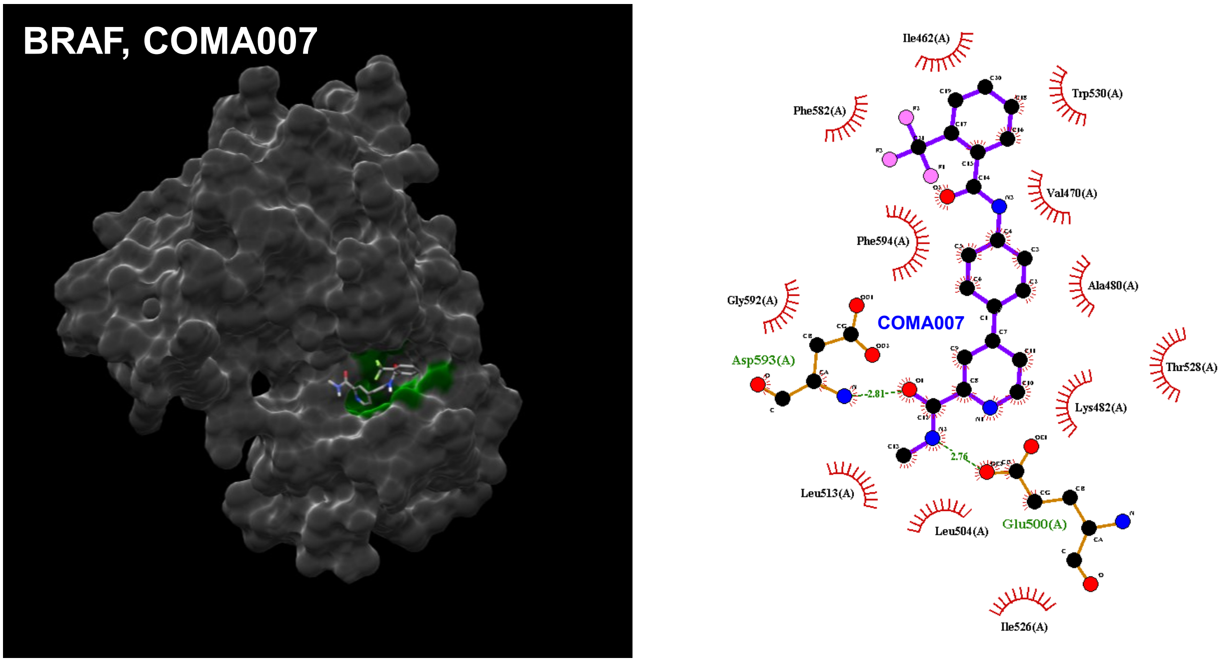

h

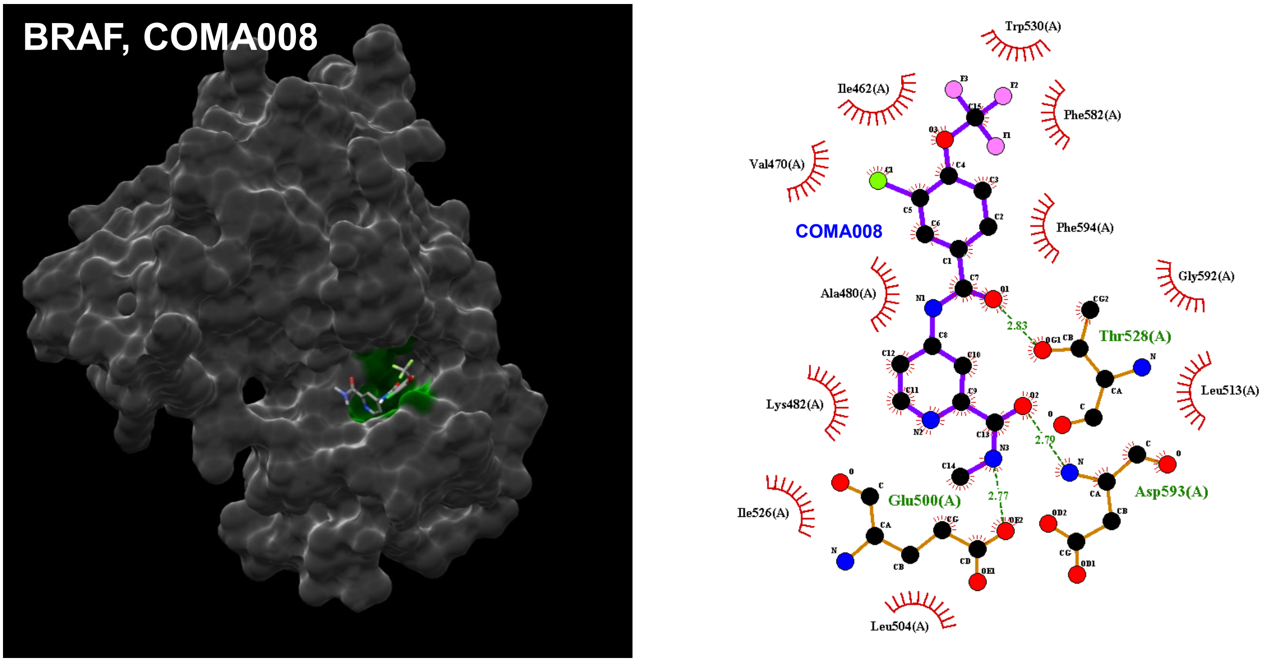

Figure S4. Docking simulation analysis of candidates. (Left) Graphics for the 3D structure of protein-ligand complex. A green surface region represents protein-ligand contacts. (Right) Diagrams of residues interacting with a ligand. Purple and yellow lines mean ligand bond and non-ligand bond, respectively. A green dashed line represents a hydrogen bond and a value on the line is its length. A red arc represents non-ligand residues involved in hydrophobic contacts. (cont.)

i

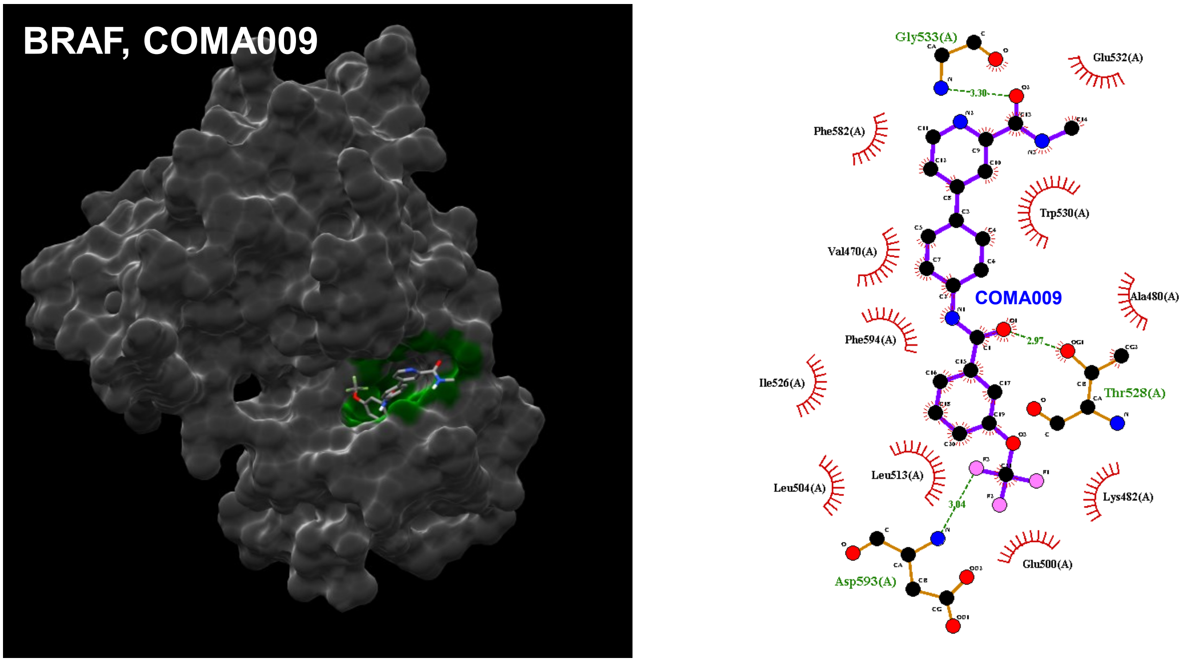

j

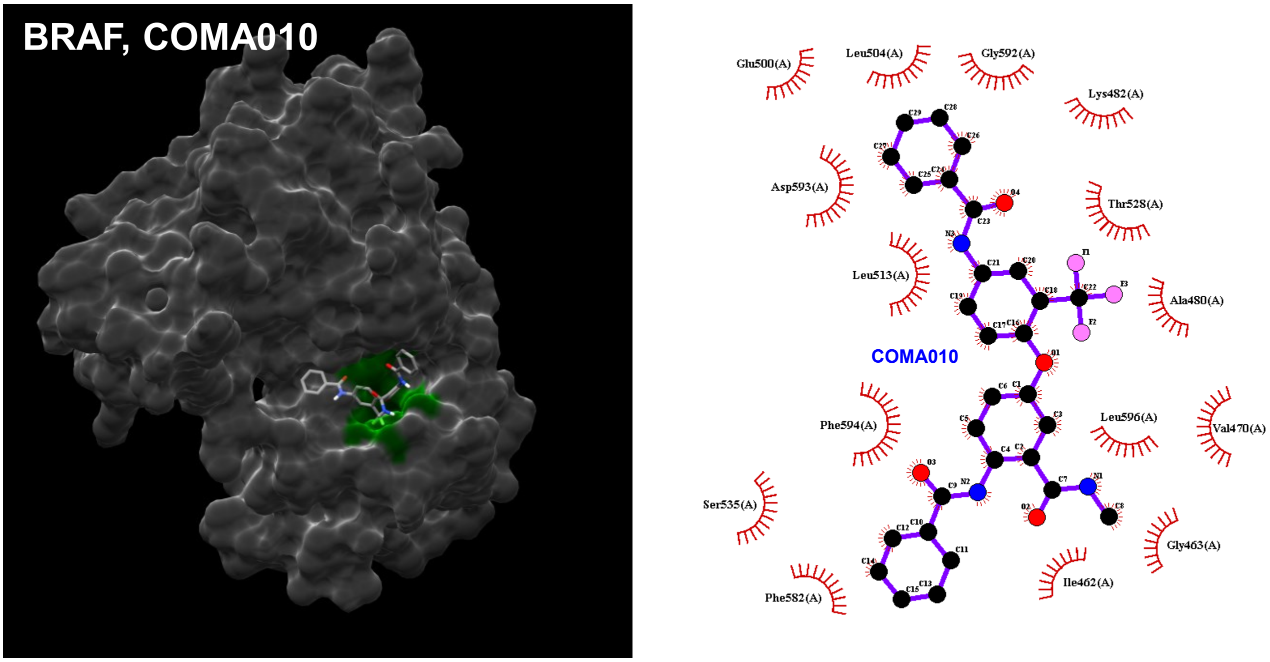

Figure S4. Docking simulation analysis of candidates. (Left) Graphics for the 3D structure of protein-ligand complex. A green surface region represents protein-ligand contacts. (Right) Diagrams of residues interacting with a ligand. Purple and yellow lines mean ligand bond and non-ligand bond, respectively. A green dashed line represents a hydrogen bond and a value on the line is its length. A red arc represents non-ligand residues involved in hydrophobic contacts. (cont.)

k

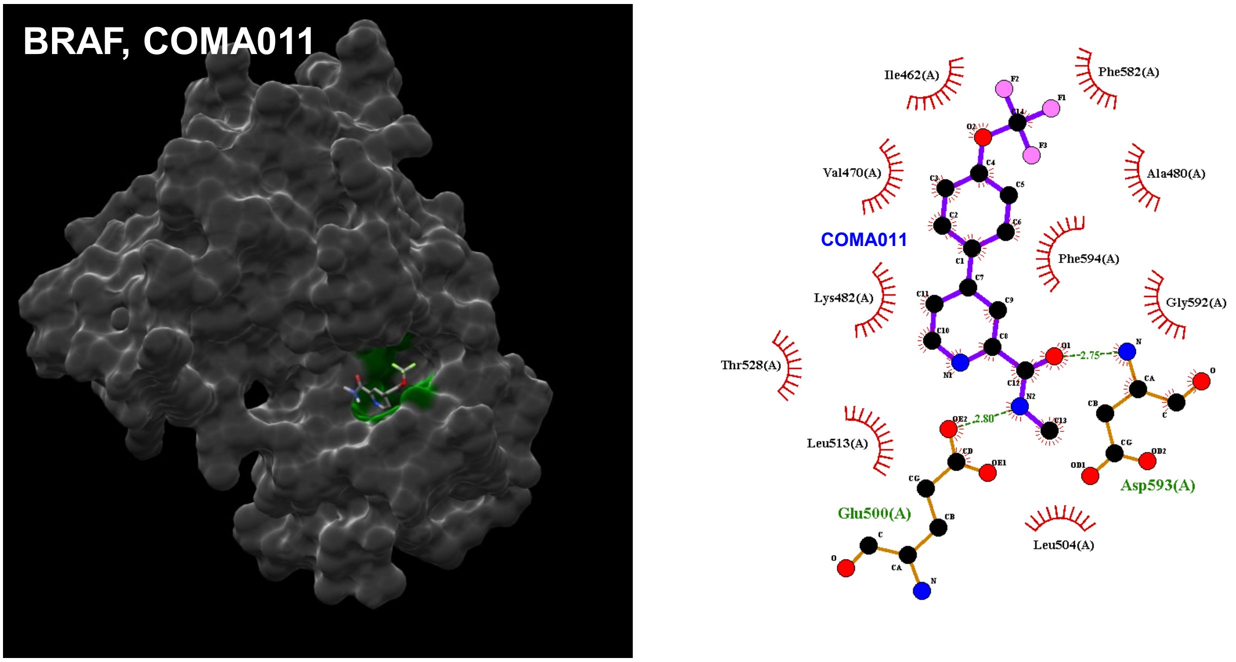

l

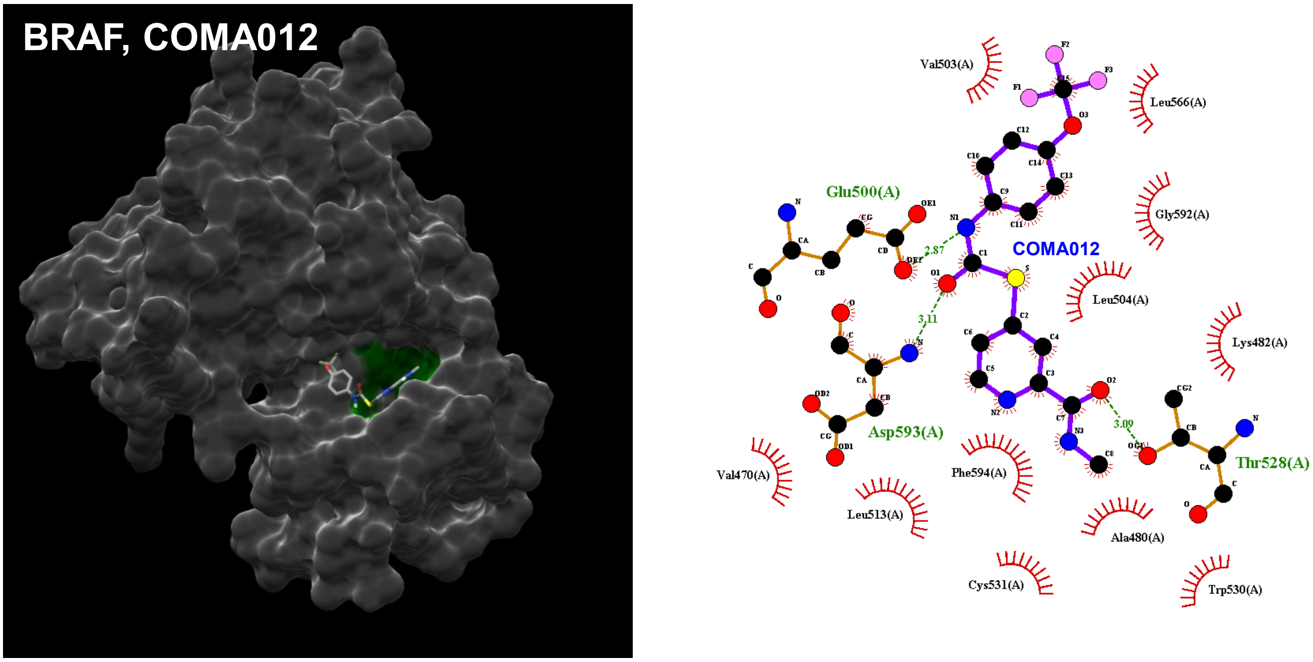

Figure S4. Docking simulation analysis of candidates. (Left) Graphics for the 3D structure of protein-ligand complex. A green surface region represents protein-ligand contacts. (Right) Diagrams of residues interacting with a ligand. Purple and yellow lines mean ligand bond and non-ligand bond, respectively. A green dashed line represents a hydrogen bond and a value on the line is its length. A red arc represents non-ligand residues involved in hydrophobic contacts. (cont.)

m

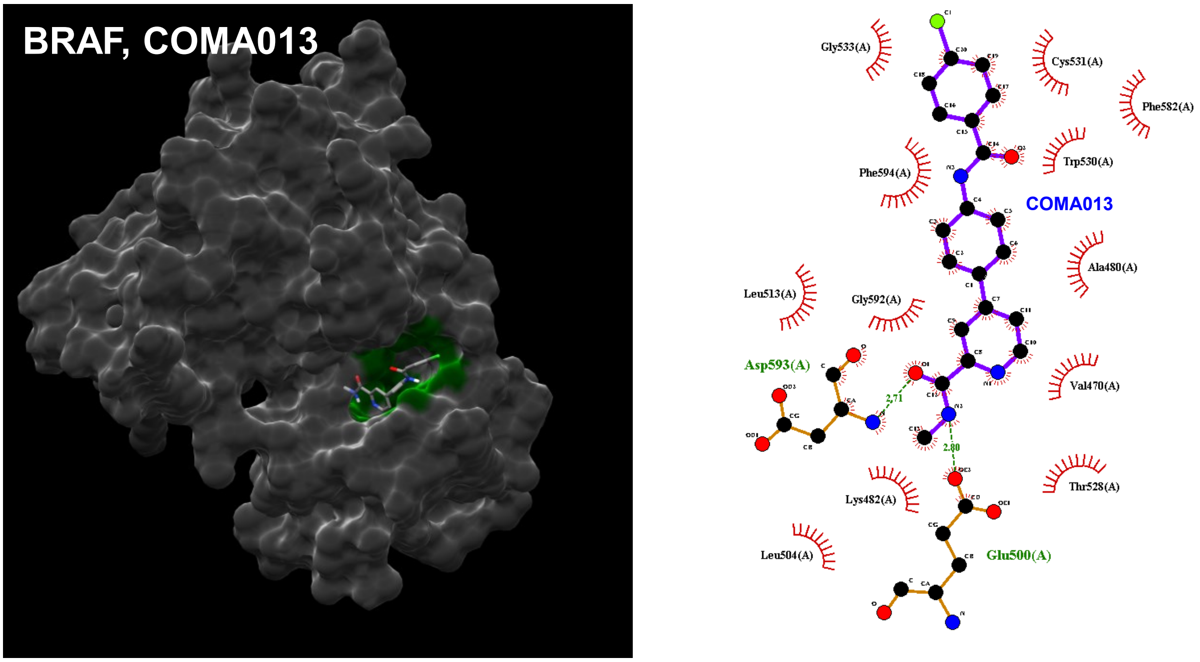

n

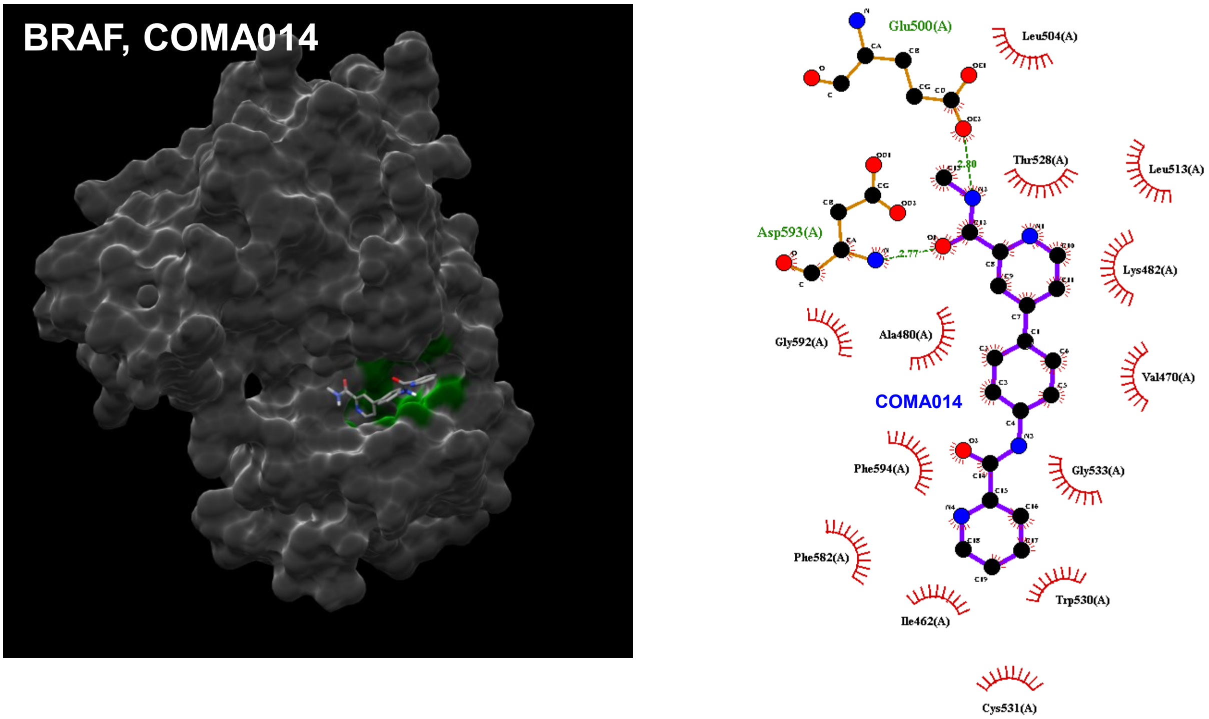

Figure S4. Docking simulation analysis of candidates. (Left) Graphics for the 3D structure of protein-ligand complex. A green surface region represents protein-ligand contacts. (Right) Diagrams of residues interacting with a ligand. Purple and yellow lines mean ligand bond and non-ligand bond, respectively. A green dashed line represents a hydrogen bond and a value on the line is its length. A red arc represents non-ligand residues involved in hydrophobic contacts. (cont.)

o

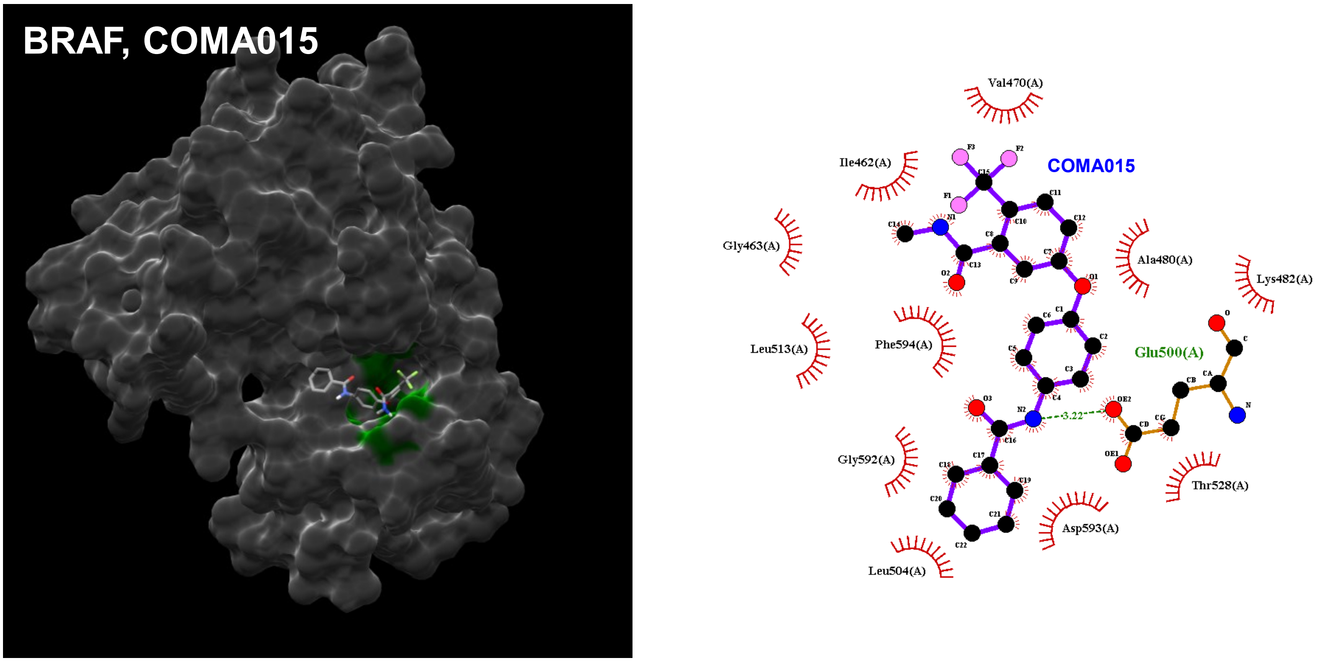

p

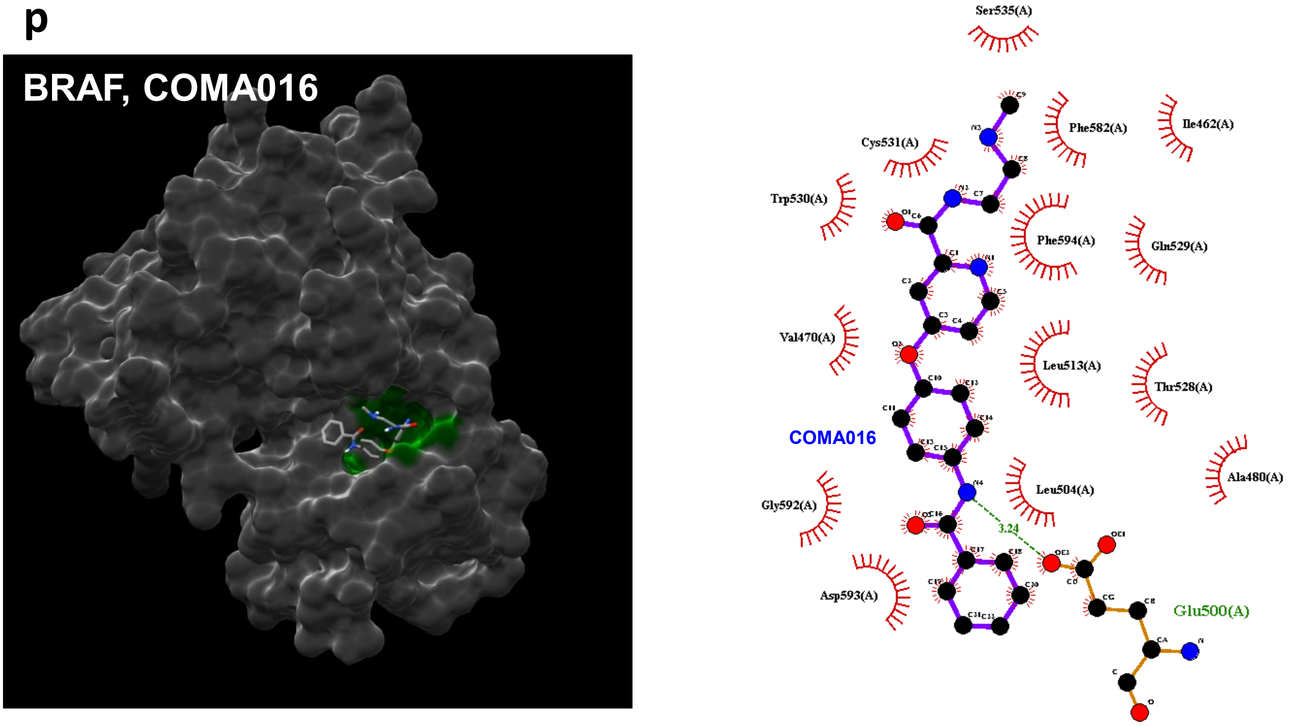

Figure S4. Docking simulation analysis of candidates. (Left) Graphics for the 3D structure of protein-ligand complex. A green surface region represents protein-ligand contacts. (Right) Diagrams of residues interacting with a ligand. Purple and yellow lines mean ligand bond and non-ligand bond, respectively. A green dashed line represents a hydrogen bond and a value on the line is its length. A red arc represents non-ligand residues involved in hydrophobic contacts. (cont.)

q

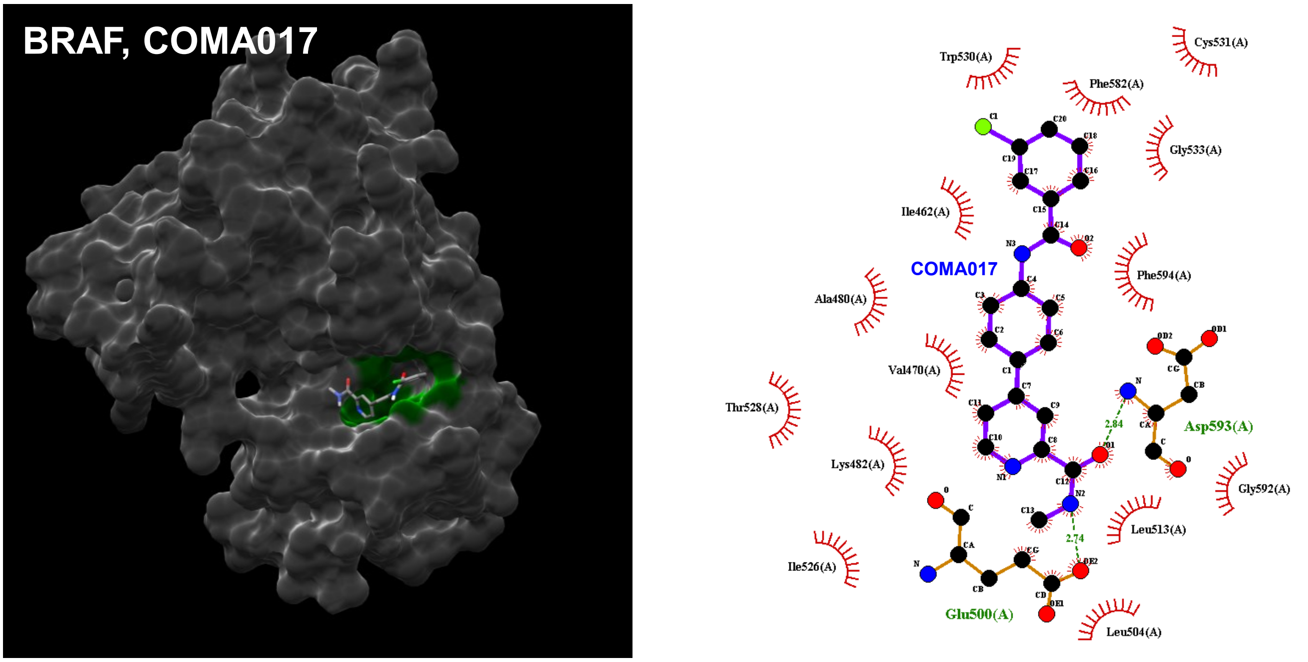

r

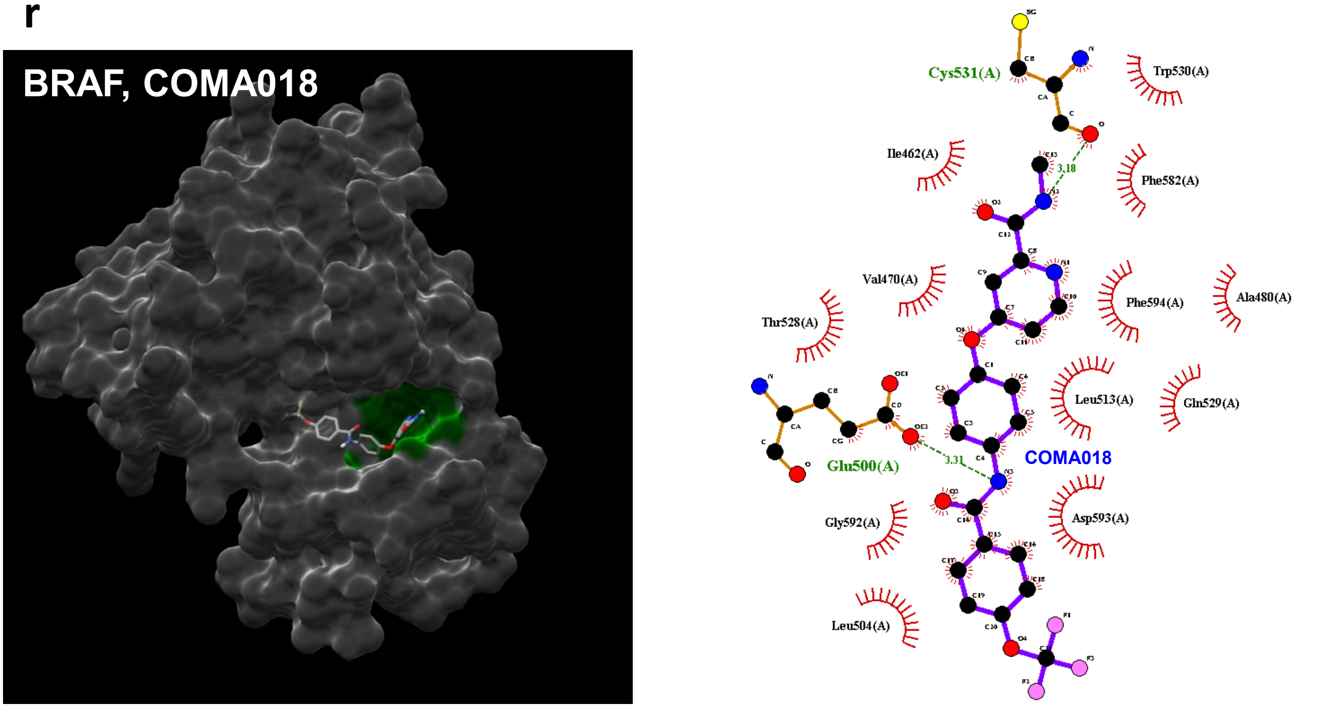

Figure S4. Docking simulation analysis of candidates. (Left) Graphics for the 3D structure of protein-ligand complex. A green surface region represents protein-ligand contacts. (Right) Diagrams of residues interacting with a ligand. Purple and yellow lines mean ligand bond and non-ligand bond, respectively. A green dashed line represents a hydrogen bond and a value on the line is its length. A red arc represents non-ligand residues involved in hydrophobic contacts. (cont.)

s

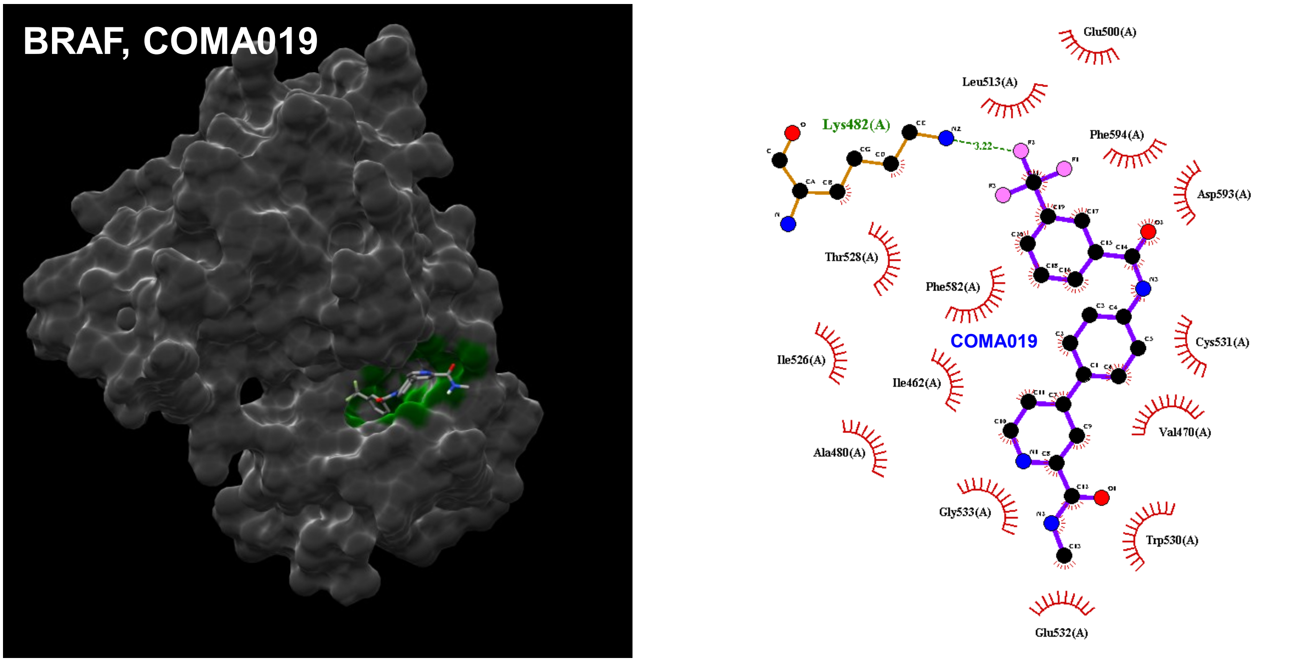

t

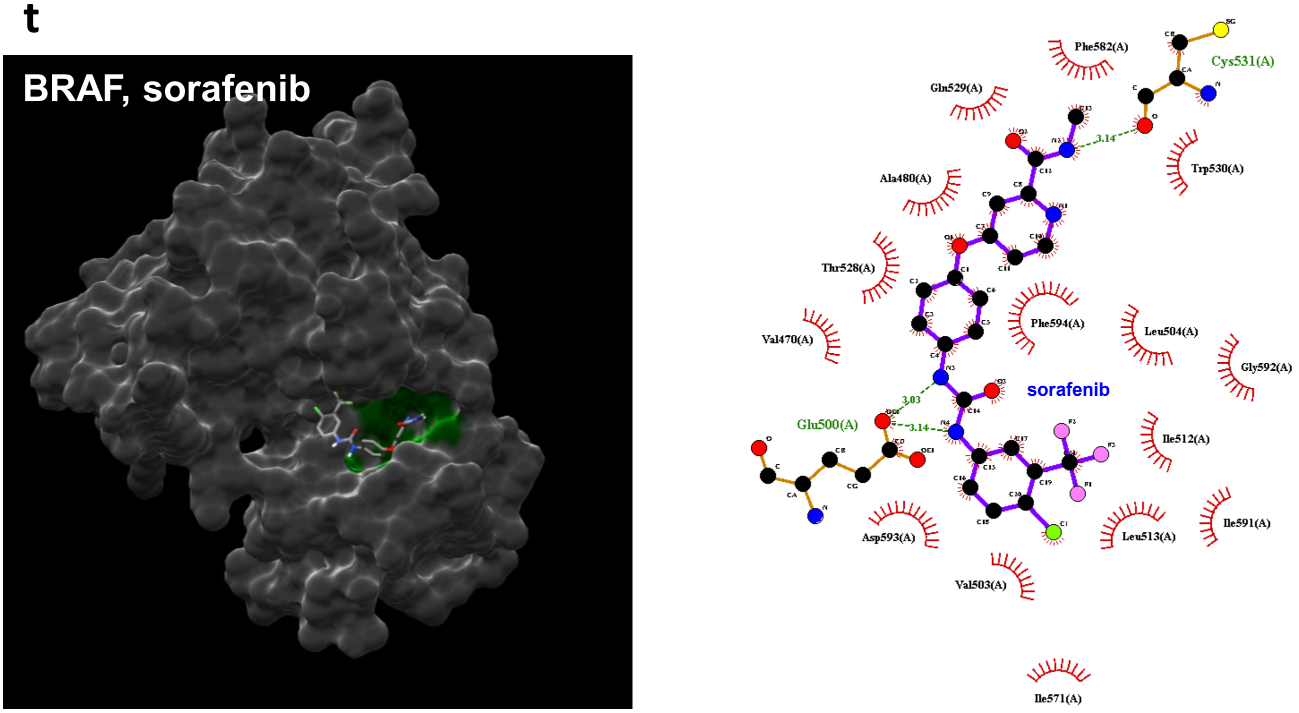

Figure S4. Docking simulation analysis of candidates. (Left) Graphics for the 3D structure of protein-ligand complex. A green surface region represents protein-ligand contacts. (Right) Diagrams of residues interacting with a ligand. Purple and yellow lines mean ligand bond and non-ligand bond, respectively. A green dashed line represents a hydrogen bond and a value on the line is its length. A red arc represents non-ligand residues involved in hydrophobic contacts.

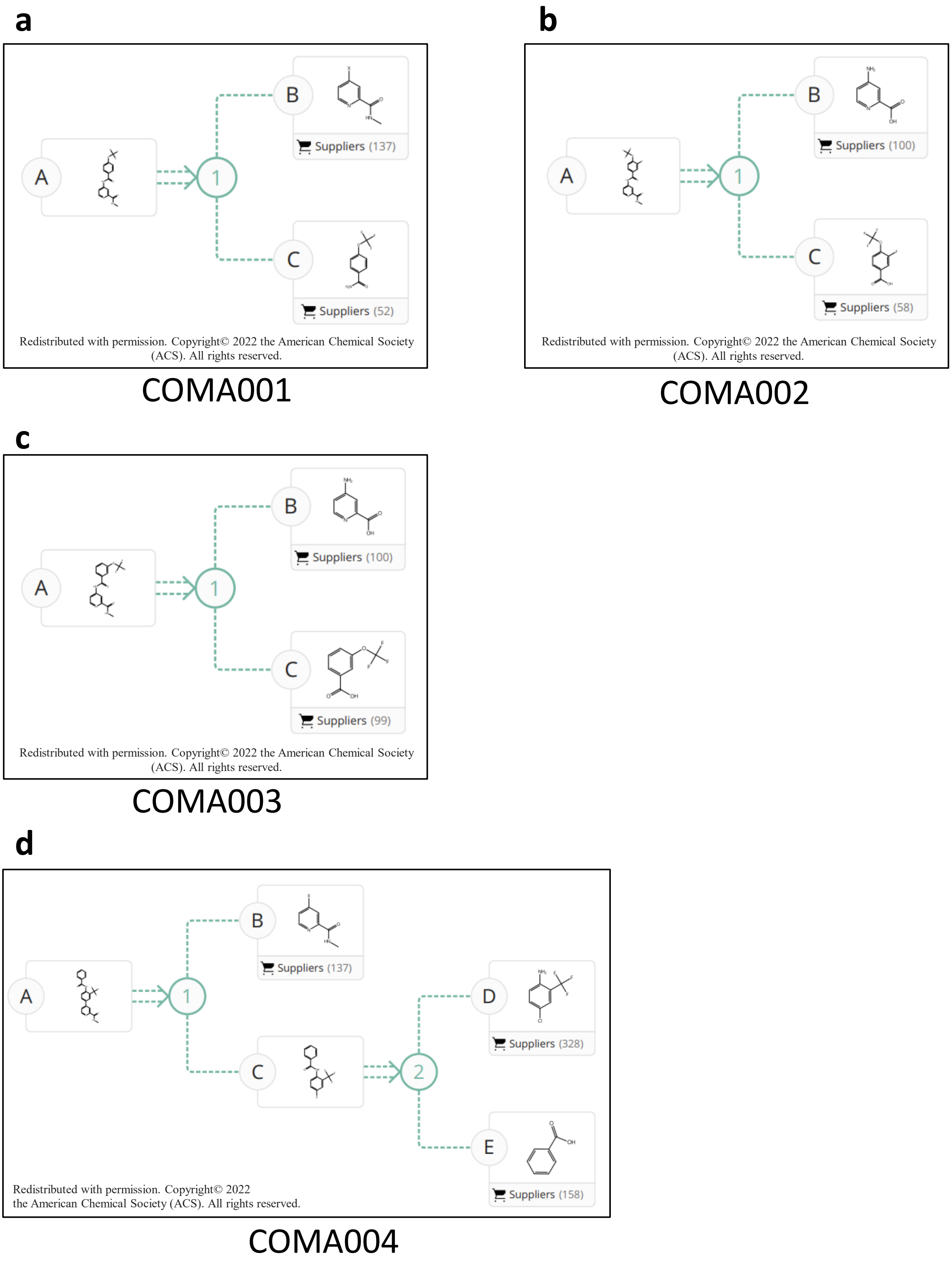

Figure S5. Retrosynthesis analysis of candidates. Green and purple arrows represent predicted and experimental steps, respectively. (cont.)

e

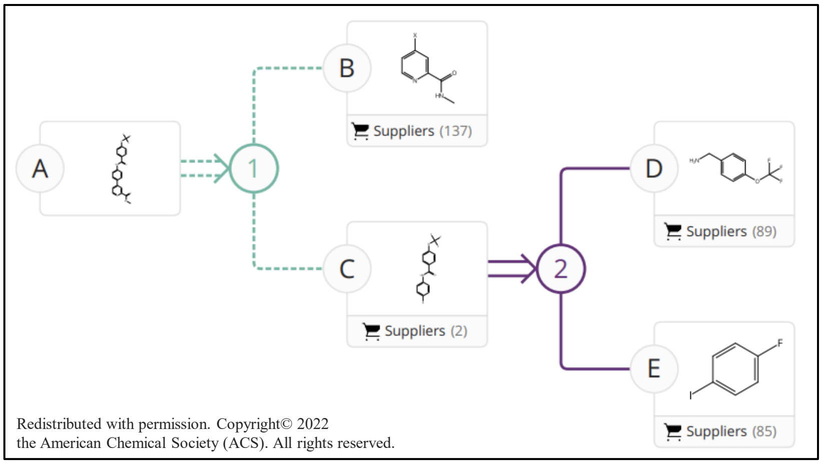

COMA005

f

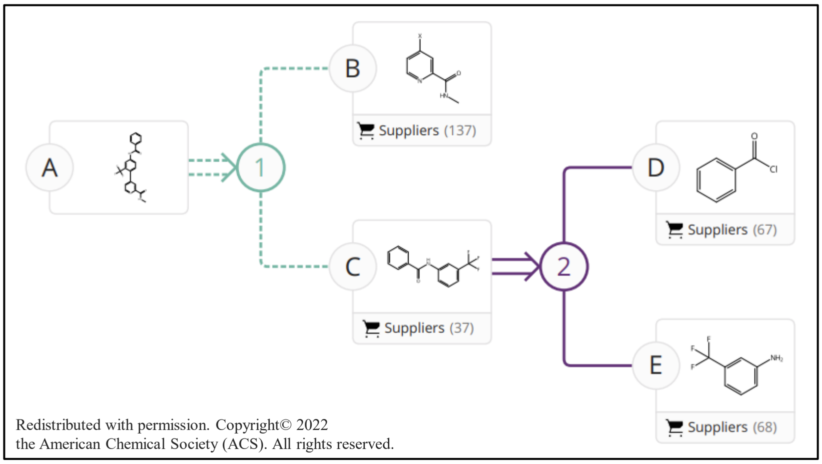

COMA006

g

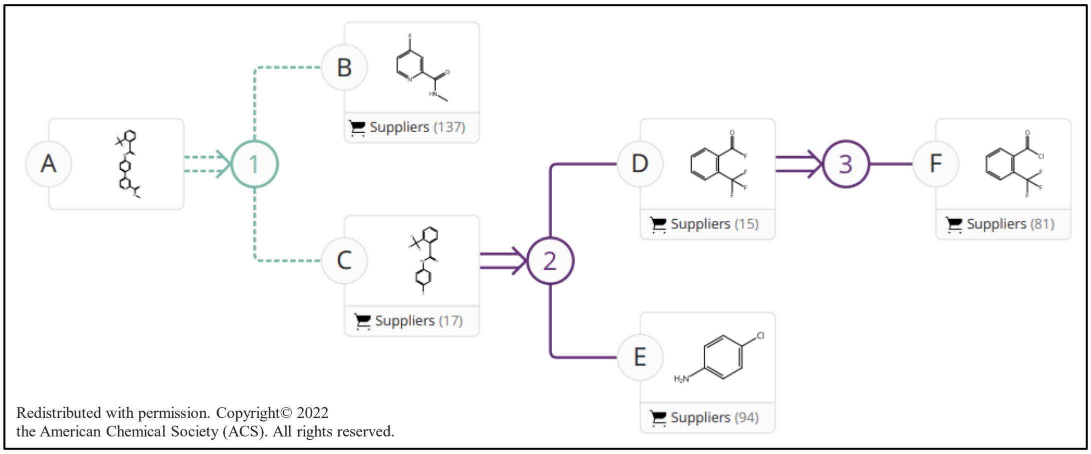

COMA007

Figure S5. Retrosynthesis analysis of candidates. Green and purple arrows represent predicted and experimental steps, respectively. (cont.)

h

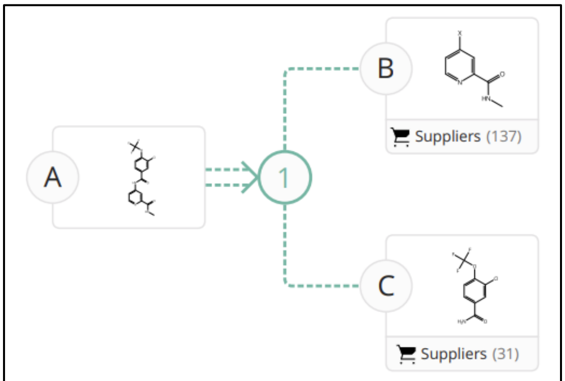

Redistributed with permission. Copyright© 2022 the American Chemical Society (ACS). All rights reserved.

COMA008

i

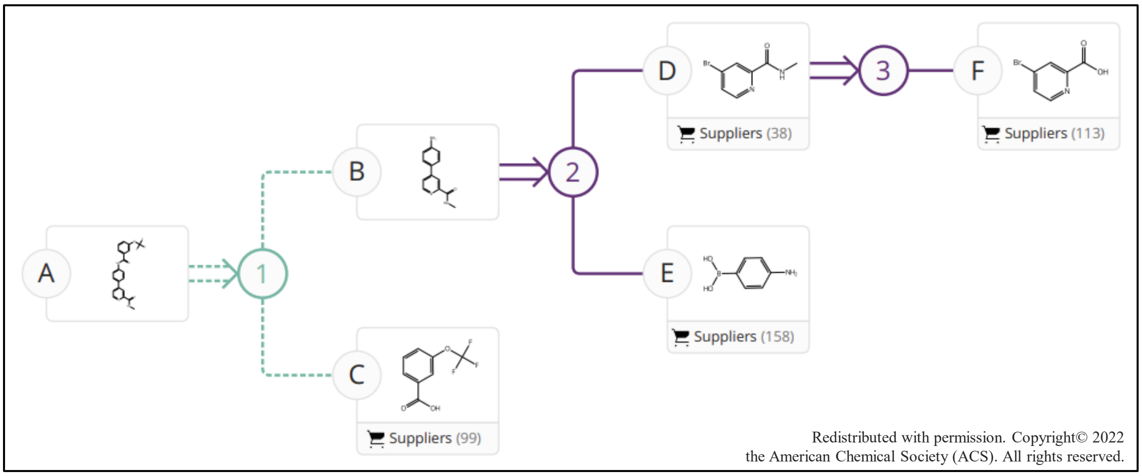

Redistributed with permission. Copyright© 2022 the American Chemical Society (ACS). All rights reserved.

COMA009

j

Redistributed with permission. Copyright© 2022 the American Chemical Society (ACS). All rights reserved.

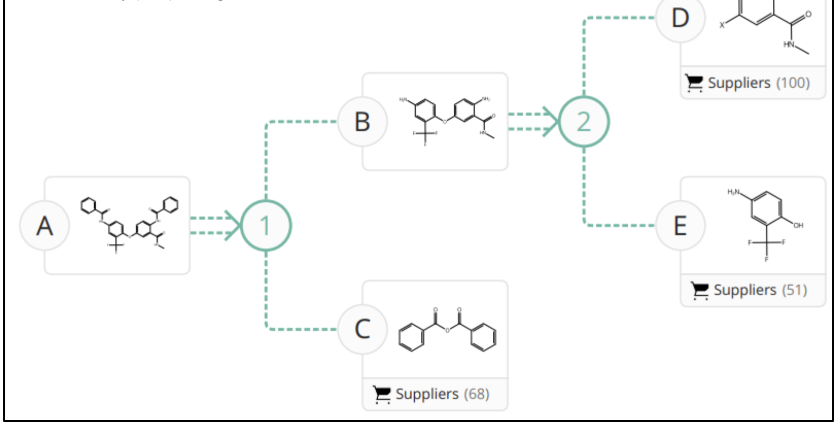

COMA010

Figure S5. Retrosynthesis analysis of candidates. Green and purple arrows represent predicted and experimental steps, respectively. (cont.)

k

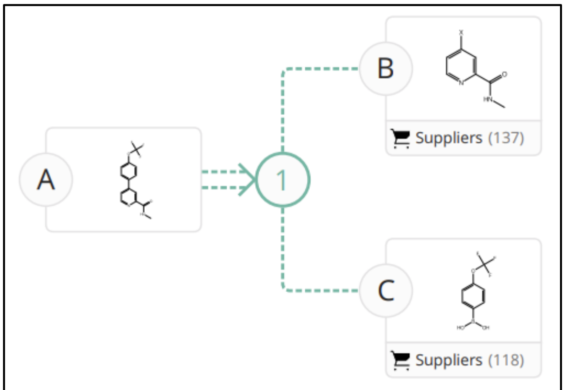

Redistributed with permission. Copyright© 2022 the American Chemical Society (ACS). All rights reserved.

COMA011

l

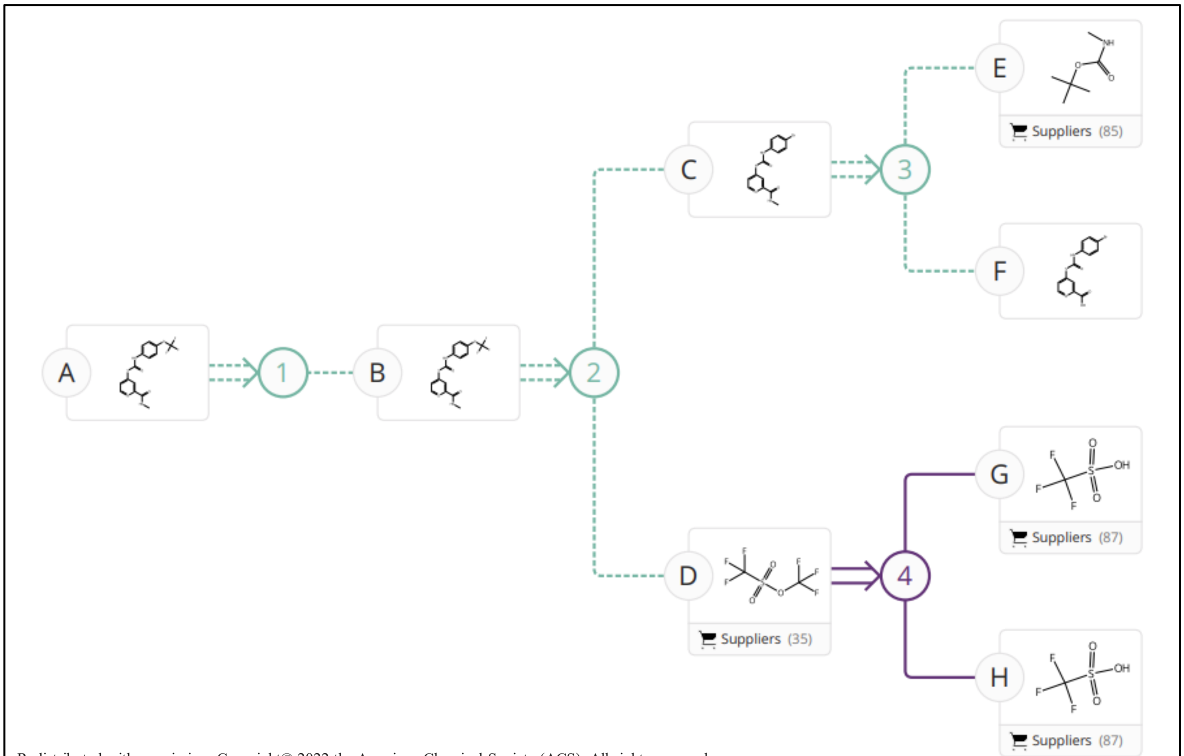

Redistributed with permission. Copyright© 2022 the American Chemical Society (ACS). All rights reserved.

COMA012

Figure S5. Retrosynthesis analysis of candidates. Green and purple arrows represent predicted and experimental steps, respectively. (cont.)

m

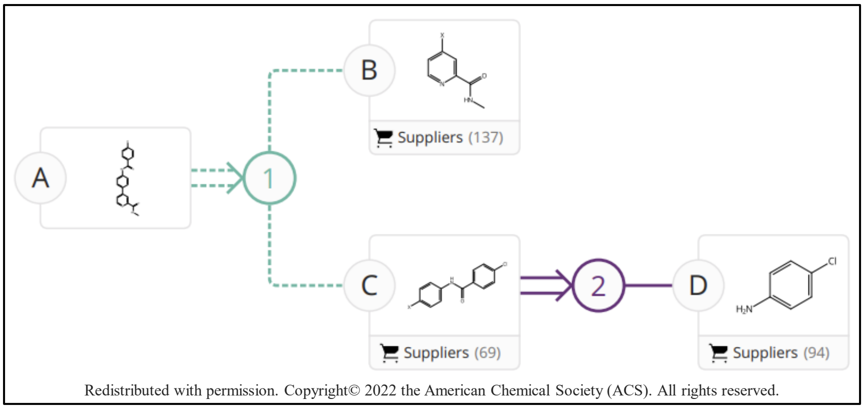

COMA013

n

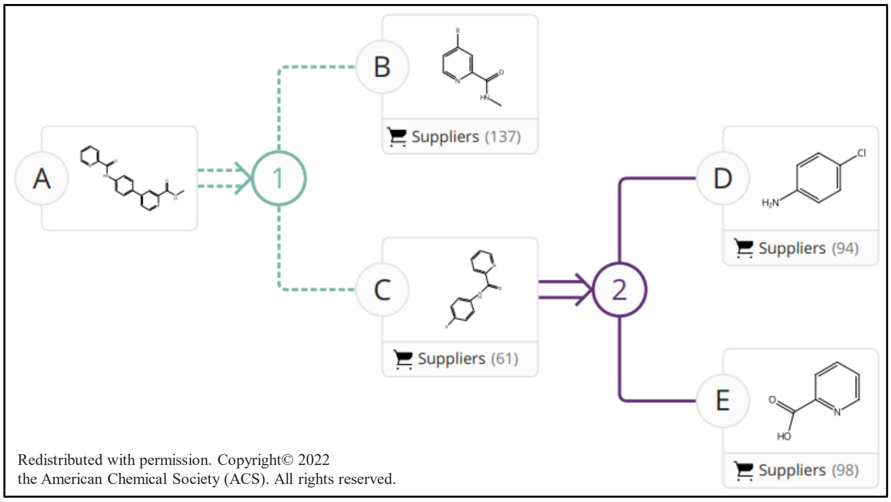

COMA014

o

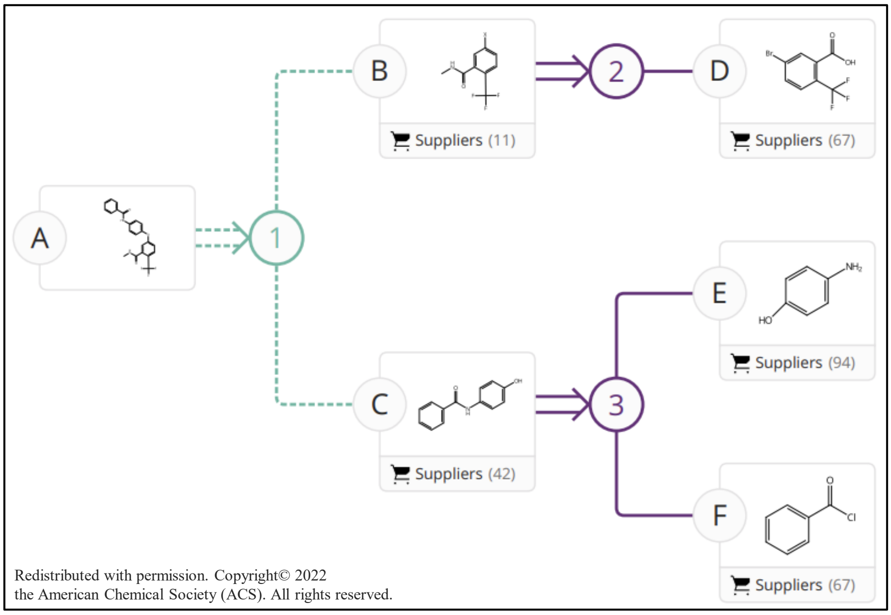

COMA015

Figure S5. Retrosynthesis analysis of candidates. Green and purple arrows represent predicted and experimental steps, respectively. (cont.)

p

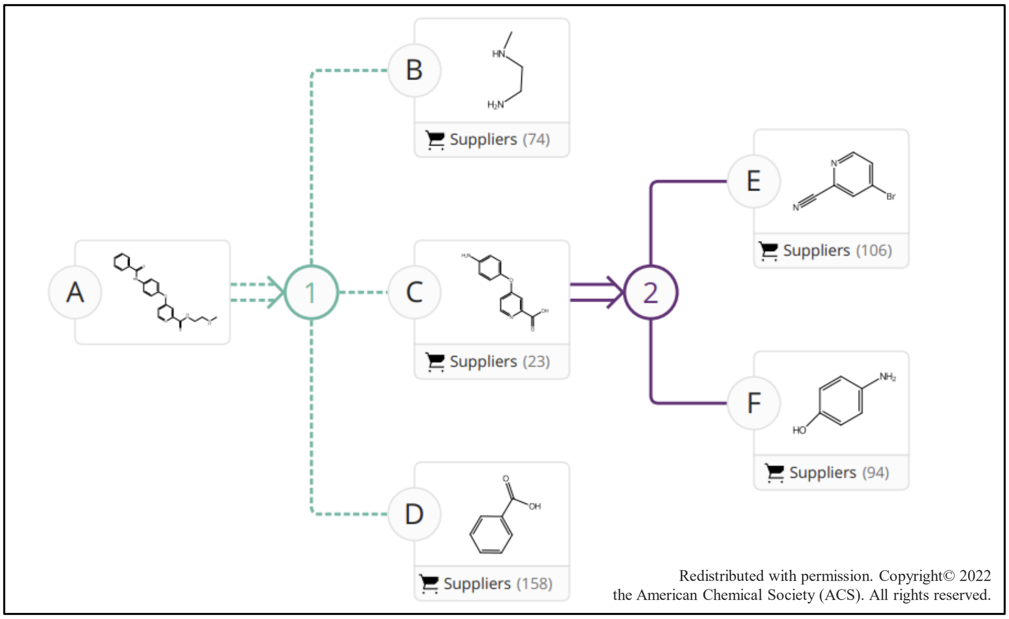

COMA016

q

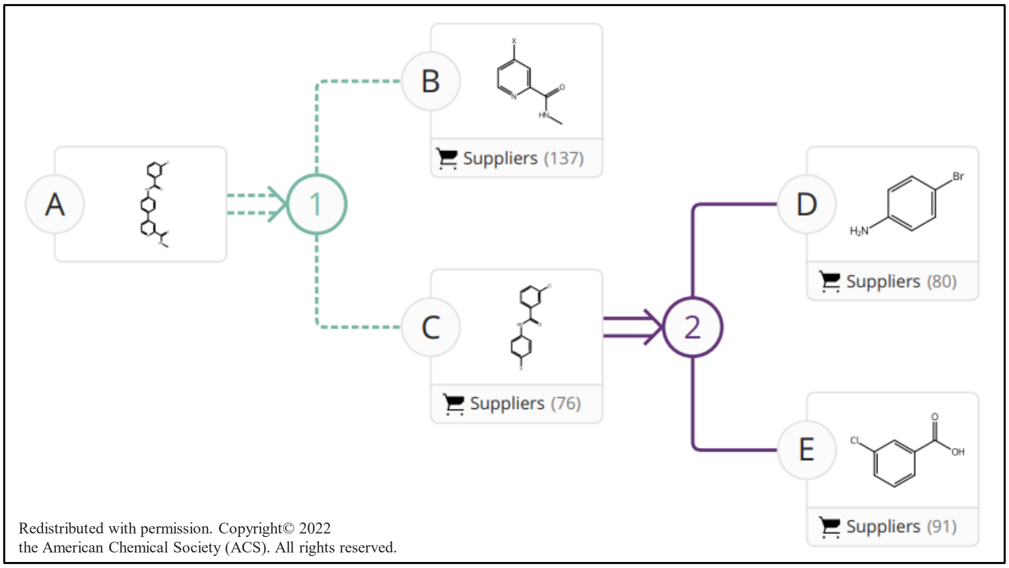

COMA017

Figure S5. Retrosynthesis analysis of candidates. Green and purple arrows represent predicted and experimental steps, respectively. (cont.)

r

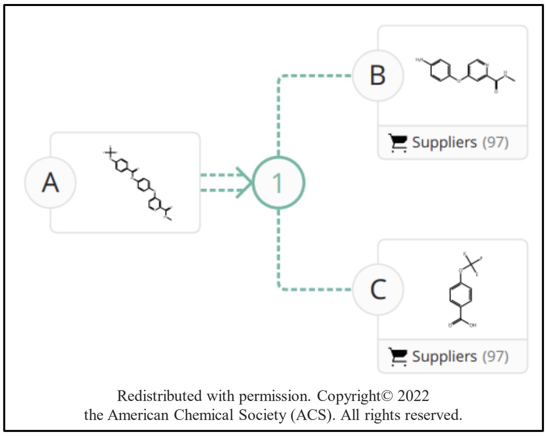

COMA018

s

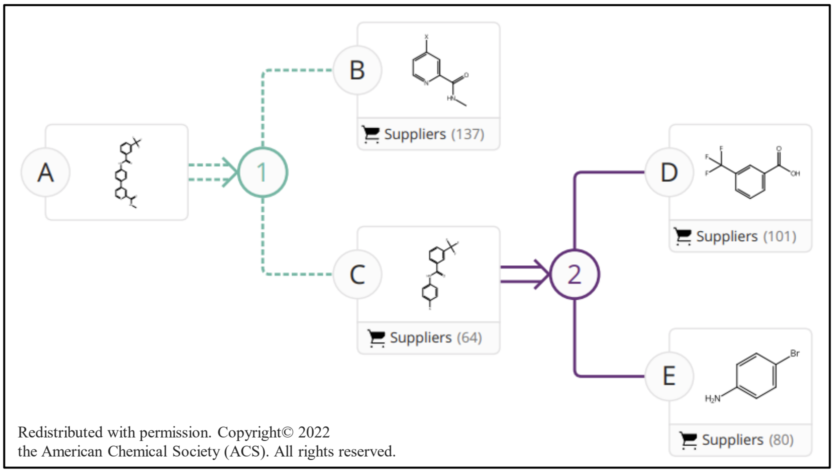

COMA019

Figure S5. Retrosynthesis analysis of candidates. Green and purple arrows represent predicted and experimental steps, respectively.

a

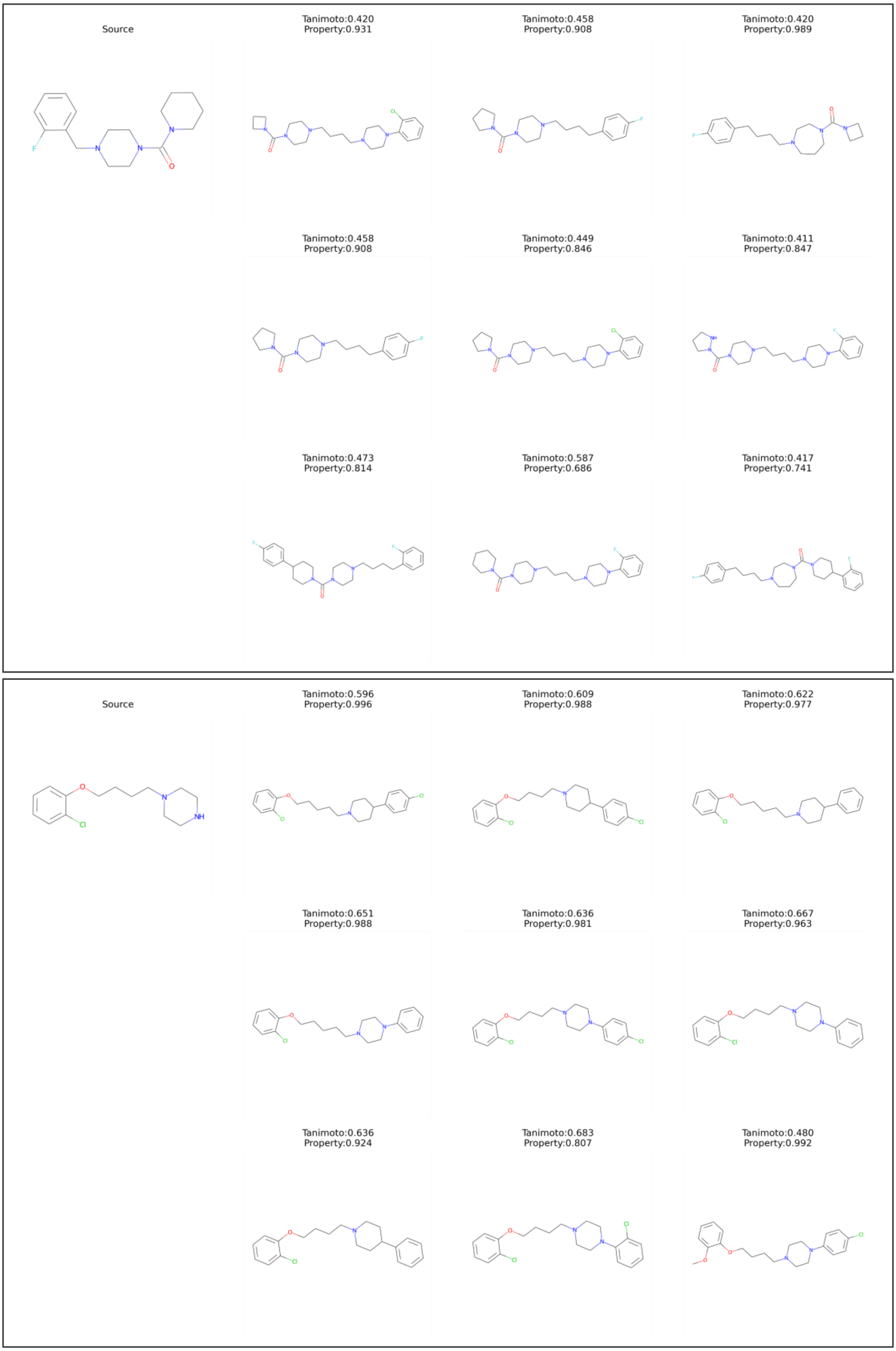

Figure S6. Examples of molecular translation via COMA. a, DRD2 results. **b**, QED results. **c**, Penalized LogP04 results. **d**, Penalized LogP06 results.

b

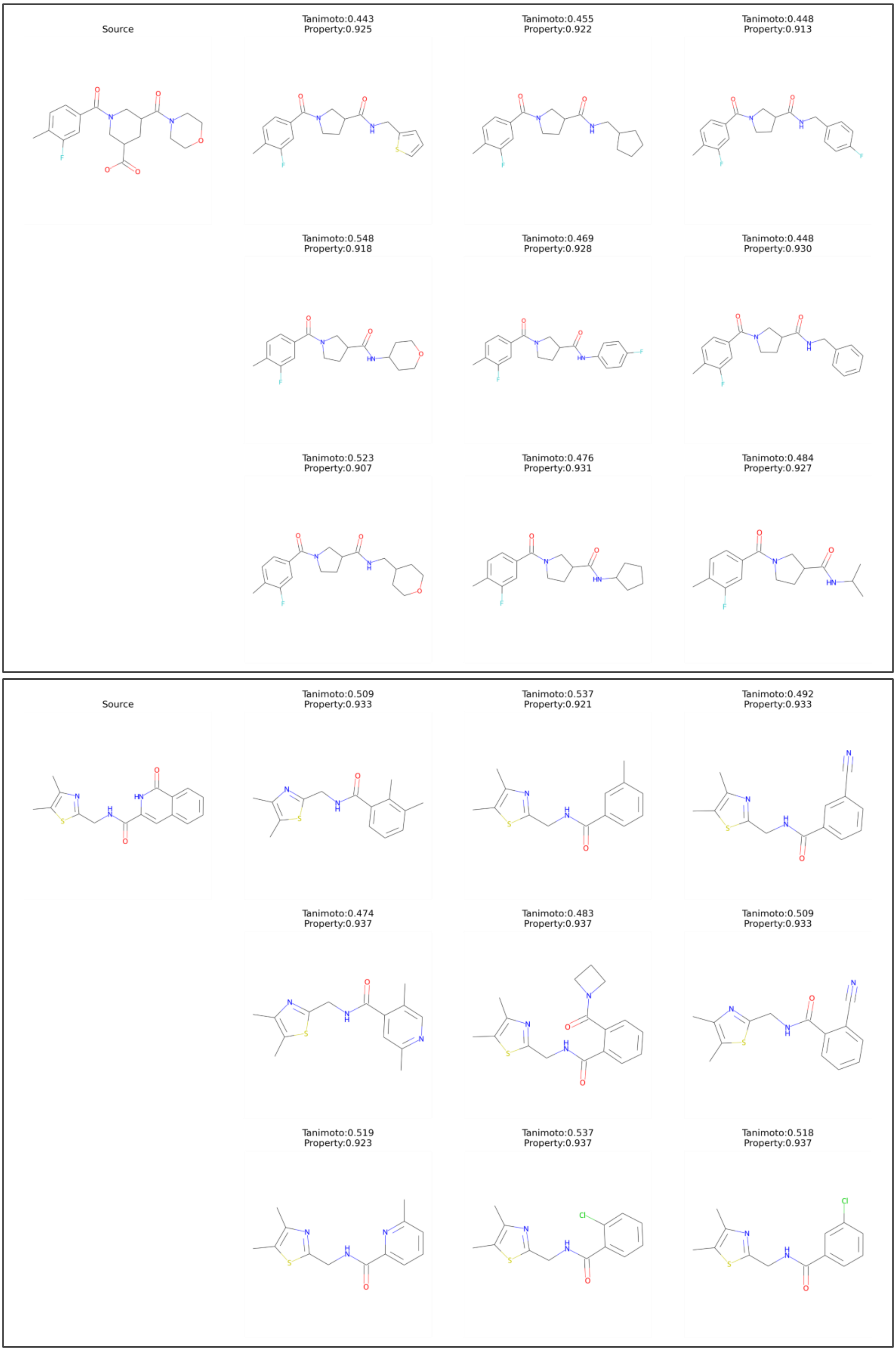

Figure S6. Examples of molecular translation via COMA. a, DRD2 results. **b**, QED results. **c**, Penalized LogP04 results. **d**, Penalized LogP06 results.

c

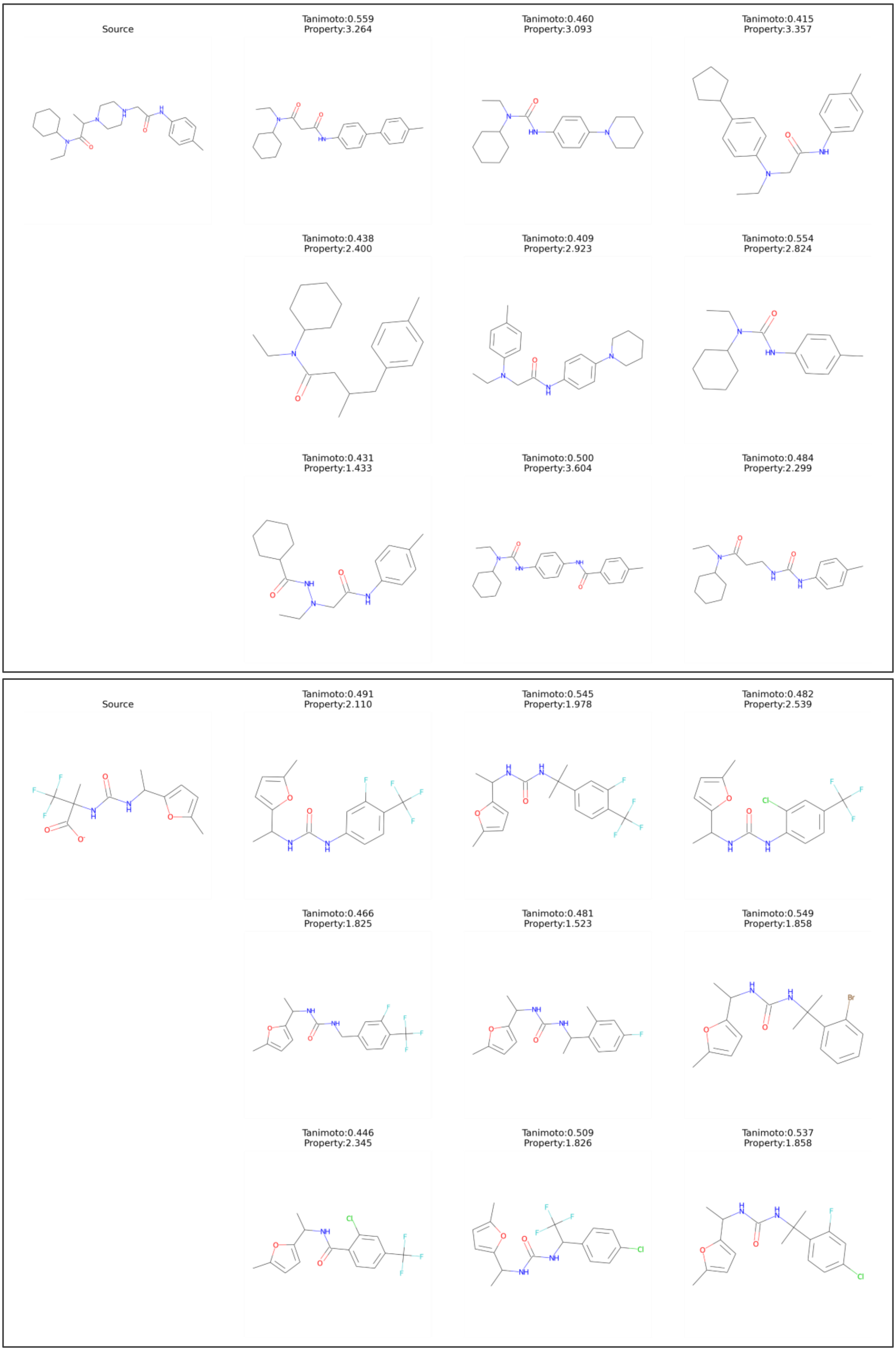

Figure S6. Examples of molecular translation via COMA. a, DRD2 results. **b**, QED results. **c**, Penalized LogP04 results. **d**, Penalized LogP06 results.

d

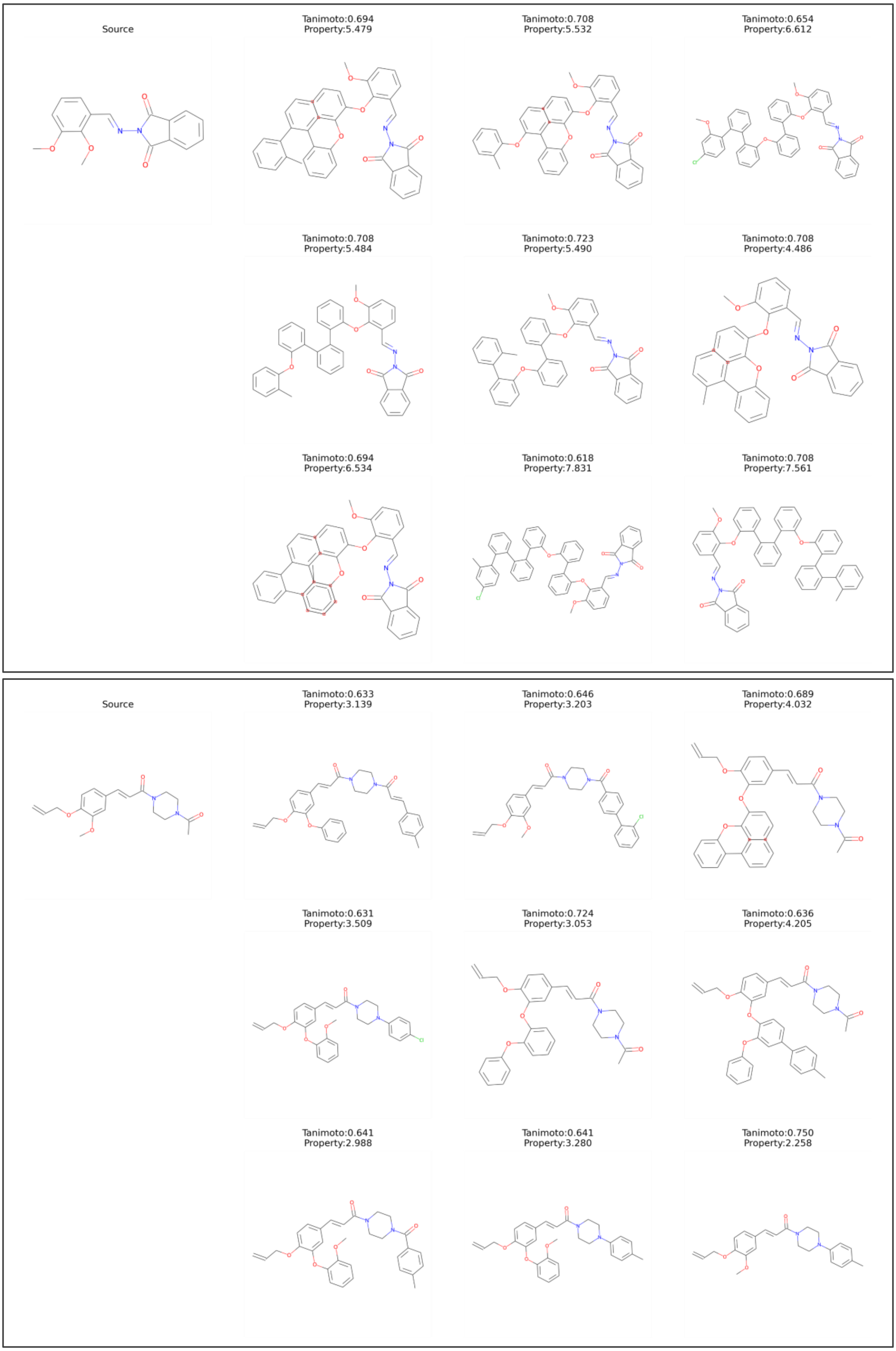

Figure S6. Examples of molecular translation via COMA. a, DRD2 results. **b**, QED results. **c**, Penalized LogP04 results. **d**, Penalized LogP06 results.
